# Diverse RNA viruses of parasitic nematodes can elicit antibody responses in vertebrate hosts

**DOI:** 10.1038/s41564-024-01796-6

**Published:** 2024-09-04

**Authors:** Shannon Quek, Amber Hadermann, Yang Wu, Lander De Coninck, Shrilakshmi Hegde, Jordan R. Boucher, Jessica Cresswell, Ella Foreman, Andrew Steven, E. James LaCourse, Stephen A. Ward, Samuel Wanji, Grant L. Hughes, Edward I. Patterson, Simon C. Wagstaff, Joseph D. Turner, Rhys H. Parry, Alain Kohl, Eva Heinz, Kenneth Bentum Otabil, Jelle Matthijnssens, Robert Colebunders, Mark J. Taylor

**Affiliations:** 1 Centre for Neglected Tropical Diseases, Department of Tropical Disease Biology, Liverpool School of Tropical Medicine, Liverpool, UK; 2 Global Health Institute, University of Antwerp, Antwerp, Belgium; 3 Laboratory of Viral Metagenomics, Clinical and Epidemiological Virology, Rega Institute, Department of Microbiology, Immunology and Transplantation, KU Leuven, Leuven, Belgium; 4 Parasite and Vector Biology Research Unit, Department of Microbiology and Parasitology, Faculty of Science, University of Buea, Buea, Cameroon; 5 Research Foundation for Tropical Diseases and the Environment (REFOTDE), Buea, Cameroon; 6 Centre for Neglected Tropical Diseases, Departments of Tropical Disease Biology and Vector Biology, Liverpool School of Tropical Medicine, Liverpool, UK; 7 Department of Biological Sciences, Brock University, St Catharines, Ontario Canada; 8 School of Chemistry and Molecular Biosciences, The University of Queensland, Brisbane, Queensland Australia; 9 Departments of Vector Biology and Clinical Sciences, Liverpool School of Tropical Medicine, Liverpool, UK; 10 Consortium for Neglected Tropical Diseases and One Health, Department of Biological Sciences, University of Energy and Natural Resources, Sunyani, Ghana; 11 Present Address: Strathclyde Institute of Pharmacy & Biomedical Sciences, University of Strathclyde, Glasgow, UK

**Keywords:** Viral reservoirs, Parasite biology, Virus-host interactions, Bioinformatics, RNAi

## Abstract

Parasitic nematodes have an intimate, chronic and lifelong exposure to vertebrate tissues. Here we mined 41 published parasitic nematode transcriptomes from vertebrate hosts and identified 91 RNA viruses across 13 virus orders from 24 families in ~70% (28 out of 41) of parasitic nematode species, which include only 5 previously reported viruses. We observe widespread distribution of virus–nematode associations across multiple continents, suggesting an ancestral acquisition event and host–virus co-evolution. Characterization of viruses of *Brugia malayi* (BMRV1) and *Onchocerca volvulus* (OVRV1) shows that these viruses are abundant in reproductive tissues of adult parasites. Importantly, the presence of BMRV1 RNA in *B. malayi* parasites mounts an RNA interference response against BMRV1 suggesting active viral replication. Finally, BMRV1 and OVRV1 were found to elicit antibody responses in serum samples from infected jirds and infected or exposed humans, indicating direct exposure to the immune system.

## Abstract (web summary)

Transcriptome mining, phylogenetic analysis, bioimaging and serology experiments reveal a widespread diverse virome present in ~70% of parasitic nematodes, such as *Brugia malayi* and *Onchocerca volvulus*, where some viruses are abundant in the reproductive tract and are associated with seropositivity in vertebrate hosts.

## Main

Humans and animals are frequently infected with multiple species of parasitic nematodes^1–3^ and suffer from chronic, lifelong infections and exposure to continuous reinfection^4^. Such infections impose a substantial health burden on billions of people, impacting their health, quality of life and economic productivity. Medically important parasitic nematodes infect over one billion people, resulting in up to 7.53 million disability-adjusted life years globally^5^. Prominent examples include intestinal species such as *Ascaris lumbricoides* and *Trichuris trichiura*^4^, which infect an estimated 511 and 412 million people, respectively^5^, as well as the hookworms *Necator americanus*, *Ancylostoma duodenale* and *Ancylostoma ceylanicum*, which collectively infect up to 186 million people globally^5^. Infected individuals can suffer from severe abdominal discomfort, bloody diarrhoea, stunted development and anaemia. Other examples include the filarial nematodes *Wuchereria bancrofti* and *Brugia malayi*, the causative agents of lymphatic filariasis that infect up to 96 million people globally^5,6^, and *Onchocerca volvulus*, which infects up to 21 million people^5^. In the case of *O. volvulus*, recent estimates indicate that 14.6 million are afflicted with skin disease and 1.15 million with blindness^7^. Furthermore, there has been increasing recognition of a disease known as onchocerciasis-associated epilepsy (OAE), occurring in children and adolescents in onchocerciasis meso- and hyperendemic foci across sub-Saharan Africa^8^. This condition manifests as a variety of epileptic seizures, including uncontrollable repeated head nodding (‘nodding syndrome’), as well as severe stunting, delayed puberty and impaired mental health (Nakalanga syndrome)^9^. OAE has been epidemiologically linked to infection with *O. volvulus*^10^, but the pathogenesis has yet to be identified^8^.

A variety of viruses can be found infecting several human parasitic protozoa, including *Plasmodium vivax*, *Trichomonas vaginalis* and *Cryptosporidium parvum*^11,12^. Viruses infecting *Leishmania* sp. have been studied in great detail^13^ and can increase disease severity, parasite prevalence and potentially the incidence rates of both drug resistance and mucocutaneous leishmaniasis^14,15^. RNA virus infections have been identified in plant-parasitic nematodes^16^, parasitic flatworms^17,18^ and free-living nematodes^17,19,20^, although the impact of viral infections on the biology of the worms is largely unknown.

Here we analysed the transcriptomes of 41 parasitic nematode species infecting humans and animals and discovered 91 virus or virus-like genomic sequences across 28 species. We further characterize the viruses infecting *B. malayi* and *O. volvulus*, describing their genomic diversity, geographic spread, phylogeny, abundance throughout different developmental stages, tissue tropism, localization and vertebrate host serology. Finally, we show that an RNA interference (RNAi) response is induced in *B. malayi* against BMRV1, providing evidence for active viral replication.

## Results

### Viruses in human- and animal-parasitic nematodes

We mined publicly available transcriptomes (Supplementary Table 1) available in the Sequence Read Archive databanks^3^ for RNA viral sequences. We screened 52 different BioProjects that covered 41 species of parasitic nematodes (Extended Data Table 1). Approximately 70% (28 out of 41) showed evidence for a total of 91 virus or virus-like sequences, based on the presence of RNA-dependent RNA polymerases (RdRPs). Eight of these sequences were identified only after removal of host transcript reads. The RdRP sequences were identified as either complete (49) or partial (42) based on comparisons with their closest relatives in existing databases and the presence of complete open reading frames. Viruses were distributed across 13 virus orders from 24 families, indicating an extensive diversity of virus taxa within parasitic nematodes (Extended Data Table 1). The most common virus families were the *Totiviridae* (36 genomes), *Partitiviridae* (8 genomes), *Rhabdoviridae* and *Lispiviridae* (6 genomes each). A total of 20 sequences were predicted members of virus families characterized by segmented genomes (Supplementary Table 1). Subsequent validation showed no evidence of host contamination, with all but two sequences showing at least one viral marker gene (as defined by CheckV (ref. ^21^)). These exceptions (*Onchocerca ochengi* RNA Virus 5 and *Onchocerca volvulus* RNA Virus 3) were validated separately via manual BlastX and BlastN searches as being related to unclassified members of the Bunyavirales order. Predicted completeness of the sequences varied from 0.82% to 100%, with 28 sequences predicted to be <50% complete (Supplementary Table 2). We used CheckV (ref. ^21^) to perform additional manual inspection of complete virus genome organization, open reading frames and comparisons against closest relatives. This identified no frameshifts or nonsense mutations, and no indication of contamination with host parasite genes and transcripts. Taken together, these factors indicate that the complete virus sequences identified are likely authentic nematode viruses.

In addition to identifying four previously unreported RNA viruses and one previously predicted from a *Toxocara canis* cDNA library, two endogenous viral element sequences were identified in published *T. canis* genomes via BLAST search. Both sequences encode RdRPs and are predicted to be members of the *Partitiviridae* family, and are closely related variants that share 88% nucleotide identity. Both sequences show high similarity to the *T. canis* protein KHN74396.1 (genome locus tag Tcan_15821, >88% nucleotide identity for both sequences). It is interesting to note that both sequences show high amino acid similarity (>75%) to the Wuhan large pig roundworm virus in *Ascaris suum* (accession AVV63192.1). Such endogenous viral elements have been reported within nematode genomes previously, such as the Atlas virus (family *Belpaoviridae*) in *A. ceylanicum*^22^. Our analysis did not identify the full genome sequence for the Atlas virus, but we were able to identify multiple fragments, all of which showed >99% nucleotide similarity to the Atlas virus. Taken together, the fragments covered 70.1% of the Atlas virus genome in *A. ceylanicum*.

As the transcriptome data were derived from a variety of different sources, and the parasites isolated from vertebrate hosts, it is possible that some of the identified virus sequences may be contamination (derived from the vertebrate host, environment or reagents), rather than genuinely infecting the nematode. Of the 91 sequences identified, five were identified as likely contaminants based on their closest relatives: three were predicted to be members of the *Fiersviridae* bacteriophage family, and one each was predicted to be a member of the families *Closteoviridae* and *Chrysoviridae* (infecting plants, fungi and potentially insects). Aside from these, the majority of identified viruses (Extended Data Table 1) have family members previously identified as infecting other nematodes^23^, including the most frequently identified virus families. Others, such as the *Peribunyaviridae* and *Phenuiviridae* families, while not known to have members infecting nematodes, are members of the larger Bunyavirales order that has several unclassified members that infect nematodes^23^.

### Global spread of viral and nematode ancestral associations

We compared all virus sequences against the NCBI database using BlastX and BlastN, to find sequences that were previously identified. We found that three of the four viruses in *A. suum* parasites isolated from pigs in the United States^24^ were previously identified from parasites isolated from pigs in China during an earlier survey of the invertebrate virome^23^. In all three cases, amino acid sequences from this study showed >98% sequence similarity to previously published viral genomes. This includes Hubei rhabdo-like virus 9 (GenBank accession KX884448.1), Hubei toti-like virus 12 (KX882956.1) and the Wuhan large pig roundworm virus 1 (KX884219.1/KX884220.1). This indicates that viral infections in *A. suum* have a widespread distribution, possibly from ancestral acquisition and co-evolution, or from the global pig trade. In addition, a study in Brazil identified the Wuhan large pig roundworm virus 1 in human faecal samples^25^ (MG746618.1/MG746619.1). While the study was unable to identify the presence of an *A. lumbricoides* infection owing to sample preparation methods^25^, this finding suggests that *Ascaris* infections in both pigs and humans can carry the same virus.

We also assembled more complete sequences for two previously published viruses. The first is for an unnamed virus belonging to the *Phenuiviridae* found within *Angiostrongylus cantonensis* (rat lungworm). The virus fragment was originally found during a survey of wild rodents in Southeast Asia^26^ (MT085324.1, with 97% nucleotide sequence similarity covering 50% of the sequence identified in this study). The second is an RNA virus in *T. canis* related to the Jingmenvirus group, which are segmented viruses related to the *Flaviviridae*^27^. The first Jingmenvirus was originally identified in ticks^28^, but a second was simultaneously predicted to exist in *T. canis* cDNA libraries^28,29^, tentatively named Toxocara canis larva agent (TCLA). This previously identified sequence (EU792509.1) showed >98% nucleotide sequence similarity, but covered only 56% of the sequence assembled in this study.

With these exceptions, none of the remaining 86 virus sequences occur in existing databases (all <50% nucleotide sequence similarity). In addition, we observed only two instances in which the same virus could be found across different nematode species. The first is a member of the *Rhabdoviridae*, found in *O. volvulus* and *O. ochengi*, with the proposed name of Onchocerca volvulus RNA Virus 1 (OVRV1). *O. ochengi* is the most closely related species to *O. volvulus*^30^, with the virus sequences sharing 96% nucleotide sequence similarity. The second is a member of the *Totiviridae*, found in eight species of *Trichinella* parasites (Extended Data Table 1). These sequences show 77% to 99% nucleotide sequence similarity between them, indicating either the same or closely related variants. To illustrate this relationship, we constructed a phylogenetic tree for these *Totiviridae* and compared it with a single-copy orthologue gene tree for their *Trichinella* hosts. This comparison indicated early acquisition and subsequent co-divergence events between virus and parasite host (Supplementary Fig. 1). Exceptions exist for *Trichinella* sp. T6 and sp. T9, and *Trichinella nativa*, which show potential evidence of horizontal transfer of virus.

A broader phylogenetic analysis of the 91 virus sequences identified during this study highlighted how they commonly clustered together, either with sequences identified during this study or with previously published sequences identified from nematodes^23,31^. In addition, we observed no obvious association between clustering patterns of the virus and the order of its host nematode (Supplementary Fig. 2). For instance, within the *Totiviridae*, we observe virus sequences from four different orders of nematodes appearing on the same branch (Supplementary Fig. 2). The parasites in question often have very different life cycles and transmission mechanisms (vector borne, faecal–oral route) as well as hosts, with minimal likelihood of coinfections (Extended Data Table 1). Taken together, this is indicative of a longer-term association between these viruses and nematodes, and strengthens the evidence for these being authentic viruses of the parasites.

### Identification of viruses in *B. malayi* and *O. volvulus*

To further characterize these viruses, we focused on those within *B. malayi* and *O. volvulus*. After assembling transcriptome sequences, we identified seven RNA virus genome sequences, one in *B. malayi* and six in *O. volvulus*, with four sequences in the latter case identified as partial (Extended Data Table 1 and Supplementary Table 1). Virus identification in *O. volvulus* was further confirmed using viral metagenomics experiments on adult worms collected from Cameroon and Ghana (Supplementary Figs. 2 and 3).

**Fig. 1:**
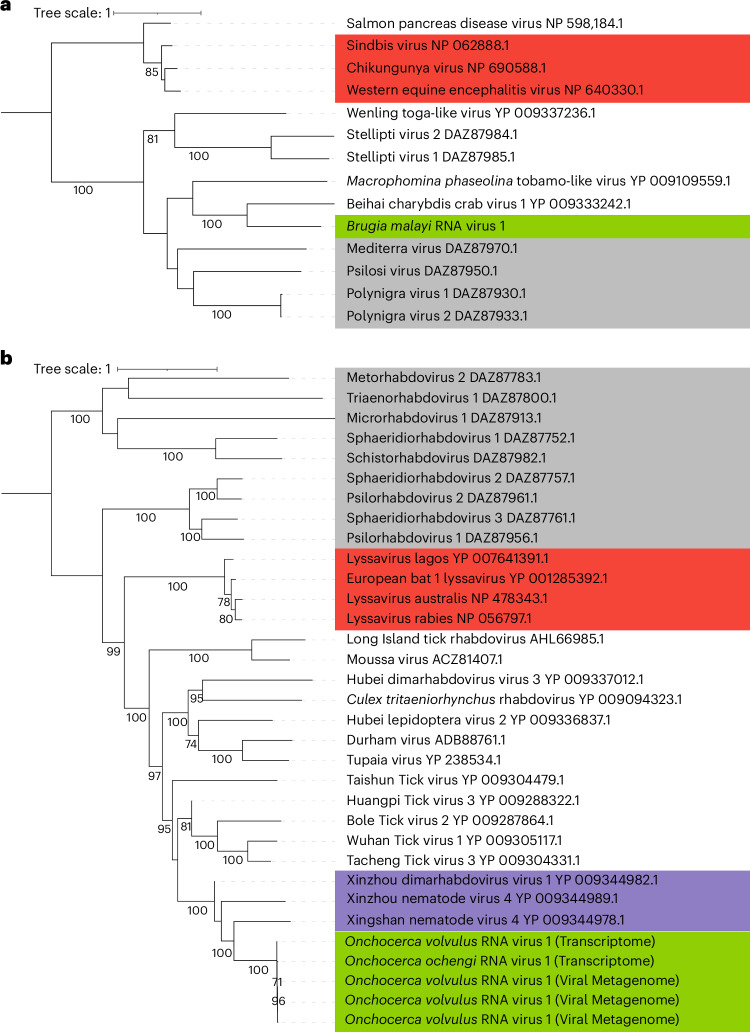
Phylogenetic analysis of BMRV1 and OVRV1 based on alignment of their respective RdRP sequences. **a**, BMRV1. **b**, OVRV1 or the variant found in *O. ochengi* (Onchocerca ochengi RNA Virus 1). Green highlight, viruses found in this study; red highlight, human pathogenic viruses; grey highlight, viruses previously found in flatworms^17^; purple highlight, viruses previously found in other nematodes. Trees were constructed with 1,000 non-parametric bootstrap replicates, and only values above 70% are shown in the figures.

**Fig. 2:**
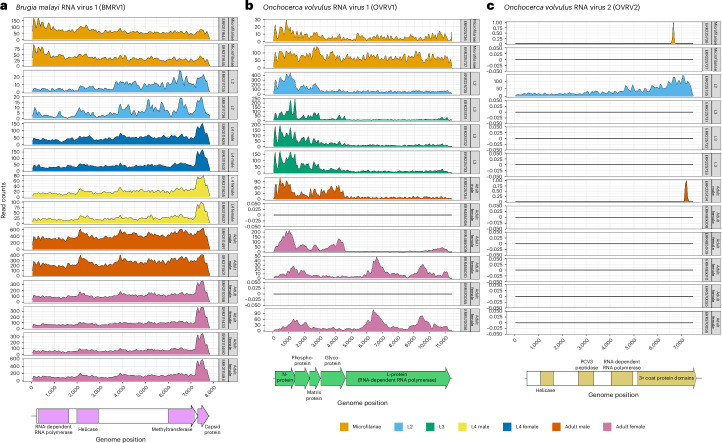
Transcriptome read alignment depths. **a**, BMRV1. **b**, OVRV1. **c**, OVRV2. For each graph, the *x*-axis represents positions in the respective virus genomes, and the *y*-axis represents the number of reads from the transcriptome dataset for different life cycle stages (defined by the grey columns on the right side of each graph and the colour code on the bottom right of the figure), which aligned to the virus genome. An annotation of the virus genomes is at the bottom of each graph, with identified domains and proteins labelled. Source data

Brugia malayi RNA Virus 1 (BMRV1) is a member of the *Togaviridae* family (Fig. 1a) and its sole *Alphavirus* genus, which are positive, single-stranded RNA (ssRNA) viruses. Phylogenetic placement of the RdRP domain clusters BMRV1 with a virus in *Charybdis* crabs^23^, within a larger clade containing human-pathogenic alphaviruses such as chikungunya, western equine encephalitis and Sindbis viruses (Fig. 1a). The genome of BMRV1 is 7.8 kbp, encoding a large polyprotein, including an RdRP, methyltransferase and helicase domains, and a smaller capsid protein (Fig. 2a).

Two of the full-length virus sequences in *O. volvulus* (Onchocerca volvulus RNA Viruses 1 and 2, OVRV1/OVRV2) are predicted members of the *Rhabdoviridae* (negative ssRNA) and *Dicistroviridae* (positive ssRNA). Phylogenetic analysis of the RdRP domains places OVRV1 in a clade containing three other viruses of nematodes^23^ (Fig. 1b). Interestingly, OVRV1 is part of a larger clade that includes the *Lyssavirus* genus, which contains the rabies virus. OVRV1 could be identified in both the published transcriptome^32^ and the viral metagenomics, while OVRV2 could be identified only from the former. The four strains of OVRV1 had a minimum of 96% whole-genome nucleotide sequence similarity to one another (Supplementary Fig. 4). The genome of OVRV1 is 11.4 kbp and predicted to code for five proteins (Fig. 2b): the L protein (RdRP), glycoprotein, nucleoprotein, phosphoprotein and matrix protein. The genome of OVRV2 is 7.4 kbp, encoding one large polyprotein with six identifiable subdomains: helicase, peptidase, RdRP and three capsid subunits (Fig. 2c).

BMRV1 is present in all life cycle stages (Fig. 2a). Adults have the highest number of reads aligning with the viral genome relative to other life cycle stages. OVRV1 is present in all life cycle stages of the nematode, but two out of five female samples showed no aligned reads (Fig. 2b). The presence or absence of reads aligning to the virus genome does not appear to correlate with the total number of sequenced reads^32^. Analysis of viral metagenomic samples from Ghana and Cameroon showed a high variability in read coverage across the OVRV1 genome (Supplementary Fig. 3). Together, this indicates that OVRV1 may not be ubiquitous in all parasites. Interestingly, the expression profile appears to change over time, with more reads aligning to the RdRP or to the structural proteins depending on the parasite life cycle stage (Fig. 2b). By contrast, OVRV2 shows expression in only the L2 stages of the nematode, found only within the black fly vector (Fig. 2c). This may be either a rarely occurring virus in *O. volvulus* or a contamination from the black fly vector.

### BMRV1 prevalence across life cycle stages

**Fig. 3:**
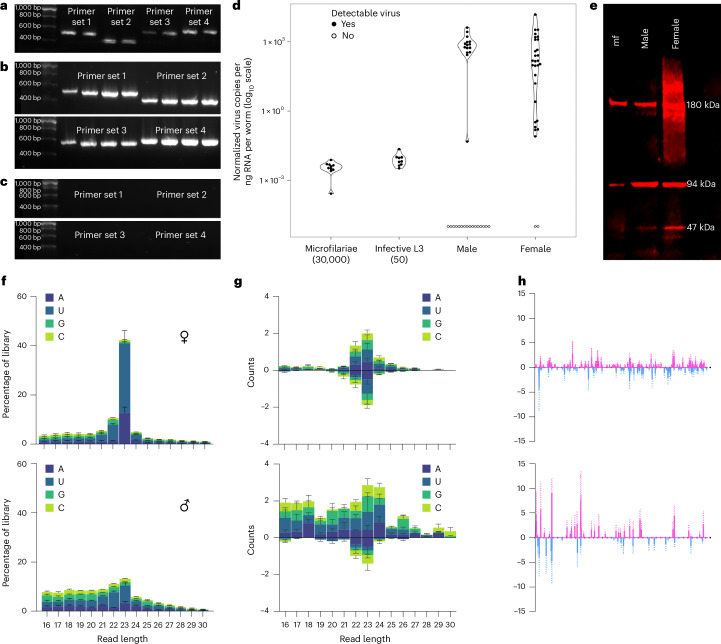
Molecular and sequencing evidence for BMRV1, including RT-PCR, qPCR, western blot and small RNA sequencing results. **a**–**c**, Reverse-transcription PCR experiments show that BMRV1 can be found in LSTM-reared (**a**) and FR3-reared (**b**) *B*. *malayi* and that BMRV1 can be amplified only from reverse-transcribed RNA and not DNA (**c**). More details on the primers used and their target in the BMRV1 genome are given in Supplementary Table 5. The individual lanes shown correspond to different biological replicates of 50,000 pooled *B. malayi* microfilariae (for tests on RNA and cDNA, *n* = 6 total; for gDNA, *n* = 4). **d**, The qPCR results reflect the differences in viral abundance across the life cycle. Note the difference in the number of adult males and females that appear positive for the virus (48% (15 out of 39) and 93% (28 out of 30) of surveyed individuals, respectively). All data points are shown in the graph (pools of mf, *n* = 10; pools of L3, *n* = 9; adult male, *n* = 39; adult female, *n* = 30). **e**, Western blots with antibodies raised against the BMRV1 capsid protein show multimerization and different signal intensities between microfilariae, males and females (*n* = 3 per life cycle stage). **f**, Histogram of total small RNA sequencing of six pools of adult male and female *B. malayi* (*n* = 3 per gender) showing an active siRNA machinery with a length bias at 23 nt that is most visible in samples derived from female nematodes. The histogram shows the mean values of three independent biological replicates (pools) of each gender (*n* = 3) ± s.d. **g**, The size distribution of small RNAs mapping to the BMRV1 genome shows no discernible strand bias, with 23 nt vsiRNAs predominant (especially in females), indicating active processing of BMRV1-derived dsRNA by a Dicer protein. The histogram shows the mean values of three independent biological replicates (pools) of each gender (*n* = 3) ± s.d. **h**, The 23 nt vsiRNA mapping against the BMRV1 genome (pink) and antigenome (blue) (*x*-axis) shows roughly equal coverage against both viral RNA strands, indicating active viral replication. The graph shows the mean values of three independent biological replicates (pools) of each gender (*n* = 3), with error bars indicating ±s.e.m. Source data

Using extracts of *B. malayi* microfilariae, we performed reverse transcription PCR (RT-PCR) on RNA and PCR on genomic DNA (gDNA) targeting four regions of the virus genome, including sections of the polyprotein and the junction between polyprotein and capsid proteins (Fig. 3a,b). All regions amplified to expected band sizes when using cDNA as a template, but not gDNA (Fig. 3c), indicating that RNA virus sequences are not integrated into the nematode genome.

RT quantitative PCR (RT-qPCR) of *B. malayi* RNA extracts from different life cycle stages was performed. After normalization to input quantities of RNA and per individual nematode used for the experiment, we observed that all larvae have low copy numbers of viral genomes (Fig. 3d). This changes dramatically in adults, with both males and females showing high virus levels (1.65 × 10^5^-fold increase in males, 1.83 × 10^5^-fold increase in females, based on weighted means of infection-positive samples from each life cycle stage). However, this viral level is not consistent across all individuals, with 48% of males and 93% of females positive for BMRV1.

To further characterize this virus–host interaction, we analysed small RNA profiles in male and female nematodes, as RNAi responses are critical to fight virus infection in many invertebrate organisms. A previous study has shown an intact small interfering RNA (siRNA) pathway with 5′ triphosphate small RNAs present in *B. malayi* with 22G-5′ triphosphate small RNAs targeting transposons^33^. Previous studies on small RNA responses to virus infection in *Caenorhabditis* nematodes showed that small RNAs are the antiviral defence of these nematodes^34–37^. Infection of *Caenorhabditis elegans* with Orsay virus and small RNA sequencing showed that virus-derived viral short interfering RNAs (vsiRNAs) were predominantly 22 or 23 nucleotides (nt) in length, with a G bias in vsiRNA mapping to the antigenome^34,38,39^. Here we observed vsiRNAs, predominantly 23 nt in length, mapped in equal abundance to the BMRV1 genome and antigenome (Fig. 3f–h) with limited evidence of antisense 22 nt G biased vsiRNAs, also indicating limited production of secondary siRNAs via the *Brugia* RdRP, as seen in *C. elegans*^38^. These data indicate that BMRV1 actively replicates in *B. malayi* and BMRV1 double-stranded RNA (dsRNA) generated during viral replication is targeted by RNAi.

We generated rabbit polyclonal antibodies to recombinant BMRV1 capsid protein (23 kDa) for western blots of *B. malayi* protein extracts from microfilariae and adults. This confirmed the RT-PCR, showing that microfilariae had very low levels of virus capsid proteins. In males and females, we observed a much more abundant signal, reflecting the results of RT-qPCR and transcriptomics. We observed the presence of multimers with three bands, the smallest band of 47 kDa, twice the predicted weight of the BMRV1 capsid protein, with the other two bands indicating tetramers and octamers (Fig. 3e). Using these antibodies, we identified the presence of viral capsid proteins in the culture medium supernatant of female and male nematodes after overnight incubation, suggesting the active release of viral particles (Supplementary Fig. 5).

### Fluorescence in situ hybridization localization of BMRV1

To determine virus tissue tropism, we performed fluorescence in situ hybridization (FISH) with virus-specific probes, *Wolbachia*-specific probes (technical control) and DAPI staining the host nuclei.

**Fig. 4:**
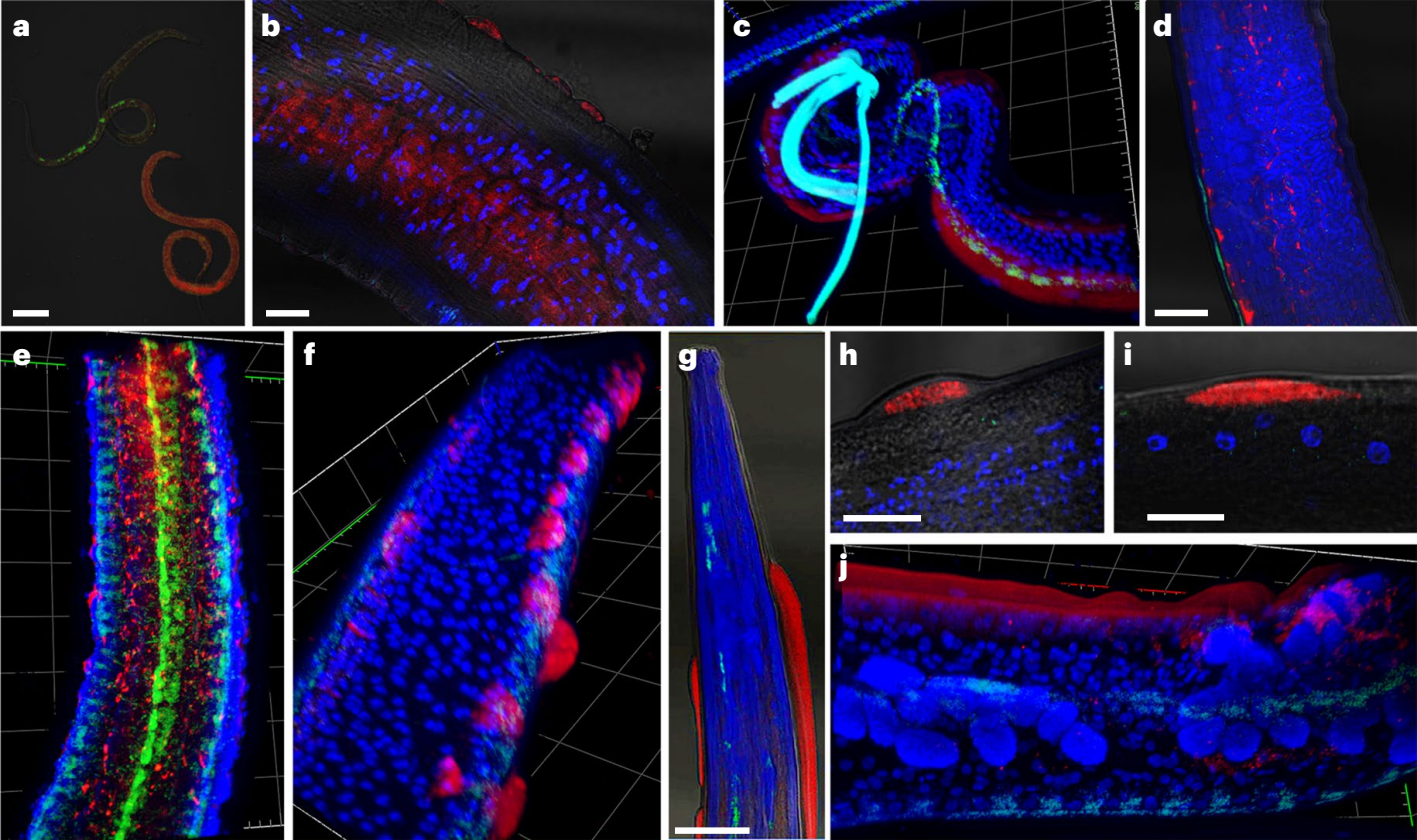
Representative FISH microscopy images of *B. malayi* showing localization of virus RNA within nematode tissues, alongside the *Wolbachia* endosymbiont as a technical control. Virus RNA stained red; *Wolbachia* stained green; DAPI nuclear stain blue. **a**–**e**, Note the different levels of viral infection in microfilariae (**a**), localization of the viral stain in male testes (**b**) and the hypodermal cells near the male spicule (**c**). Virus signal within adult female reproductive tracts appears between developing eggs within the paired uteri of adult females, with early embrys in the left uteri and ‘pretzel-stage’ microfilariae in the right (**d**), with the developing eggs casting a ‘shadow’ in between virus staining, visible in 3D images of female uteri (**e**). **f**–**j**, In older adults (>12 months), we observed ‘epicuticular inflations’ often with an intense viral signal (**f**), typically occurring near the head (**g**) or tail regions of the nematodes. They can appear as single separate inflations at different nematode orientations, either next to internal organs (**h**) or the hypodermal chords (**i**), or as a continuous inflation along the nematode flank (**j**). Scale bars measure 20 µm (**a**,**b**,**h**,**i**) or 50 µm (**d**,**g**). Gridlines in three-dimensional *z*-stack figures (**c**,**e**,**f**,**j**) measure 40 µm by 40 µm. A total of 15 adult male and female parasites were processed in separate experiments. Parasites with epicuticular inflations were typically between 12 and 19 months at the time of sampling, with jird animal hosts being 15–22 months of age, respectively. Parasites without were typically 3–6 months of age, with the jird animal hosts being 6–9 months of age.

Most microfilariae show a weak signal for virus RNA. There are very rare instances of microfilariae that are heavily infected (Fig. 4a, less than 1 per 100 individuals). Adult males and females frequently showed intense signal in patches appearing just beneath the nematode cuticle, the epithelial lining of the reproductive tract and the reproductive tract itself (Fig. 4b–e). Viral signal only rarely appears within the developing embryos or sperm.

In older nematodes of both sexes (>12 months of age), the virus signal appears in patches within ‘epicuticular inflations’ on the nematode cuticle (Fig. 4f–j). These occur most frequently near the head and tail regions of the nematode and adjacent to the male spicule and range between 20 µm and 40 µm in length.

### OVRV1 occurs in both *O. volvulus* and *O. ochengi*

Transcriptomics analysis identified six viruses in *O. volvulus*, of which four were only detected following removal of host transcripts. One *Rhabdovirus* (OVRV1) appeared in all life cycle stages, with the same virus also found in the closely related nematode, *O. ochengi* (~96% overall genome sequence similarity; Supplementary Fig. 4). *O. ochengi* infects cattle and is the most closely related *Onchocerca* sp. to *O. volvulus*^30^.

**Fig. 5:**
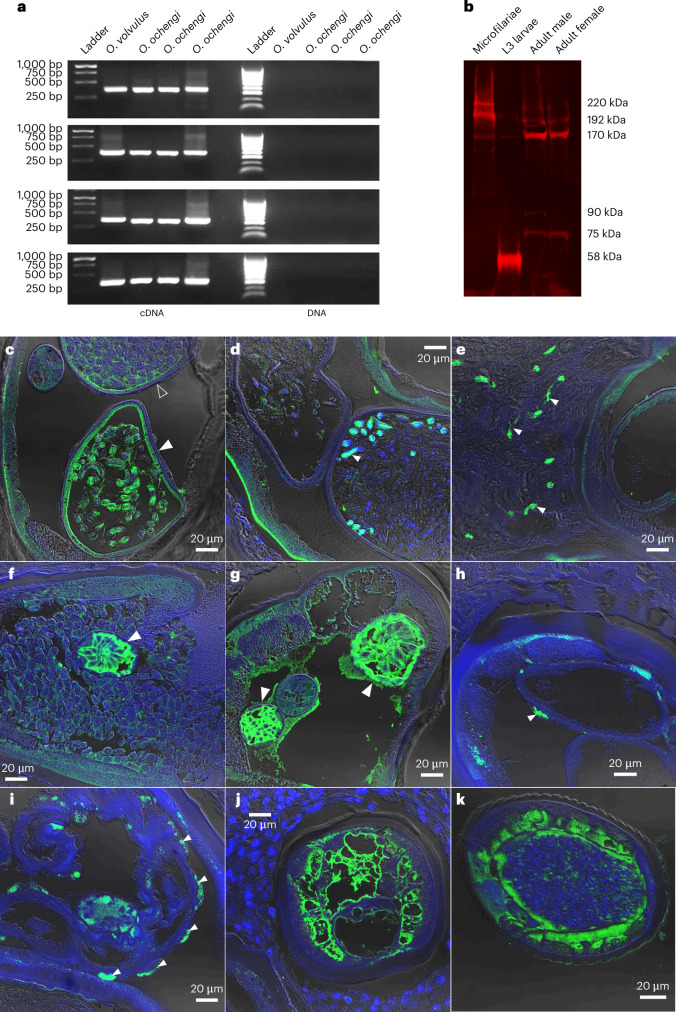
Validation of OVRV1 using RT-PCR, western blot and representative IFA staining of *O. volvulus* nodules with anti-OVRV1 glycoprotein antibodies. Anti-OVRV1 glycoprotein antibodies stained green; DAPI nuclear stain blue. **a**, RT-PCR experiments show that OVRV1 can be amplified only from reverse-transcribed RNA, from both *O. volvulus* (lane 1, *n* = 1) and *O. ochengi* (lanes 2–4, *n* = 3). **b**, Western blots against the OVRV1 glycoprotein show different molecular weight bands occurring depending on the life cycle stage of *O. volvulus* (*n* = 3). All IFA images include the DAPI nuclear stain (blue). **c**,**d**, Images of the paired uteri from adult *O. volvulus* females show virus stains surrounding and entering developing embryos within the uteri (solid arrow), while surrounding but not within the early embryos (hollow arrow). Developing embryos can show either complete infection rates (**c**) or a much smaller proportion (**d**). **e**, Mature microfilariae released from the female, located within surrounding nodule tissues, stain heavily for OVRV1 glycoprotein. **f**,**g**, Intense antibody staining is observed surrounding the nematode rachis, where eggs are first formed (solid arrows). The heavily stained rachis is either surrounded by early-stage eggs with green staining surrounding them (**f**) or without surrounding eggs (**g**). **h**,**i**, Cellular inflations containing intense antibody staining are observed on the external face of the adult female uterine walls (solid arrows). **j**,**k**, Male *O. volvulus* are frequently observed to be infected, with viral stains occurring in different tissues (**j**), as well as surrounding and entering the male testes (**k**). Parasites were obtained from sections of fixed *O. volvulus* nodules from human patients (*n* = 8 nodules). Source data

To validate transcriptome results, we performed RT-PCR and western blots on *O. volvulus* and *O. ochengi* for OVRV1. We designed RT-PCR primers targeting four regions of the OVRV1 genome. We observed amplification for all four targets from *O. ochengi*^40^ and *O. volvulus*^41^ (Fig. 5a). All samples showed amplification at expected sizes, with sequencing showing >96% sequence similarity to their target regions in the assembled OVRV1 genome. We also observed amplification of expected band sizes when testing the same primers against four cDNA libraries made from *O. volvulus* material collected from Guatemala, Cameroon (Kumba and Touboro regions) and Mali^42^ (Supplementary Fig. 6).

The results of RT-PCR were replicated when using western blots on nematode extracts from different life cycle stages, using rabbit polyclonal antibodies raised against the recombinant OVRV1 glycoprotein (Fig. 5b). Interestingly, we observed three distinct western blot profiles that differ based on the life cycle stage of *O. volvulus*. During the development of the infective third-stage larvae (L3), we observed three bands, with the most intense band in line with the glycoprotein’s estimated weight (58 kDa). In adults, we see up to five bands of different weights, with microfilariae showing three out of these five bands (Fig. 5b). This may indicate multimerization and/or post-translational modifications.

### Immunofluorescence localization of OVRV1

To identify the localization of OVRV1 within nematode tissues, we performed immunofluorescence histochemistry with the rabbit polyclonal antibodies raised against the OVRV1 glycoprotein. We assayed eight different *O. volvulus* nodules^41^, all of which were observed to contain fluorescent signal in at least one adult nematode (Fig. 5c–k). Like BMRV1, fluorescent staining of the nematode cuticle was observed, but the most commonly stained tissue was the epithelial lining of the female reproductive tract; fluorescent staining was occasionally observed within the reproductive tract itself, with a strong signal in the rachis (Fig. 5c–g). Like the epicuticular inflations containing BMRV1, we observed multiple inflations of the external face of the female ovary and uterine epithelium (Fig. 5h,i). We further observed developing microfilariae within the reproductive tract, as well as released microfilariae within nodule tissue that showed intense staining for OVRV1 glycoprotein. All male nematodes observed (*n* = 6) stained positive for OVRV1 glycoprotein (Fig. 5j,k).

### Host vertebrate serology to BMRV1 and OVRV1

To investigate potential vertebrate immune responses against nematode viruses, we generated recombinant proteins for the BMRV1 capsid and OVRV1 glycoproteins. We assayed responses to BMRV1 from Mongolian jirds (*Meriones unguiculatus*, henceforth ‘jirds’) used for parasite life cycle maintenance. Responses against OVRV1 were tested using serum from humans infected with or exposed to *O. volvulus*, sourced from the Filariasis Research Reagent Resource Center (FR3) and includes the Centers for Disease Control and Prevention (CDC) and Edna McConnell Clark Foundation^43^ (EMCF) serum banks, together with UK uninfected controls. Serum from jirds infected with *O. ochengi*, which also host OVRV1, was also screened for anti-OVRV1 antibodies.

**Fig. 6:**
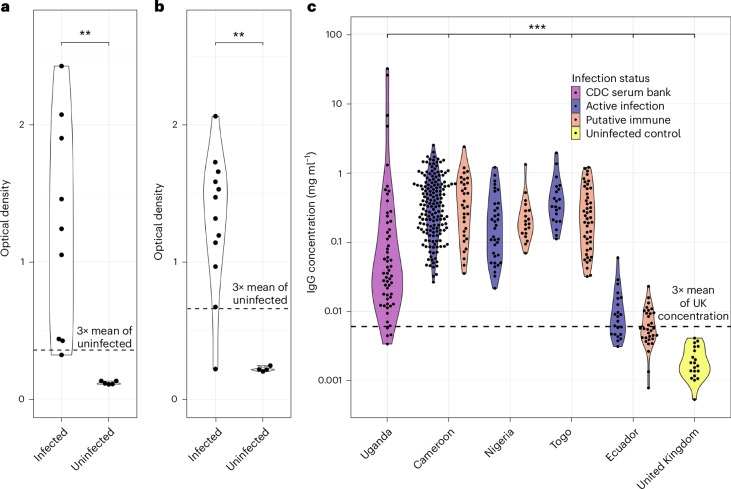
ELISA distribution graphs to illustrate host antibody responses against BMRV1 and OVRV1. Each data point represents the results from one infected individual. **a**, Statistically significant difference in the antibody serology of jirds infected with *B. malayi* against BMRV1 capsid protein when compared with uninfected jirds (*P* = 0.0027). **b**, Statistically significant difference in the antibody serology of jirds infected with *O. ochengi* against OVRV1 glycoprotein when compared with uninfected jirds (*P* = 0.0053). **c**, Serology of *O. volvulus*-infected individuals (‘active infection’ or from the CDC serum bank, dark blue or lavender violin respectively) and individuals without detectable active infection, but resident in the community for at least 20 years (‘putatively immune’, orange violin) against OVRV1 glycoprotein. This shows that all individuals in Africa infected or exposed to *O. volvulus* transmission are seropositive, with 4 samples from Uganda and an additional 26 samples from Ecuador appearing seronegative, when compared with 3× the mean of UK uninfected controls (the dotted line is 3× the mean antibody concentration of UK negative controls). Serum was taken from individuals from Uganda (*n* = 58, *P* = 7.88 × 10^−11^) Cameroon (*n* = 200, *P* = 6.56 × 10^−13^), Nigeria (*n* = 54, *P* = 8.58 × 10^−11^), Togo (*n* = 67, *P* = 3.09 × 10^−11^), Ecuador (*n* = 54, *P* = 5.97 × 10^−9^) and uninfected UK controls (‘uninfected control’, yellow violin). Significance is defined by number of asterisks (**P < 0.01, ***P < 0.001). Significance for all panels was determined via a two-sided Wilcoxon rank-sum test with continuity correction, after identifying the distribution of points as non-normal via a Shapiro–Wilk normality test. Panel **c** uses Benjamini–Hochberg’s *P*-adjustment method for multiple testing correction. ELISA optical density results from human samples were converted into a concentration of IgG antibodies against the OVRV1 glycoprotein using a standard curve of human total IgG.

Western blots of jird serum showed that seven out of nine jirds infected with *B. malayi* were seropositive for antibodies against BMRV1 capsid protein (Supplementary Fig. 7), while enzyme-linked immunosorbent assay (ELISA) showed that eight of these jirds were seropositive compared with uninfected controls (Fig. 6a; seropositivity was determined via cutoff of 3× the mean results of uninfected controls). As OVRV1 can be found within both *O. ochengi* and *O. volvulus*, we performed ELISA experiments on serum from jirds infected with *O. ochengi* parasites^40^, as well as from humans infected with *O. volvulus*^43^. Out of 12 jirds, 11 were seropositive compared with uninfected controls (Fig. 6b). When investigating human serum samples, we found that all *O. volvulus*-infected or exposed individuals across sub-Saharan Africa from Cameroon, Nigeria and Togo were seropositive, as was Uganda, apart from four individuals (Fig. 6c), and all were significantly different from UK uninfected controls. In Ecuador, only 48% of individuals were seropositive for OVRV1 glycoprotein, which was also significantly different from UK controls (Fig. 6c).

## Discussion

We reveal an abundant and diverse RNA virome spanning 14 different viral orders and 24 families within parasitic nematodes. Of the 91 viruses discovered, only 5 have been previously reported, including 3 from *A. suum* and *A. lumbricoides*^23,25^. Our survey is probably an under-representation of the true extent and diversity of the parasitic nematode RNA virome owing to a variety of factors including variations in sample preparation resulting in discarded viral reads and the restricted number, or lack, of transcriptomes for several important parasites. Nevertheless, our analysis supports a conserved global spread of virus–nematode associations across multiple continents in the case of the viruses of *A. suum* and *A. lumbricoides*, and *O. volvulus*, suggesting an ancient and stable co-evolution. This is perhaps best exemplified by members of the Trichinellidae (Supplementary Fig. 1), which show a close evolutionary relationship, as well as phylogenetic clustering of diverse virus sequences from different species and orders of parasitic nematodes.

The parasitic nematodes identified with viruses include several important human parasitic nematodes, *A. lumbricoides*, *T. trichiura*, *O. volvulus*, *B. malayi*, *A. ceylanicum* and *Trichinella spiralis*, which cause substantial public health issues, with over 1.5 billion people infected with one or more such parasites^4–6,44,45^. Several other species cause an even greater global burden in the livestock industry^46^, with 15 economically important parasites (*A. suum*, *Dictyocaulus viviparous*, *Haemonchus contortus*, *Ostertagia ostertagi*, *Oesophagostomum dentatum*, *Teladorsagia circumcinta*, *Trichuris suis* plus 8 *Trichinella* spp.) of cattle, sheep and pigs, harbouring 37 previously unreported viruses.

The full extent and diversity of the parasitic nematode RNA virome, how it impacts nematode biology and whether they act as drivers or modulators of disease pathogenesis remain critical knowledge gaps. Indeed, in the parasite *Toxocara canis*, which causes neurotoxocariasis, components of the TCLA virus have been reported to be highly expressed in infective larvae (18% of expressed sequence tags) before entry into a vertebrate host (for example, humans and dogs)^29^, with human infections eliciting antibody responses against several TCLA virus proteins^29^, indicating potential roles in transmission and infectivity. Alternatively, extrapolation from the most well-characterized RNA viruses of *Leishmania* sp. protozoan parasites suggests potential roles of nematode viruses in disease pathology and progression. Both Leishmania virus 1 (LRV1) and T. vaginalis virus induce hyperinflammatory immunity, which drives disease pathogenesis and subverts host immunity to the parasites’ advantage^14,15,47^. We show that BMRV1 and OVRV1 elicit antibody responses from the host showing direct exposure to the immune system, and we speculate that this suggests the potential to modulate host immunity to the parasite and cross-reactive immunity to other RNA viruses.

We further characterized BMRV1 and OVRV1, infecting *B. malayi* and *O. volvulus*. BMRV1 and OVRV1 cluster within distinct phylogenetic branches containing several human pathogenic alphaviruses (for example, chikungunya virus) and neurotropic lyssaviruses (for example, rabies virus), respectively. This is of relevance for OVRV1, which we propose could potentially play a role in the pathogenesis of OAE. Although BMRV1 and OVRV1 fall within clades containing human pathogenic viruses, these are distant relationships. Here we validate the presence of OVRV1 using transcriptome data (Fig. 2b), metagenomic data and RT-PCR of *O. volvulus*^42^ (Supplementary Figs. 3 and 6). Furthermore, we provide serological evidence of virus exposure in infected and exposed individuals from Uganda, Togo, Nigeria and Cameroon (Fig. 6c). Individuals from Ecuador show a lower proportion of seropositive individuals (48%), which are still significantly different from uninfected controls. OVRV1 also infects the closely related cattle parasite *O. ochengi*, and experimentally infected jirds elicit antibody responses against OVRV1.

*B. malayi* show low levels of BMRV1 in larval stages, which expand dramatically (>1.6 × 10^5^-fold) in adult parasites with a 48% positive rate in males and 93% in females. This suggests variability in the transmission of the virus, changes in host factors that impact replication or the potential for adult worms to mount a protective antiviral immune response. Indeed, our analysis of the siRNA response in *B. malayi* suggests dsRNA intermediates of the BMRV1 genome are targeted by an active host RNAi response with a small-RNA length bias of 23 nt, indicating the presence of an active and functional exogenous siRNA response. Size distribution of reads deriving from BMRV1 in infected samples shows no discernible first position or 5′ bias, indicating cleavage of BMRV1 genomic RNA by processive Dicer patterns. Aligned small RNAs derived from the BMRV1 genome are roughly equal in abundance against both the forward and reverse strands of the positive-sense virus genome, indicating active viral replication and derived dsRNA that is targeted by Dicer (Fig. 3). Tissue tropism in both filarial species shows virus signals in a range of diverse tissues, with a particular abundance in reproductive organs. This might indicate a sexual or vertical transmission route, as well as the potential for virus exposure and transmission into the host during the release of microfilariae. One particularly striking feature was the presence of BMRV1 in epicuticular inflations in older parasites (>12 months), which protrude through cuticular striations clustered around the head and tail. These structures have previously been described in several parasitic nematodes as ‘bosses’, ‘cuticular inflations’ or ‘tubercules’, but without any known function^48–53^. The superficial and exposed surface location of these structures may make them vulnerable to damage, precipitating the release of virus into the host.

The nature of the association of these viruses with their nematode host may include pathogenicity, latency or even mutualism, which are all represented in the diverse RNA virome of invertebrates^23^. Their capacity or likelihood to cross species barriers and infect the parasite’s mammalian host may be linked to whether they adopt ‘generalist’ or ‘specialist’ evolutionary phenotypes, as suggested for arboviruses and some tick viruses^54–58^. Intriguingly, the phylogenetic analysis of platyhelminth viruses indicates that rabies and other vertebrate-associated rhabdoviruses emerged from parasitic flatworm viruses^17^. What we do know is that the parasites that host these viruses, together with those previously reported from helminth flatworms^17^ and protozoan parasites^11^, affect billions of people and animals of economic importance. All of these organisms have intimate, chronic and lifelong exposure to vertebrate host tissues, which may influence disease pathogenesis and nematode biology, and so expands our understanding of parasite diseases.

## Methods

### Ethics statement

All research performed in this study conforms to relevant ethical regulations. All animal experiments using jirds were approved by the ethical committees of the University of Liverpool and Liverpool School of Tropical Medicine (LSTM) and conducted under Home Office Animals (Scientific Procedures) Act 1986 (UK) requirements (license numbers P86866FD9 and PP6173839). Animals had free access to food and water throughout the duration of the studies, were checked daily for welfare and were weighed weekly.

Human serum was obtained from patients enrolled in a double-blind placebo-controlled randomized clinical trial conducted in Cameroon and was approved by the ethics committees of the Tropical Medicine Research Station, Kumba, Cameroon; the Research Ethics Committee of the LSTM, Liverpool, UK; and National Health Service (NHS) National Research Ethics Service (09/H1001/81, Northwest 4 REC). Written informed consent was obtained from all participants, with the exception of those who were illiterate, for which a literate witness signed on behalf of the participant and the participant added a thumbprint (trials registry, number ISRCTN48118452)^41^. Human sera from Uganda, Cameroon, Togo, Nigeria and Ecuador were obtained from the National Institutes of Health (NIH), National Institute of Allergy and Infectious Diseases (NIAID) FR3 (http://www.filariasiscenter.org/), which provides blanket approval for the research community to use as part of a NIAID-funded sample repository. These samples were curated by the EMCF in 1985^43^ and the CDC onchocerciasis serum bank. Ethical approval for use of UK uninfected control sera was obtained from the NHS Research Ethics Committee (16/NW/0170) and the Central Liverpool Research Ethics Committee (protocol number UoL001207). All donors had given approval and consent to be included as part of additional experiments (such as those described in this paper) upon sample collection.

Ethical clearance for the collection of *O. volvulus* parasite material from Cameroon was obtained from S. M. Ghogomu of the Ethics Review and Consultancy Committee, Cameroon Bioethics Initiative, P.O. Box 31489, Biyem-Assi, Yaoundé, Cameroon, reference number CBI/ 443/ ERCC/CAMBIN. Ethical clearance for the collection of *O. volvulus* parasite material from Ghana was obtained via a full board review from the Kintampo Health Research Centre Institutional Ethics Committee, Ghana, study identification number KHRCIEC/2018-18. Written informed consent was obtained from all participants, apart from those who were illiterate, for which a thumbprint was used instead.

### Statistics and reproducibility

Randomization was not relevant for this study, as no particular experimental variable or covariate was being tested. We were interested only in the presence or absence of viruses in datasets or parasite material that is currently available to us. Blinding was also not relevant for this study, as this work was focused on analysing the presence or absence of viruses in material in which no previous knowledge is available. However, as the investigators had no previous knowledge on the parasite’s infection status with viruses (and their jird host’s exposure level to the viruses), data collection and analysis were randomized and performed blind to the conditions of the experiments, except for experiments using serum from jirds and humans whose infection status with their respective parasite was known. No statistical method was used to predetermine sample sizes for experiments performed in this study, as no information on viral infection status is available for sample size calculations. Instead, the investigators used the maximum number of samples possible from previously collected material^40,41,43^. No animals or data points were excluded from the analysis.

### Parasite maintenance

The life cycle of *B. malayi* was maintained in mosquitoes and male jirds as previously described^40^. Briefly, microfilariae (mf) were collected via peritoneal catheterization from jirds infected for more than 12 weeks. The mf were purified using PD10 columns in RPMI 1640 (1% penicillin–streptomycin and 1% amphotericin B) and mixed with heparinized human blood to 15–20,000 mf per ml; the blood with mf was fed to female *Aedes aegypti* mosquitoes from the FR3, using an artificial membrane feeder (Hemotek). After 14 days, infective L3 were collected from infected mosquitoes by crushing and Baermann’s filtration. Jirds were inoculated with 100 L3 larvae via the intraperitoneal route, and adult *B. malayi* were collected from the peritoneal cavity at necropsy, either 6 months or 18 months after infection. Infections with *O. ochengi* were performed by surgical implantation of adult male parasites into jirds, and sera were collected 15 days after infection^40^.

All jirds used to maintain the life cycle at LSTM were purchased from Janvier Laboratories and reared to ethical standards as described previously. Jirds used were all males and aged 3 months when infected with *B. malayi*. Parasites that were maintained in-house can be termed the ‘LSTM strain’, but are descendants of the TRS (now FR3) strain, and were originally obtained from TRS Laboratories. The exact age of the parasites used for experimental processing cannot be monitored. Infected jirds were processed for parasites and/or serum between 18 and 22 months of age. Uninfected, naive jirds were processed between 2 and 3 months of age for serum. The parasites used in this paper were from this ‘LSTM’ strain, unless specifically mentioned (for example, FR3 strain).

### Virus genome assembly and annotation

Transcriptome datasets were downloaded from the European Nucleotide Archive (ENA) through the ENA Browser Tools application^59^ (v1.5.3) via the BioProject accession number, or a list of accessions identified as RNA sequencing from the project details. A full list of this is available in Supplementary Table 1. All datasets chosen for analysis were from experiments originally designed to investigate the parasite transcriptome specifically, either for genome assembly and annotation, or investigation of nematode biology. These datasets used either Illumina sequencing or 454 Titanium pyrosequencing technologies, and assembly. These were trimmed for adapters and quality using bbduk^60^ (v38.79) for a minimum PHRED quality score of 20 and minimum trimmed read length of 50 nucleotides. The reference libraries ‘adapters.fa’, ‘nextera.fa’ and ‘truseq.fa’ from the distributed bbduk package were combined and used for adapter trimming. The resultant reads were then assembled using the program rnaviralSPAdes^61,62^ (v.3.15.3) with default settings, without filtering reads against the host genome. This was done as an initial assembly to conduct a full screen of any virus-like sequences that may be present in the transcriptome or integrated into the host genome. Once assembly was completed, sequences were then analysed using the program Virsorter2 (ref. ^63^; v.2.2.3) using a minimum assembled length of 1,000 base pairs and to screen only against the RNA group of viruses. As additional validation, we also used the program geNomad^64^ (v1.6.1) to screen for viruses. All sequences identified by Virsorter2 and/or geNomad were first validated for quality and completeness using the program CheckV^21^ (v1.0.1). This program looks for potential host contamination in the assembled virus sequences and assigns a predicted ‘completeness’ score to the sequences based on a curated hidden Markov model gene database. Results from CheckV are available in Supplementary Table 2. Validated sequences were subsequently taken for further analysis via BlastX and BlastN against non-redundant protein and nucleotide databases in NCBI, respectively, to identify the closest relatives or presence of potential genome integrations with the nematode host. This was done by limiting the searches to either ‘Viruses (taxID 10239)’ or ‘Nematoda (taxID 6231)’, respectively. Sequences were annotated using PROKKA^65^ (v.1.11) with the standard genetic code to identify reading frames and protein sequences, which were further analysed using InterProScan^66^ to identify functional domains, such as the RdRP. Visualization of virus genomes was done using Artemis^67^ (v16.0.17), and coding regions were plotted using ggplot2 (ref. ^68^; v3.4.2) and the gggenes package^69^ (v0.5.0).

A second reassembly of the reads was also performed after removal of host parasitic nematode transcripts. Genomes for 38 out of the 41 different parasite species were identified from WormBase^70^ (Supplementary Table 1). The same quality-trimmed sequencing reads used in the first pass-through were aligned to their respective parasite species genome (where available) using BWA-mem^71^, run as part of the NVIDIA Clara Parabricks toolkit^72^ (v4.0.1-1) using default parameters. Reads that did not align to the host genome were then removed using Samtools view^73^, with the flags ‘-13’ for paired-end reads and ‘-4’ for single-end read datasets. These remaining reads were then reassembled and analysed using the method described previously.

Based on a combination of results from Blast and InterProScan, all identified viruses were assigned to a putative order, which was used to build sequence alignments for phylogenetics. This analysis was performed using a pre-existing alignment used for virus phylogenetics^23^ downloaded from Figshare supplementary data. The identified RdRP sequences from this study were then added to these alignments based on their putative virus orders. Additional RdRP sequences from viruses of flatworms that were more than 50% complete (less than 50% of identified amino acids were ‘X’) were also added for completeness, with all sequences realigned using MAFFT^74,75^ (v.7.505) using the E-INS-I strategy. Uninformative parts of the sequence were then removed using Trimal^76^ (v1.2rev59), with the gappyout strategy specified. The gappyout strategy allows the removal of ‘gap-rich’ regions (areas of the alignment where there are gaps across many or all sequences used in the alignment) while preserving ‘gap-poor’ regions (areas with few gaps across the sequences). The final alignment was then used for phylogenetic analysis using IQTree^77^ (v.1.6.1) at 1,000 non-parametric bootstraps. ModelFinder^78^ was also used to identify the best evolutionary models (Supplementary Fig. 2 caption). Phylogenetic trees were then visualized and annotated using the Interactive Tree of Life^79^. The full phylogenetic tree can be found in Supplementary Fig. 2, with alignment information and Newick formatted trees found on Figshare (Data Availability statement). In addition to these larger trees built for the different virus families, more ‘focused’ trees were constructed for BMRV1 and OVRV1 specifically. In these cases, branches of interest were identified from the larger overall trees, and the original amino acid sequences downloaded and reprocessed following the above method for the generation of clade-specific phylogenetic trees. These ‘focused’ trees were visualized in Figtree v1.4.4 (ref. ^80^) and annotated using iTOL^79^.

In addition to the phylogenetic trees for the different viruses, non-parametric bootstrap trees for a group of totiviruses, as well as their *Trichinella* hosts, were constructed to investigate their co-evolutionary relationship with their parasite host. To construct the phylogenetic tree of the Trichinellidae parasite hosts, single-copy orthologue genes were identified from the parasite proteome (downloaded from WormBase ParaSite) using the program OrthoFinder^81^ (v2.5.1). Protein sequences from these genes were aligned individually, before the aligned sequences were concatenated together. This was then used to build a phylogenetic tree at 1,000 non-parametric bootstraps, using the evolutionary model JTT + F + I + G4 (as defined by ModelFinder^78^). A phylogenetic tree of the totiviruses was created by aligning the RdRP amino acid sequence, using 1,000 non-parametric bootstraps and the evolutionary model JTT + F + G4. These two trees were then manually compared to construct a tanglegram to evaluate potential virus–parasite co-evolution (Supplementary Fig. 1).

Frequency of virus prevalence within datasets was performed by first concatenating the virus-like sequences to the end of the nematode host genome (where available, nematode genome accessions used are available in Supplementary Table 1), thus acting as a false chromosome. This concatenated genome was used as a ‘reference genome’, allowing competitive alignment and ensuring that sequencing reads from the nematode host did not align to the virus. Alignment was performed using the same quality-trimmed sequencing dataset used as input for assembly (Supplementary Table 1), and BWA-mem^71^, run as part of the NVIDIA Clara Parabricks toolkit^72^ (v4.0.1-1). This is a computational workflow designed to take advantage of graphical processing units to increase the speed of alignment and variant calling exercises compared with standard workflows^82^. Resultant bam files were then processed using the program MosDepth^83^ (v0.3.2) to calculate average read depths in 50-nucleotide ‘bins’ across the virus ‘chromosome’ specifically (options ‘–chrom’ and ‘–by’). The tabular output ‘regions.bed’ file was then used in R, and the mean read depth was calculated across the virus genome. A virus was considered present in a dataset based on an arbitrary mean read depth of 0.5 (Supplementary Table 1) to account for chance matches, as well as the possibility of low viral titres in any one sample.

Additional visualization of read alignments for BMRV1 and OVRV1 identified in *B. malayi* and *O. volvulus* was further performed by graphing the outputs from MosDepth^83^ (v0.3.2) into ggplot2 (ref. ^68^; v3.4.2).

Visualization of read alignments to BMRV1 and OVRV1 identified in *B. malayi* and *O. volvulus*, respectively, was performed with (PRJNA772674 (ref. ^84^) for BMRV1, PRJEB2965 (ref. ^32^) for OVRV1). The virus-like sequences were first concatenated to the end of the nematode host genomes to act as a false chromosome (nematode genome accessions used available in Supplementary Table 1).

### *O. volvulus* sample collection, RNA extraction and sequencing

Nodulectomies were performed on identified nodules of persons with an *O. volvulus* infection from both Ghana and Cameroon. All nodulectomies were performed under aseptic conditions and local anaesthetics by trained medical personnel. Once the nodules were recovered, the human tissue was digested using 0.5 mg ml^−1^ collagenase in RPMI for 9 h at 37 °C, shaking at 90 rpm. All worms were either pooled by gender or sequenced separately (Supplementary Table 3). The worms collected from Cameroon were washed three times and then placed in RNALater to be stored at −80 °C. Worms collected in Ghana were handled in a similar manner but placed in alcohol to be stored at −20 °C to be later transferred to −80 °C in saline for long-term storage and shipment to Belgium.

All samples were processed according to the NetoVIR protocol^85^ to enrich them for viral-like particles. The original protocol was slightly modified by adjusting the initial sample preparation for nematode homogenization with the Precellys homogenizer in three intervals of 30 s at 4,500 rpm with the addition of Precellys 2.8 mm zirconium oxide beads (Bertin Technologies). Paired-end sequencing of samples was performed on a NovaSeq 6,000 system for 300 cycles (2 × 150 bp) at the Nucleomics Core sequencing facility (VIB).

### Confirmation of OVRV1 in adult *O. volvulus* nematodes

We performed additional sequencing of *O. volvulus* parasites collected from Ghana and Cameroon (available under PRJEB67302). This sequencing yielded between 24.40 and 53.28 million sequences per sample (Supplementary Table 3), which were trimmed for quality and adapters with trimmomatic^86^ (v0.39). Good-quality reads were subsequently mapped to the OVRV1 genome specifically with Bowtie2 (ref. ^87^) (v2.4.2) on default settings. Consensus sequences were extracted from the resulting BAM files using samtools^73^ (v1.15), bcftools^88^ (v1.8) and bedtools^89^ (v2.29.2). Finally, the consensus sequences were manually curated in Geneious Prime (v2023.1.2). Visualization of aligned reads was performed as previously described.

### Nucleic acid extraction from *B. malayi* and *Onchocerca* nematodes

For production of RNA, mf, L3, and adult male and female *B. malayi* were collected via the life cycle maintained at LSTM, with additional adults obtained from the FR3. *O. volvulus* and *O. ochengi* adults stored in liquid nitrogen were also used from previous studies^40,41^. *B. malayi* material generated at LSTM was placed directly in TriZol for storage at −20 °C, while adults from FR3 were placed in RNALater before shipping to LSTM. The mf were divided into 10 aliquots of 30,000 (used for RT-qPCR) or 4 lots of 50,000 (used for PCR) and placed in Bertin Instrument tubes (model VK05-2ml) suspended in a total volume of 1 ml of TriZol. Individual adults and batches of L3 (50 per batch) were transferred from their storage tubes into 1 ml of TriZol in Bertin Instrument tubes (model SK38-2ml). Archived *O. volvulus* and *O. ochengi* parasite material were removed from liquid nitrogen, allowed to defrost and then transferred into 1 ml of TriZol in Bertin Instrument tubes (model SK38-2ml). Batches of material were homogenized at 6,000 rpm (Minilys, Bertin Instruments) for 3 × 60 s, cooling on ice for 60 s in between. The material was then left overnight at −80 °C, and the next day subjected to heat-shock at 75 °C for 1 min to lyse any remaining material before being placed on ice. Adult samples were further homogenized for one additional round. The resultant extracts were then removed from the tubes and placed into 2 ml microcentrifuge tubes, which were then spun to pellet any cellular debris. The supernatant was then transferred into a fresh 2 ml microcentrifuge tube, to which an equal volume (~700 µl) of 100% ethanol was added. From here, the Zymo Direct-zol RNA Miniprep kit (catalogue number R2062) was used following the manufacturer’s protocol, but excluding on-column DNAse treatment, with RNA eluted in the final step using 25 µl of supplied DNAse- and RNAse-free water.

We generated additional gDNA for *B. malayi*, *O. volvulus* and *O. ochengi*. gDNA material for *B. malayi* was obtained from 10,000 mf using Qiagen’s QIAamp DNA kit (catalogue number 51306), following the manufacturer’s instructions for isolation of genomic DNA from tissues. gDNA material for *Onchocerca* parasites was also obtained from previously mentioned archived samples^40,41^, using the same DNA extraction kit and method.

Concentration of RNA was then quantified using a Nanodrop spectrophotometer (IMPLEN NanoPhotometer NP80) with two measurements of 1 µl volumes. A third measurement was taken if there was a discrepancy in detected concentration of more than 2 ng µl^−1^. For the RNA extracts, the remaining volume of nucleic acids was then DNAse-treated in-solution using Lucigen’s Baseline-ZERO DNase (catalogue number DB0715K) following the manufacturer’s protocol, scaled-up to the correct volume. Up to 9.5 µl of the resulting RNA was used for the synthesis of cDNA, using Jena Bioscience’s SCRIPT cDNA synthesis kit (catalogue number PCR-511L), using random primers and following the manufacturer’s instructions.

### Small RNA sequencing and analysis

RNA was extracted from pools of BMRV1-positive *B. malayi* female (three pools of three) and male (three pools of eight) adults, and small RNA sequencing was undertaken at Beijing Genomics Institute using the DNBSEQ unique molecular identifier (UMI) small RNA sequencing technology platform. Briefly, small RNA of read lengths 18–30 nt were first selected via polyacrylamide gel electrophoresis (PAGE), and a 5′-adenylated and 3′-blocked adaptor was ligated to the 3′ end. A UMI-labelled primer was then added, followed by the ligation of an adaptor to the 5′ end. First-strand synthesis was performed with the UMI-labelled primer, followed by second-strand synthesis. Fragment selection was carried out to isolate fragments of the desired size. Finally, the double-stranded PCR products were circularized by the splint oligo sequence, forming single-strand circular DNA sequenced in a single-end 50 bp format. The six libraries were sequenced, with final clean tags of all libraries between 25.18 and 25.37 million reads. Basecalled fastq files were quality and adapter trimmed using SOAPnuke^90^ with parameters ‘n 0.001 -l 13 -q 0.1–highA 1–minReadLen 15–maxReadLen 44–ada_trim’. Trimmed reads were then mapped to the BMRV1 genome using Bowtie2 (v2.4.5)^87^, with local mapping flag (–local). The trimming and mapping statistics summary is available in Supplementary Table 4. The histogram of mapped read lengths and first base pair bias was generated using bash scripts available from github.com/rhparry/viral_sRNA_tools/, specifically 3_bam_sRNA_histogram.sh, which uses samtools (v1.16.1). Output BAM files were filtered to include only the 23 nt reads, and coverage for each position of the BMRV1 genome was extracted using bedtools genome coverage tool (v.2.27.1)^89^ and visualized using GraphPad Prism (v10.0.2).

### PCR for visualization of virus presence or absence

Nucleic acid material generated as previously described (both RNA and gDNA) were used for confirming the presence of RNA virus genomes. Four primer sets for BMRV1 were designed using the NCBI PrimerBlast program, screening against *B. malayi* material from FR3 and additional microfilariae material generated at LSTM. The four sets were chosen based on their location in the virus genome, with two sets overlapping the gene junctions between the polyprotein and capsid protein of BMRV1, and the remaining two overlapping the polyprotein’s methyltransferase and RdRP domains. An additional four primer sets for OVRV1 were designed using the same tool, screening against *O. volvulus*. The four sets targeted different regions of the virus genome, with two overlapping with the L-protein of OVRV1, and the remaining two overlapping with the virus’ N-protein and phosphoprotein genes. The analysis was done using cDNA as well as gDNA templates generated as described earlier. PCR was performed using Jena Bioscience’s Taq Core Kit (catalogue number PCR-214S) following the manufacturer’s protocol, with all samples using 10 µl input cDNA and gDNA into 20 µl volume assays. Cycling conditions were as follows: 2 min at 95 °C before being subjected to 35 cycles of 20 s at 95 °C, 20 s at 60 °C and 40 s at 72 °C. The amplicons were run on a 2% Tris–borate EDTA agarose gel with 1× GelRed (Biotium) and visualized using a UV transilluminator (Syngene). Primer sequences are available in Supplementary Table 5.

### qPCR for analysis of virus genome copies per life cycle stage

Primer sequences for qPCR analysis of virus genome copy number are described in Supplementary Table 5, with the primer efficiency of amplification validated to 100% (±10%) using five serial dilutions of cDNA material, replicated on at least two separate experiments. All qPCRs were done using a total volume of 20 μl, with 2 μl of cDNA from each sample, forward and reverse primer concentrations of 0.5 μM and 10 μl of QuantiTect SYBR Green PCR kit master mix (Qiagen). All reactions were run in duplicate and first heated for 2 min at 50 °C, followed by 15 min at 95 °C, before being subjected to 40 cycles of 30 s at 94 °C, 30 s at 60 °C and 30 s at 72 °C. A melt-curve analysis was then performed as the final step, from temperatures 55 °C to 95 °C.

A stock of template cDNA for the primers was then generated by performing PCR on extracted cDNA, using Jena Bioscience’s Taq Core Kit (catalogue number PCR-214S). This was then run on a 2% Tris–borate EDTA agarose gel with 1× GelRed (Biotium) and visualized using a blue light transilluminator. The resultant amplification bands were then cut out, nucleic acids purified using Zymo’s Gel DNA Recovery Kit (catalogue number D4007) and the concentration of eluted cDNA quantified by Nanodrop spectrophotometery (IMPLEN NanoPhotometer NP80). Based on the weight of recovered cDNA and known amplicon size from primer design, copy numbers were then deduced using the following formula:

$${\mathrm{Amplicon}}\;{\mathrm{copy}}\;{\mathrm{number}}=\frac{({\mathrm{Weight}}\;{\mathrm{of}}\;{\mathrm{recovered}}\;{\mathrm{cDNA}}\times 6.0221)\times {10}^{23}}{({\mathrm{Length}}\;{\mathrm{of}}\;{\mathrm{amplicon}}\times 660)\times {10}^{9}}$$

This template was then diluted to a concentration of 10^7^ copies per µl and used as starting material for serial dilutions to create a standard curve for virus genome quantification. All samples were run using the Quantstudio 5 system, following the qPCR protocol as described earlier. The standard curve was used for exact quantification of virus genome copy numbers in each individual sample. This was then first normalized based on the input amount of cDNA used for the reaction, determined by the concentration of extracted RNA. A second normalization step was then performed, by dividing this copy number concentration by the number of nematodes used for the RNA extraction process (that is, 30,000 for mf, 50 for L3, individuals for adult males and females).

### FISH

Probes for FISH were designed using the OligoMiner online application^91^, with modification to the following settings: minimum length of 20 bp, minimum GC content of 40%, all instances of salt (Na^+^) concentrations at 750 mM, linear discriminant analysis temperature model at 37 °C and secondary structure prediction at 37 °C. Probes were designed to target BMRV1 in this manner and had an ATTO-647 dye attached to the 5′ end (IDT). Probes targeting the *Wolbachia* endosymbiont were used as a technical positive control, with sequences taken from previously published literature^92^ and an ATTO-488 dye attached to the 5′ end. An additional probe targeting the Flock House Virus^93^ using an ATTO-647 dye (IDT) was also included to act as a non-binding negative control. All probe sequences used as part of this work are listed in Supplementary Table 5.

Adults or mf of *B. malayi* nematodes were either shipped from FR3 (6-month-old adults) or collected from jirds maintained at LSTM (mf or >12-month-old adults), and stored at −80 °C until fixation and preparation. Nematodes were first fixed in 70% ethanol and 1× PBS and left overnight at 4 °C, with a second fixation step in 4% paraformaldehyde and 1× PBS for 15 min, before two washes in 1× PBS. They were then permeabilized using 10 µg ml^−1^ of pepsin solution for 10 min at 37 °C. For mf, each change of supernatant and buffer was preceded by centrifugation at 3,000 × *g* for 10 min to pellet the mf. Additionally, permeabilization of mf was performed using 5 µg ml^−1^ of pepsin for 30 min. All samples were then incubated at 37 °C overnight with 100 µl hybridization buffer (50% formamide, 5× saline sodium citrate (SSC), 200 g l^−1^ dextran sulfate, 250 mg l^−1^ poly(A), 250 mg l^−1^ salmon sperm DNA, 250 mg l^−1^ tRNA, 0.1 M dithiothreitol (DTT), 0.5× Denhardt’s solution) and 2 µl of each target probe (combination of *Wolbachia*, BMRV1 or none depending on the experiment). After overnight hybridization, the supernatant was removed and the nematodes were washed in 100 µl of hybridization buffer without any probes for 15 min at 37 °C, followed by two washes of 1× SSC buffer with 10 mM DTT, another two washes with 0.1× SSC buffer and 10 mM DTT, and a final wash with 1× PBS. For mf, only one wash with each buffer was performed instead of two. All wash steps were carried out at room temperature using a microcentrifuge tube rotator for 5 min each, removing the supernatant after each wash. After the washes, the nematodes were mounted on slides using Vectashield Mounting Medium with DAPI (Vector Labs). The nematodes were then visualized with a confocal laser-scanning microscope (Zeiss LSM 880, software Zeiss ZenBLUE Edition (v3.7.97.02000)) and images captured with ×40 or ×60 oil objectives.

### Expression of recombinant virus proteins

The full-length BMRV1 capsid protein cDNA was obtained from *B. malayi* adult female cDNA (SAW96MLW-BmAF) in the λ-Uni-ZAP XR vector library^94^ (courtesy of S. Williams, Smith College). This was amplified from the bacteriophage DNA by PCR using forward and reverse primers corresponding to the ends of the open reading frame sequence. All primer sequences used are detailed in Supplementary Table 5.

The C-terminus of the OVRV1 glycoprotein was obtained via PCR of an *O. volvulus* L3 cDNA library constructed in λ-Uni-ZAP XR^94^ (Stratagene; Smith College) using forward and reverse primers as described in Supplementary Table 5. This resulted in 624 bp nucleotides that were expressed as a protein of 24 kDa with a iso-electric point (PI) of 6.58.

The amplified PCR products were subcloned into the TA vector, pCR2.1-TOPO (Invitrogen). The insert from pCR2.1-TOPO was re-amplified and subcloned into the expression vector, pJC40 (ref. ^95^). The constructs were then transformed into *E. coli* DE3 cells, induced with isopropyl-1-thio-d-glucopyranosides (IPTG) and expressed as a fusion with an N-terminal poly-histidine tag. Results were checked by nucleotide sequencing of constructs (Source Bioscience) and protein sequencing (Functional Genomics and Proteomics Laboratories, University of Birmingham) to confirm that the final products were correct. Recombinant proteins were then purified by affinity chromatography on a nickel column (Probond resin, Invitrogen) according to the manufacturer’s instructions.

### Production of polyclonal antibodies

Antibodies to recombinant BMRV1 capsid proteins and to C-terminal OVRV1 glycoprotein were produced in rabbits by immunization with recombinant antigen as described above. In brief, the products were emulsified in Freund’s complete adjuvant (for the first inoculation) and Freund’s incomplete adjuvant (subsequent inoculations). The process was carried out by Alta Bioscience, with 200 µg of protein antigen administered by subcutaneous injection on three occasions, at an interval of 3 weeks. Rabbits were bled 8 days after boosting. No experimental work was performed using rabbits.

### Gel electrophoresis and immunoblotting

Proteins were extracted from parasites by boiling for 10 min in electrophoresis sample buffer (3% (w/v) SDS; 62 mM Tris–HCl, pH 6.8; 15% (v/v) glycerol) containing 5% 2-mercaptoethanol. The lysates were homogenized and vortexed before boiling. Insoluble material was removed by centrifugation for 5 min at 16,000 × *g*. The parasite extracts from different life cycle stages were then fractionated on either 12% NuPAGE Bis–Tris protein gels using morpholinoethanesulphonic acid (MES) buffer or NuPAGE 3% to 8% protein gel using Tris–acetate buffer (Invitrogen). The protein markers used included phosphorylase b (94 kDa), bovine serum albumin (68 kDa), ovalbumin (43 kDa), carbonic anhydrase (30 kDa), soybean trypsin inhibitor (20 kDa) and α-lactalbumin (14 kDa). Separated proteins were electrophoretically transferred to nitrocellulose, and the membranes were blocked by incubation in 5% skim milk in TST buffer (0.01 M Tris, pH 8.5; 0.15 M sodium chloride; and 0.1% Tween) for 1 h at room temperature. Blots were incubated with rabbit antisera to BMRV1 capsid protein or OVRV1 glycoprotein at 1:2,000 dilution in 5% milk overnight at 4 °C. Control blots were probed with preimmune rabbit sera. The detection was performed using IRDye anti-rabbit specific antibodies 1:15,000 in 5% milk for 1 h at room temperature (IRDye 680RD goat anti-rabbit IgG (Li-COR Biosciences)). The results were visualized in 700 nm fluorescence channels by Odyssey FC (LI-COR Biosciences).

To detect jird immune responses against virus surface proteins, we used freshly collected jird blood from the existing life cycle or naive, uninfected jirds (for the detection of BMRV1 capsid protein), or archived jird serum^40^ (for OVRV1 glycoproteins). For BMRV1, naive and *B. malayi*-infected adult gerbils were euthanized by CO_2_ inhalation. Blood was collected from the animal heart by cardiac puncture and centrifuged at 13,000 *g* for 10 min at 4 °C to separate the blood and collect serum (repeated if necessary). Recombinant virus proteins were purified as described earlier and transferred via gel electrophoresis and immunoblotting. After transfer onto a nitrocellulose membrane, each protein lane was cut, labelled and blocked, and then placed into a 5 ml tube for reaction with individual gerbil serum overnight at 4 °C with agitation. The membrane was then washed five times with TST buffer, then incubated with rabbit anti-Mongolian gerbil IgG (H + L), (catalogue number BS-0403R, Bioss) for 1 h at room temperature. The membrane was washed 5 times with TST buffer, then incubated with IRDye anti-rabbit-specific antibodies 1:10,000 in 5% milk for 1 h at room temperature (IRDye 680RD goat anti-rabbit IgG (Li-COR Biosciences, 926-68071). After incubation and washing in TST, the results were visualized in 700 nm fluorescence channels by an Odyssey FC imager (LI-COR Biosciences).

### ELISA for OVRV1 glycoprotein using human sera

Human sera were obtained from the FR3, originally derived from the EMCF^43^ and the CDC^43^. Samples originated from endemic regions within Cameroon (*n* = 200), Togo (*n* = 67), Nigeria (*n* = 54), Uganda (*n* = 88) and Ecuador (*n* = 54). Sampled individuals ranged in age from 3 to 95; the median age per country was 51, 35, 39, 33 and 33, respectively, with male to female ratios of 98:82, 31:27, 42:12 and 21:33, respectively (where these data were given, Uganda had no information on participant gender). In each of these locations, sera were derived from individuals showing parasitological signs of current infection (classified as ‘infected’) or from individuals that had neither palpable nodules nor positive skin snips (classified as ‘putatively immune’). EMCF criteria for defining putative immunity include a minimum of 20 years residency in the endemic area and with no history of recent treatment (past 3 years). Sera from unexposed individuals were obtained from volunteers from the United Kingdom (*n* = 16). This was analysed by ELISA for the presence of antibodies against the OVRV1 glycoprotein. Briefly, Maxisorp plates (Nalge Nunc International) were coated overnight at 4 °C with purified OVRV1 glycoprotein at a concentration of 1 µg ml^−1^ in 0.05 M carbonate buffer (pH 9.6). Wells were blocked by overnight incubation with 20% (v/v) soya milk in TST (Tris–sodium chloride–Tween buffer). Sera were diluted 1:300 in 20% soya/TST and applied to duplicate plates for 2 h at room temperature. The wells were washed in TST, and goat anti-human IgG (H + L) horseradish peroxidase conjugate (Nordic Immunological Laboratories) was added at 1:2,000 dilution in 20% soya/TST for 1 h at room temperature. Plates were washed, and the assay was developed using SIGMAFASTTM OPD (*o*-phenylenediamine dihydrochloride) tablets (Sigma). The absorbance at wavelength 492 nm was read on a Dynastic MR 5000 plate reader and converted to IgG concentrations (ng ml^−1^) using a standard curve generated from ‘IgG (Total) Human Uncoated ELISA Kit with Plates’ (Invitrogen). The standard curve formula and the optical density of all samples per group were calculated and visualized using ggplot2 (ref. ^68^; v3.4.2), ggbeeswarm^96^ (v0.7.2), viridis (v0.6.5)^97^ and ggsignif^98^ (v0.6.4) using R^99^ in R studio^100^. Final IgG concentrations against OVRV1 glycoproteins were normalized using the standard curve formula generated. A total of 30 samples from the Ugandan cohort were not within the standard curve detection range and were excluded from analysis, rather than being replaced with the maximum detected value, to avoid incorrect statistical analysis. We first tested for normal distribution and equal variances via a Shapiro–Wilk test and Levene’s test, respectively. Data were deemed non-normal with unequal variances, so the presence of statistical differences was tested for using a Kruskal–Wallis test, followed by Wilcoxon rank-sum tests with Benjamini–Hochberg multiple testing correction. As we are testing only for differences in distribution in our data, and not differences in mean and median, our data meet the assumptions of the Wilcoxon rank-sum test. All statistical tests were performed using R^99^ in R Studio^100^.

### Immunohistochemistry of onchocercomata

Nodules from *O. volvulus*-infected patients were used from the northwest province of Cameroon collected as part of a double-blind placebo-controlled randomized trial of doxycycline (6 weeks ± ivermectin 4 months after the start of treatment) and placebo^41^. Onchocercomata were surgically removed 21 months after treatment, fixed in 80% ethanol and embedded in paraffin. Onchocercomata from placebo-treated individuals were used for immunohistochemistry (IHC), with 4 µm sections mounted on poly-l-lysine slides and rehydrated through serial dilutions of xylene and ethanol to water. After heat-induced antigen retrieval in 1 mM EDTA, pH 8.0, the sections were encircled with a hydrophobic pen and blocked in 5% normal rabbit serum for 1 h at room temperature. The presence of virus was assessed using rabbit monospecific serum against OVRV1 glycoprotein at a concentration 1:500 in Ultraclean antibody diluent (Thermos Scientific TA125) overnight at 4 °C. After washing sections in Tris–saline buffer, we used a secondary anti-rabbit antibody from goats (AF488, Life Technology), diluted in Ultraclean antibody diluent at a concentration of 1:1,000. Slides were mounted in Vectashield Mounting Medium with DAPI (Vector Labs). Non-specific binding control staining was performed by omitting primary antibodies in one slide per batch. Sections were visualized with a confocal laser scanning microscope (Leica DM2500), and images were captured with ×20 or ×40 oil objectives.

### Western blot of the excretory–secretory products of *B. malayi* microfilariae and adult worms

To determine whether BMRV1 capsid protein was excreted during parasite culture, a western blot with anti-BMRV1 capsid protein was performed on excretory–secretory products collected from the supernatant of freshly collected *B. malayi* adult worms. A total of 29 females and 14 males collected from one jird were incubated in two petri dishes (one dish per nematode gender) containing 10 ml of RPMI with penicillin, streptomycin and amphothericin B (concentrations of 1,000 units ml^−1^, 1,000 µg ml^−1^ and 250 µg ml^−1^, respectively) at 37 °C for 24 h. Adults were then removed and the media centrifuged at 6,000 *g* for 15 min to remove any host material or released mf. The clean supernatant was collected and precipitated using acetone and trichloroacetic acid (TCA). Briefly, a solution in a ratio 1:8:1 was mixed in the following order: 1 ml supernatant, 8 ml 100% ice-cold acetone and 1 ml 100% TCA, stored at −20 °C for 1 h and centrifuged at 18,000 *g* for 15 min at 4 °C. The pellet was washed three times to remove all the TCA. The pellet was air-dried, and the precipitated proteins were then dissolved in SDS–sample buffer for SDS–PAGE and western blot.

### Nematode immunofluorescence

Nematode adults were fixed in 4% PFA (Electron Microscopy Sciences) in PBS at 37 °C for 2 h and were then fixed again in 4% PFA in PBS for 16 h at 4 °C. Worms were washed with PBST (0.1% Triton-X 100 (Sigma-Aldrich) in PBS) and permeabilized overnight in 2-mercaptoethanol solution (5% 2-mercaptoethanol, 1% Triton-X 100, 120 mM Tris, pH 7.0) at 37 °C. Following thorough washes in PBST, the worms were blocked in 2% BSA in PBST for 1 h and then incubated with the primary antibodies at the proper antibody diluent (2% BSA in PBST) overnight. After washing with PBST, the secondary antibodies were used that were conjugated with immunofluorescence antibody (IFA) or Alexa Fluor 488 for 1 h at room temperature. The worms were washed in PBST, mounted using Fluoromount aqueous mounting medium and visualized using a confocal laser scanning microscope (Zeiss LSM 880).

### Reporting summary

Further information on research design is available in the Nature Portfolio Reporting Summary linked to this article.

## Supplementary information

Supplementary Information Supplementary Figs. 1–7 and source data for Supplementary Figs. 5–7.

Reporting Summary

Supplementary Table 1. A full table containing details of viruses identified during this study, the nematode transcriptomes analysed, number of datasets showing an average read depth of >0.5 for the virus, PUBMED ID number of the study that generated the transcriptome and method of library generation as described in the original publication. Supplementary Table 2. Results from CheckV with details on assembly length, total gene count, predicted virus gene count and predicted sequence completion status. Supplementary Table 3. Details of *O. volvulus* parasite sample collection, storage and pooling from Cameroon and Ghana. Supplementary Table 4. Primer and FISH probe sequences used during this study for BMRV1 and OVRV1. Supplementary Table 5 SRA accessions, library and mapping statistics for small RNA sequencing data of *B. malayi* samples.

## Source data

Source Data Figs. 2, 3d and 5a–c Raw data used as input into R for graph drawing.

Source Data Figs. 3a–c,e and 5a,b Unprocessed PCR gels and western blots.

## Data Availability

Assembled virus sequences (all 91 derived from transcriptome assembly and an additional 3 derived from viral metagenomics for OVRV1) have been deposited in the ENA at EMBL-EBI under accession number PRJEB64898. Sequencing data used for viral metagenomics of OVRV1 have been deposited in the ENA at EMBL-EBI under accession number PRJEB67302. Small RNA sequencing data from BMRV1-infected *B. malayi* adults have been deposited in the Sequence Read Archive (SRA) of the NCBI database, under the accession number PRJNA1089951. The transcriptome datasets used to assemble these virus sequences have previously been published, and a full list of transcriptome accessions used for the identification of viruses in this paper has been included as part of Supplementary Table 1. This also includes a list of their associated papers, using their PubMed identification number. All datasets are publicly available. Additional supplementary data are also hosted separately on Figshare, containing the phylogenetic trees used as images in this study in Newick format, as well as the alignments that were used to generate them. Additional phylogenetic trees for the different virus orders identified (both the Newick-formatted trees and the alignments used to generate them) are also available via Figshare. These can be found at 10.6084/m9.figshare.23906130 (ref. ^101^). Additional data for the alignment of all Onchocerca volvulus RNA Virus 1 nucleotide sequences in fasta-file format, plus Onchocerca ochengi RNA Virus 1, are also available via Figshare at 10.6084/m9.figshare.23954781 (ref. ^102^). Source data are provided with this paper.

## Extended data

**Extended Data Table 1:** Overview of nematode species’ transcriptomes surveyed, their definitive hosts, and the viruses family and genome type

Order	Nematode Species (datasets)	Definitive host	Viruses	Virus family (genome strandedness)
Spirurida	*Brugia malayi* (93)	Humans, monkeys, cats, dogs	1	*Togaviridae* (ss+)
	*Brugia pahangi* (36)	Cats, dogs	2	*Partitiviridae* (ds)
	*Loa loa* (1)	Humans	-	-
	*Onchocerca volvulus* (12)	Humans	6	*Rhabdoviridae* (ss-), *Dicistroviridae* (ss+), 3x *Totiviridae* (ds), Unclassified *Bunyavirales* (ss-)
	*Onchocerca ochengi* (3)	Cattle	11	*Rhabdoviridae* (ss-), *Closteoviridae* (ss+), 5x *Totiviridae* (ds), 2x *Fiersviridae* (ss+), *Sedoreoviridae* (ss+), Unclassified *Bunyavirales* (ss-)
	*Dirofilaria immitis* (12)	Dogs, cats	-	-
	*Litomosoides sigmodontis* (5)	Rodents	-	-
	*Gnathostoma spinigerum* (1)	Humans, dogs, cats	-	-
	*Acanthocheilonema viteae* (7)	Rodents	-	-
	*Anguillicola crassus* (48)	Eels	1	*Totiviridae* (ds)
Ascaridida	*Ascaris suum/lumbricoides* (42)	Pigs, humans	4	*Rhabdoviridae* (ss-), 2x *Totiviridae* (ds), *Partitiviridae* (ds)
	*Anisakis simplex* (1)	Sea mammals	-	-
	*Toxocara canis* (4)	Dogs	7	4x *Totiviridae* (ds), 2x *Partitiviridae* (ds), 1x *Flaviviridae* (ss+)
Rhabditida	*Strongyloides stercoralis* (42)	Humans	-	-
	*Strongyloides ratti* (14)	Rodents	-	-
Strongyloida	*Ancylostoma caninum* (4)	Dogs	-	-
	*Ancylostoma ceylanicum* (58)	Humans, dogs, cats	4	*Chrysoviridae* (ds), *Partitiviridae* (ds), *Fiersviridae* (ss+), *Belpaoviridae* (ss)
	*Angiostrongylus cantonensis* (9)	Rodents	2	*Rhabdoviridae* (ss-), *Phenuiviridae* (ss-)
	*Dictyocaulus viviparus* (29)	Cattle	2	2x *Iflaviridae* (ss+)
	*Haemonchus contortus* (36)	Ruminants	2	*Sedoreoviridae* (ds), *Cghuviridae* (ss-)
	*Heligmosomoides polygyrus* (6)	Rodents	3	2x Unclassified *Picornavirales* (ss+), *Chuviridae* (ss-)
	*Necator americanus* (7)	Humans	-	-
	*Nippostrongylus brasiliensis* (15)	Rodents	5	*Flaviviridae* (ss+), *Virgaviridae* (ss+), 3x *Totiviridae* (ds)
	*Oesophagostomum dentatum* (31)	Swine	2	*Totiviridae* (ds), *Phenuiviridae* (ss-)
	*Ostertagia ostertagi* (21)	Ruminants	3	*Nyamiviridae* (ss-), *Totiviridae* (ds), *Partitiviridae* (ds)
	*Teladorsagia circumcincta* (18)	Sheep, goats	1	*Virgaviridae* (ss+)
Trichinellida	*Trichinella spiralis* (7)	Humans, swine, canids	1	*Totiviridae* (ds)
	*Trichinella nelsoni* (4)	Humans, swine, felids	-	-
	*Trichinella murrelli* (4)	Humans, swine, ursine, canids	1	*Totiviridae* (ds)
	*Trichinella britovi* (4)	Humans, swine, canids	1	*Totiviridae* (ds)
	*Trichinella patagoniensis* (5)	Swine, felids	2	*Lispiviridae* (ss-), *Totiviridae* (ds)
	*Trichinella nativa* (4)	Humans, swine, ursine, mustelids	2	*Lispiviridae* (ss-), *Totiviridae* (ds)
	*Trichinella T6* (4)	Humans, swine, ursine, mustelids	1	*Totiviridae* (ds)
	*Trichinella T8* (4)	Swine, felids	1	*Totiviridae* (ds)
	*Trichinella T9* (4)	Swine, canids	1	*Totiviridae* (ds)
	*Trichinella papuae* (4)	Humans, swine, reptiles	-	-
	*Trichinella zimbabwensis* (4)	Swine, reptiles	-	-
	*Trichinella pseudospiralis* (8)	Humans, swine, felids, birds, marsupials	7	*Lispiviridae* (ss-), *Peribunyaviridae* (ss-), 3x *Totiviridae* (ds), 2x Unclassified *Bunyavirales* (ss-)
	*Trichuris muris* (21)	Mouse	10	*Rhabdoviridae* (ss-), 2x *Qinviridae* (ss-), 3x *Totiviridae* (ds), *Solemoviridae* (ss+), *Lispiviridae* (ss-), *Flaviviridae* (ss+), *Peribunyaviridae* (ss-)
	*Trichuris suis* (15)	Swine	5	*Lispiviridae* (ss-), 2x *Totiviridae* (ds), *Peribunyaviridae* (ss-), *Partitiviridae* (ds)
	*Trichuris trichiura* (1)	Humans, primates	3	*Rhabdoviridae* (ss-), *Lispiviridae* (ss-), *Peribunyaviridae* (ss-)

Overview of nematode species’ transcriptomes surveyed, their definitive hosts, and the viruses family and genome type (ss + , positive-sense single stranded, ss-, negative-sense single stranded, ds, double-stranded). “Datasets” refers to the number of individual sequencing libraries analysed, which form one or more Bioproject. See Supplementary Table 1 for more information on the datasets used and associated manuscript Pubmed ID number.

## Code availability

This paper did not generate new software; however, custom code was used for generating the histogram of mapped read lengths and first base pair bias. Bash scripts for these are available via GitHub at github.com/rhparry/viral_sRNA_tools/, 10.5281/zenodo.13312694. Assembly pipelines and bioinformatic analysis of virus genome sequences have been described in Methods and use previously published programs.
