# Supplementary material for: Diverse RNA viruses of parasitic nematodes can elicit antibody responses in vertebrate hosts
Source: Nat Microbiol. 2024 Sep 4;9(10):2488–505. doi: 10.1038/s41564-024-01796-6 (PMC11445058; doi:10.1038/s41564-024-01796-6)

nature microbiology

Supplementary information

https://doi.org/10.1038/s41564-024-01796-6

Diverse RNA viruses of parasitic nematodes can elicit antibody responses in vertebrate hosts

In the format provided by the authors and unedited

Diverse RNA viruses of parasitic nematodes can elicit antibody responses in vertebrate hosts

Shannon Quek[1*§], Amber Hadermann[2*], Yang Wu[1*], Lander De Coninck[3], Shrilakshmi Hegde[1], Jordan R. Boucher[1], Jessica Cresswell[1], Ella Foreman[1], Andrew Steven[1], E. James LaCourse[1], Stephen A. Ward[1], Samuel Wanji[4,5], Grant L. Hughes[6], Edward I. Patterson[7], Simon C. Wagstaff[1], Joseph D. Turner[1], Rhys H. Parry[8], Alain Kohl[6], Eva Heinz[9◊], Kenneth Bentum Otabil[10], Jelle Matthijnssens[3], Robert Colebunders[2], Mark J. Taylor[1§] ✉

1. Centre for Neglected Tropical Diseases, Department of Tropical Disease Biology, Liverpool School of Tropical Medicine, Pembroke Place, Liverpool, L3 5QA, UK.
2. Global Health Institute, University of Antwerp, Doornstraat 331, 2610 Antwerp, Belgium.
3. Laboratory of Viral Metagenomics, Clinical and Epidemiological Virology, Rega Institute, Department of Microbiology, Immunology and Transplantation, KU Leuven, Leuven, Belgium
4. Parasite and Vector Biology Research Unit, Department of Microbiology and Parasitology, Faculty of Science, University of Buea, Buea, Cameroon.
5. Research Foundation for Tropical Diseases and the Environment (REFOTDE), Buea, Cameroon.
6. Centre for Neglected Tropical Diseases, Departments of Tropical Disease Biology and Vector Biology, Liverpool School of Tropical Medicine, Pembroke Place, Liverpool, L3 5QA, UK.
7. Department of Biological Sciences, Brock University, St. Catharines, ON, L2S 3A1, Canada.
8. School of Chemistry and Molecular Biosciences, The University of Queensland, Brisbane, QLD, Australia
9. Departments of Vector Biology and Clinical Sciences, Liverpool School of Tropical Medicine, Pembroke Place, Liverpool, L3 5QA, UK, and Strathclyde Institute of Pharmacy & Biomedical Sciences, University of Strathclyde, Glasgow, G4 0RE, UK
10. Consortium for Neglected Tropical Diseases and One Health, Department of Biological Sciences, University of Energy and Natural Resources, Ghana

*Contributed equally

[◊]Currently based at the Strathclyde Institute of Pharmacy & Biomedical Sciences, University of Strathclyde, Glasgow, UK

[§]Corresponding authors:

Prof. Mark Taylor, Mark.Taylor@lstmed.ac.uk

Dr Shannon Quek, Shannon.Quek@lstmed.ac.uk

Supplementary Data table descriptions

Supplementary Table 1: A full table containing details of viruses identified during this study, the nematode transcriptomes analysed, number of datasets showing an average read depth of >0.5 for the virus, PUBMED ID number of the study that generated the transcriptome, and method of library generation as described in the original publication. *Additional data includes details of top BLAST hits for the different viruses, as well as information on the study that the transcriptome dataset came from. This includes sample preparation and processing methods, such as details of poly-A/mRNA enrichment if performed, as well as where the parasite material was derived from (e.g. field-caught, laboratory colony) where this information was available.*

Supplementary Table 2: Results from CheckV with details on assembly length, total gene count, predicted virus gene count, and predicted sequence completion status.

Supplementary Table 3: Details of* O. volvulus *parasite sample collection, storage and pooling from Cameroon and Ghana. *These samples were used for viral metagenomics to confirm the presence of OVRV1 from parasite material from a different source.*

Supplementary Table 4: SRA accessions, library and mapping statistics for small RNA sequencing data of B. malayi samples.

Supplementary Table 5: Primer and FISH probe sequences used during this study for BMRV1 and OVRV1.

Supplementary Figure

Supplementary Figure 1: Tanglegram of Trichinella spp. and a* Ghabrivirales *RNA virus found within them using midpoint rooted trees. *Phylogenetic tree on the left corresponds to* Trichinella *spp., while the tree on the right corresponds to the identified* Ghabrivirales. *Viruses were not found in T. zimbabwensis, T. papuae, T. patagoniensis or T. nelsoni (grey highlight). T. pseudospiralis and T. spiralis divergence mirrors the phylogenetic analysis of TpseudospiralisRV1, TpseudospiralisRV4, and TspiralisRV1. Bootstrap values are indicated on each branch, and tree scales are located above each tree. Trees are mid-point rooted for clarity only.*

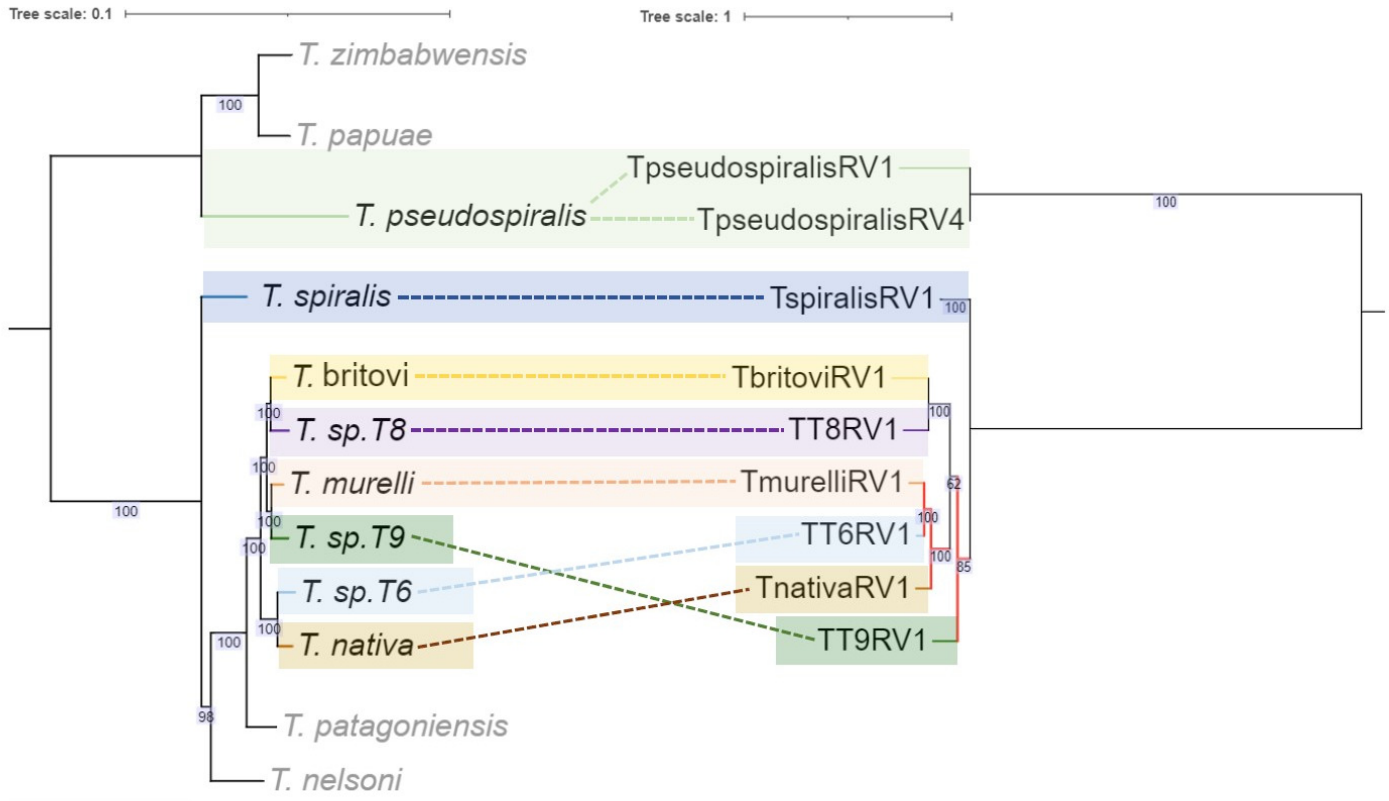

Supplementary Figure 2: Phylogenetic trees for the different virus orders identified as part of this study, and were not deemed as likely contaminants which infect bacteria or plants. *A total of seven different phylogenetic trees were built, for the viral orders: Bunyavirales (evolutionary model LG+F+I+G4), Durnavirales (LG+F+G4) Ghabrivirales (LG+G4), Jingchu-Mononegavirales (LG+F+I+G4), Martellivirales (LG+F+I+G4), Picornavirales (LG+F+I+G4), and Reovirales (LG+F+I+G4). An additional two trees were built for the virus groups: Flaviviridae (LG+I+G4) and Qinviridae (LG+I+G4). See following pages.*

Bunyavirales (LG+F+I+G4)

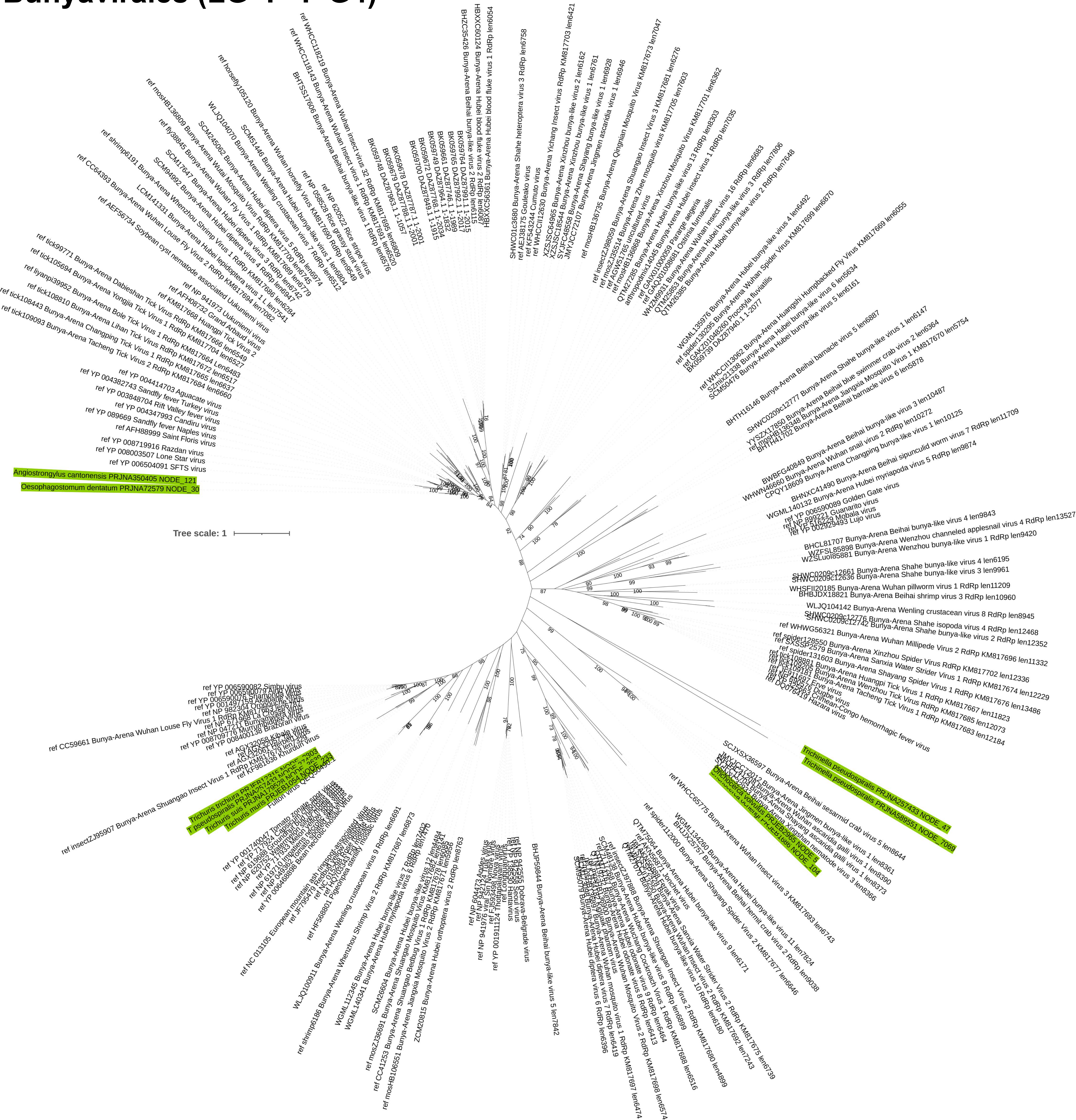

Durnavirales (LG+F+G4)

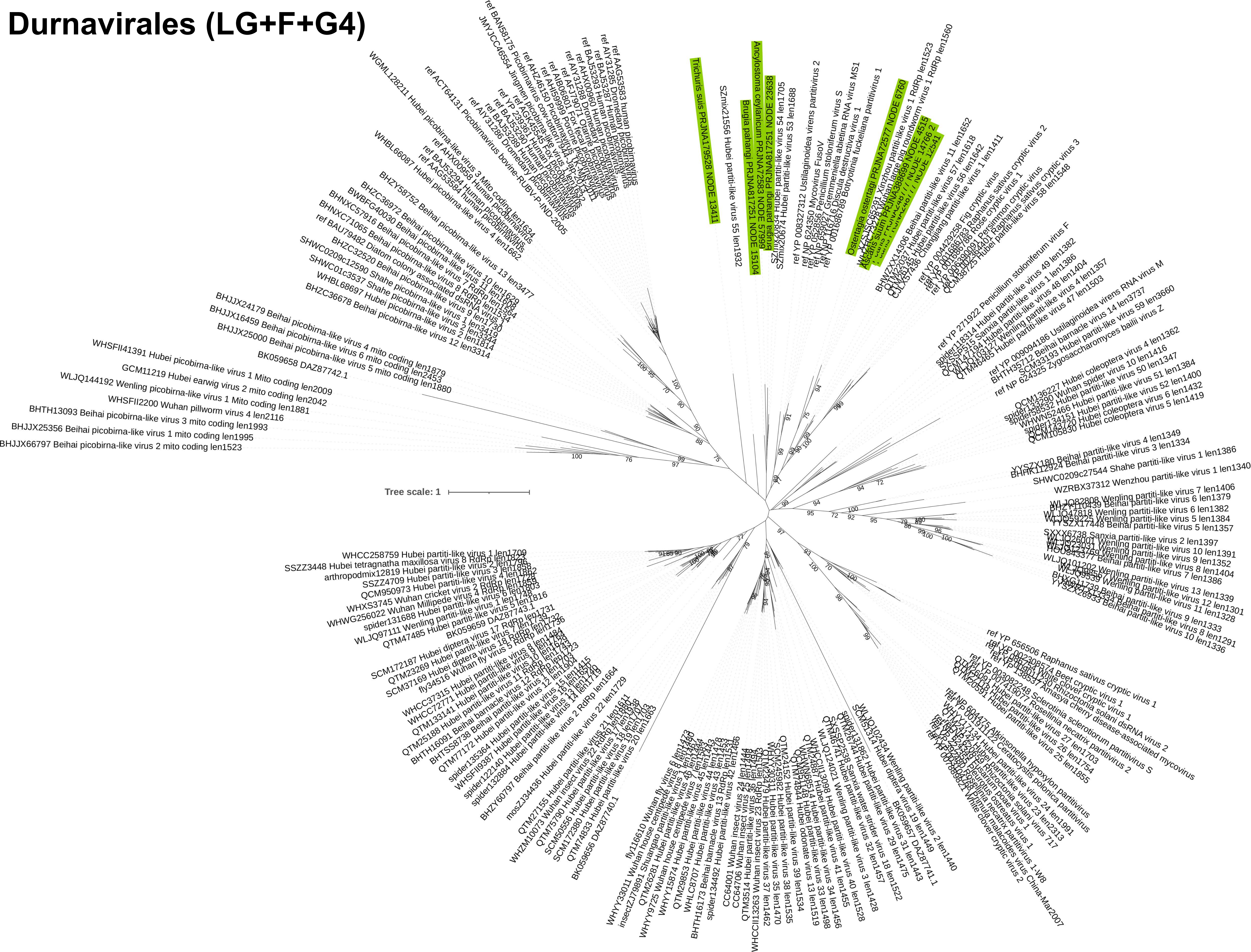

Ghabrivirales (LG+G4)

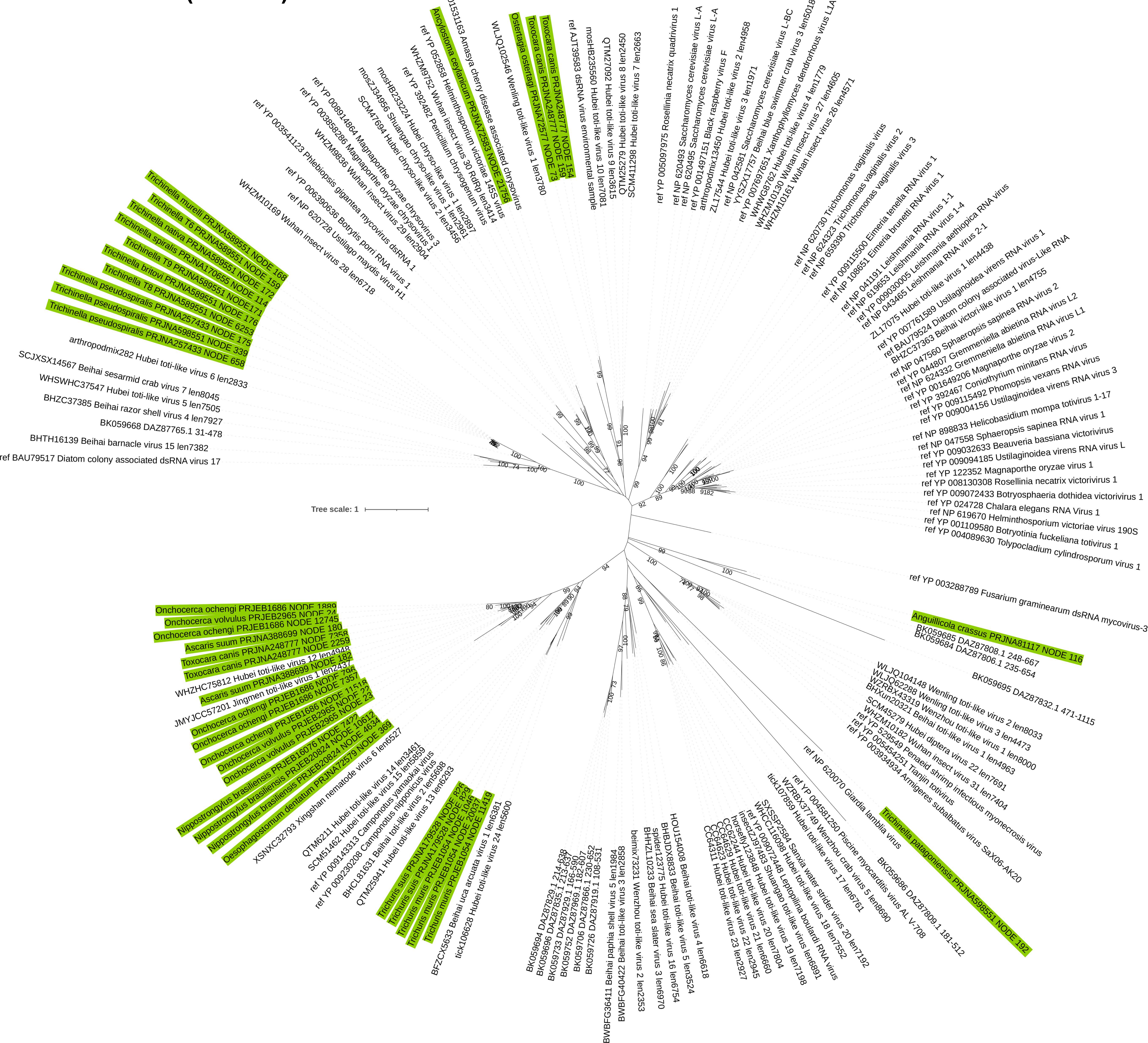

Jingchu-Mononegavirales (LG+F+I+G4)

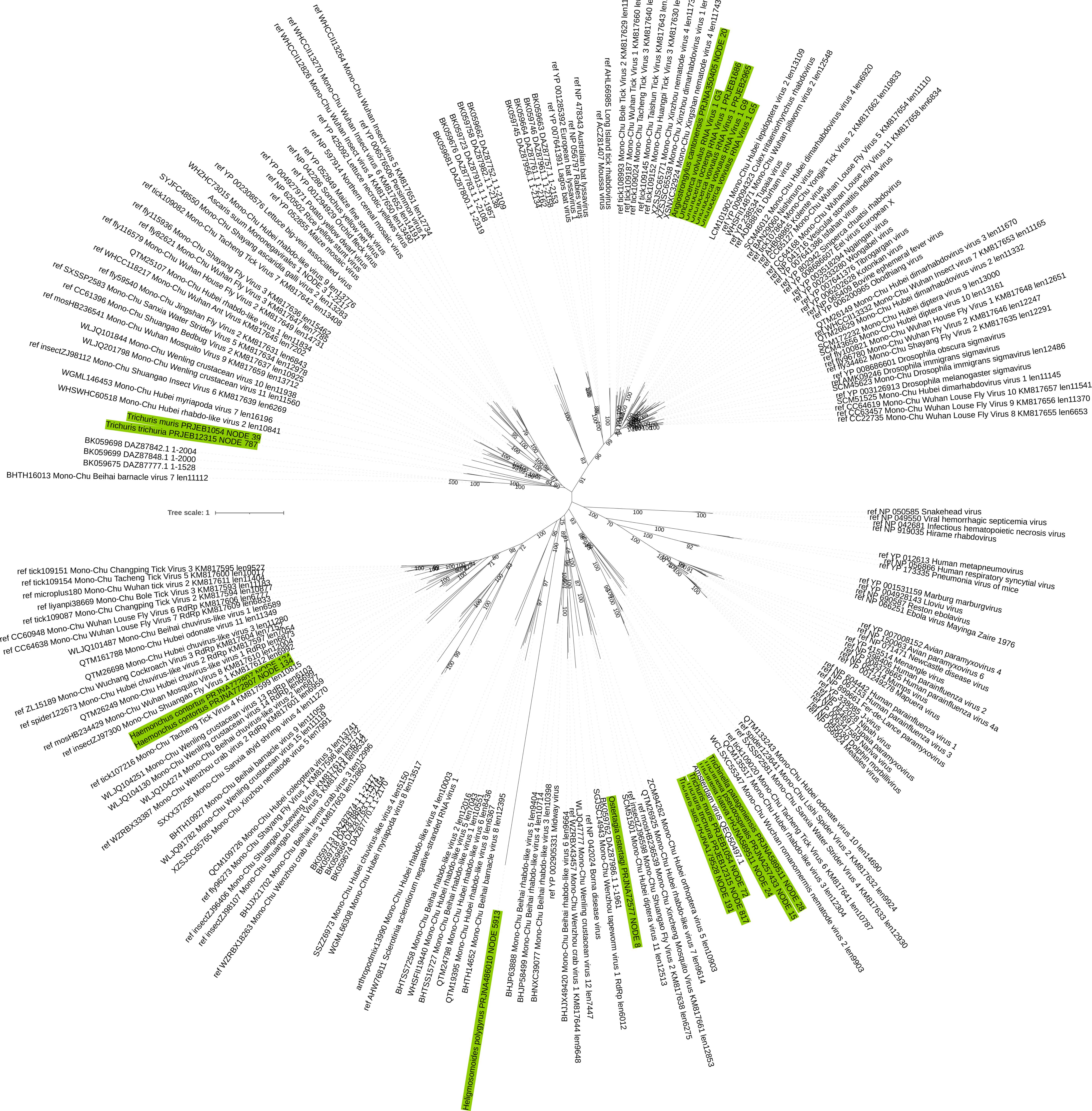

Martellivirales (LG+F+I+G4)

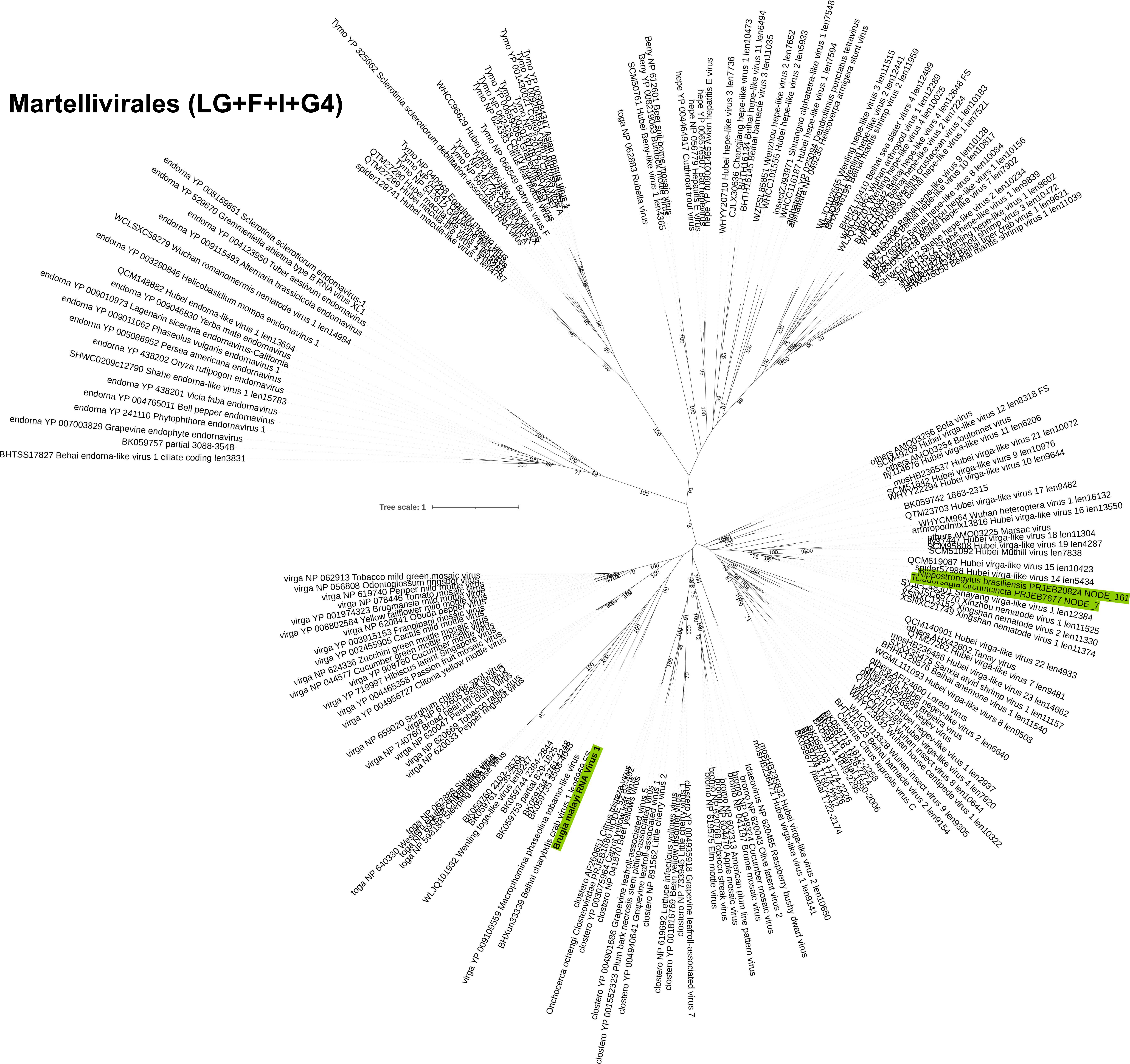

Picornavirales (LG+F+I+G4)

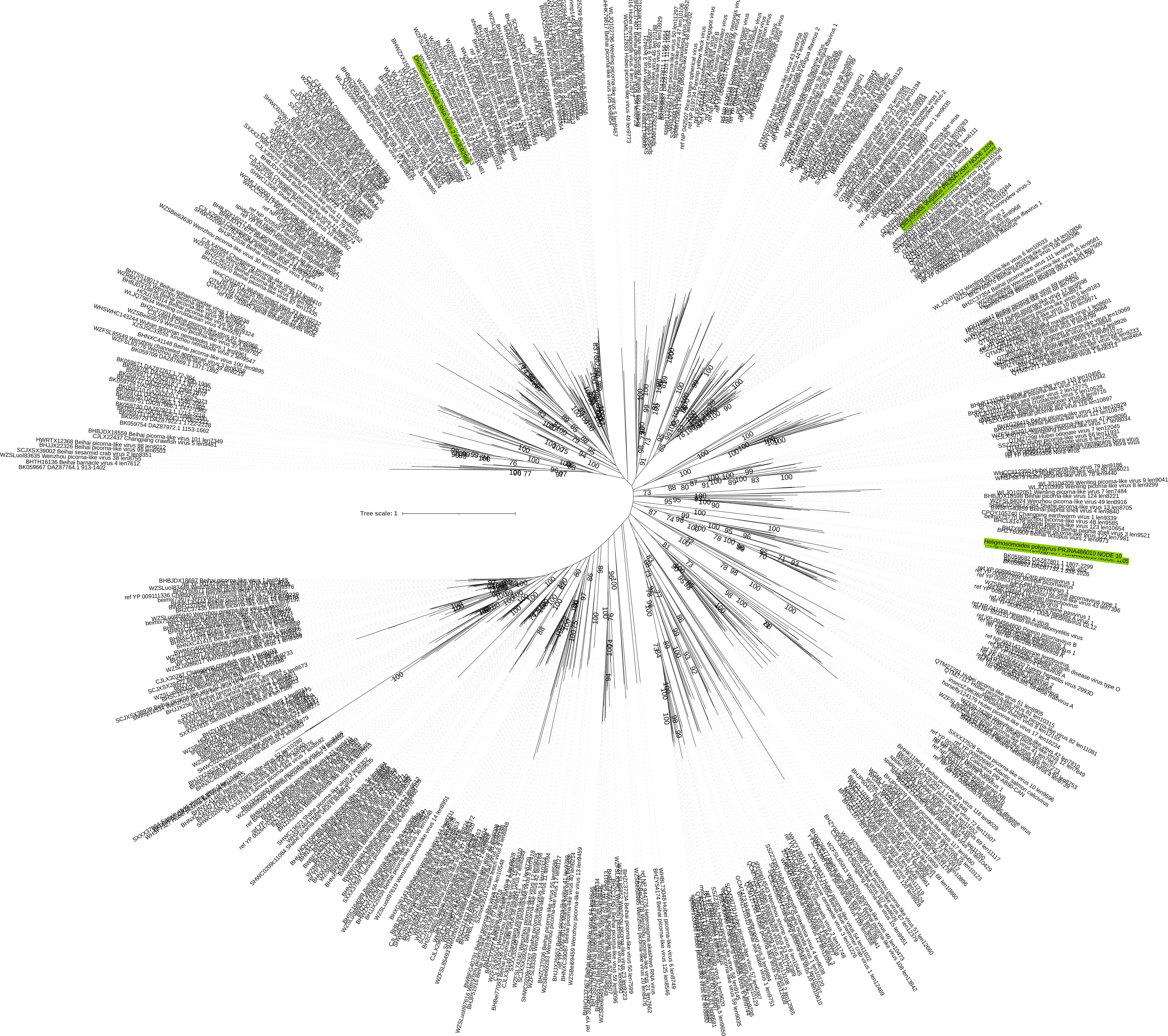

Reovirales (LG+F+I+G4)

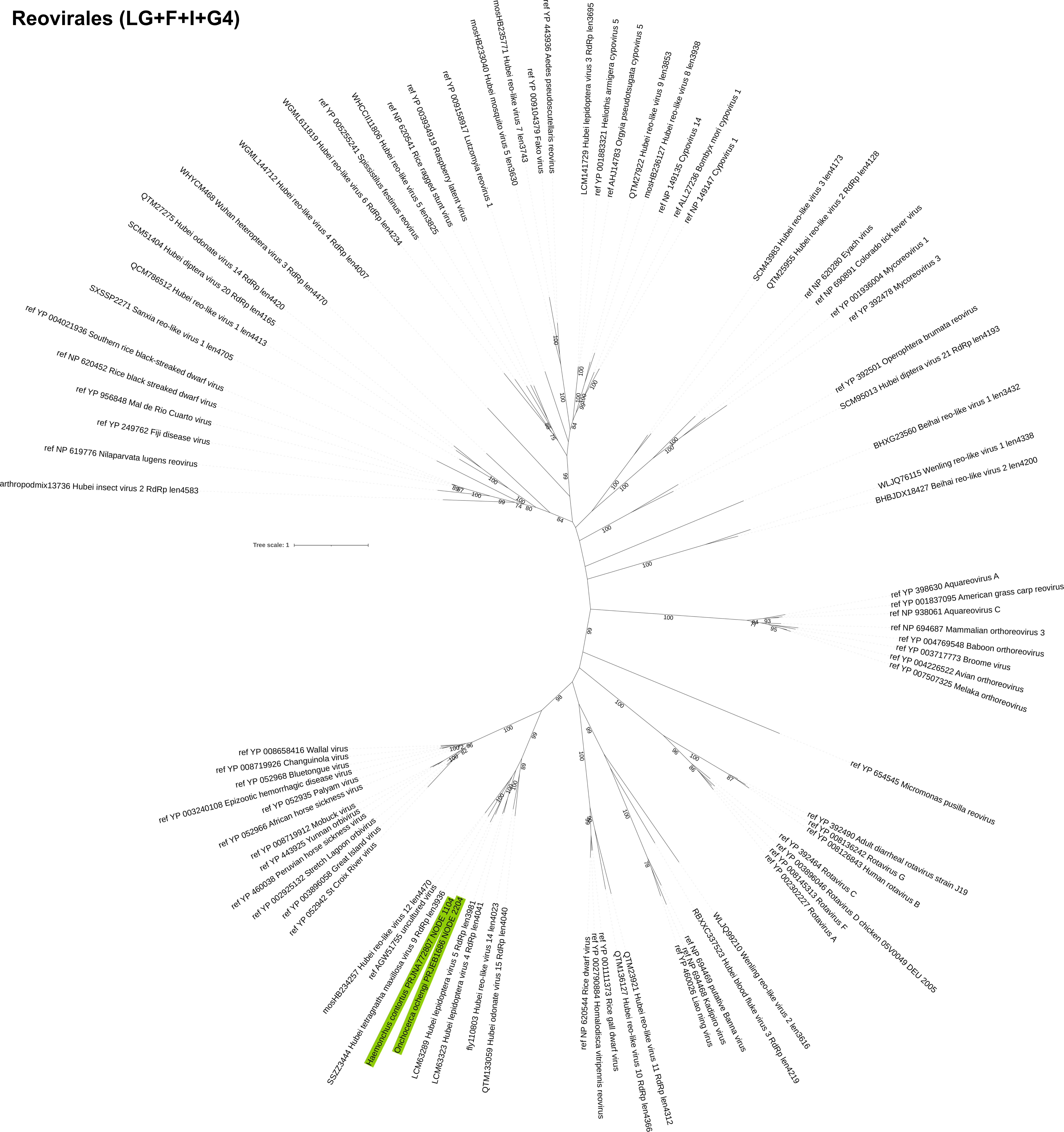

Flaviviridae (LG+I+G4)

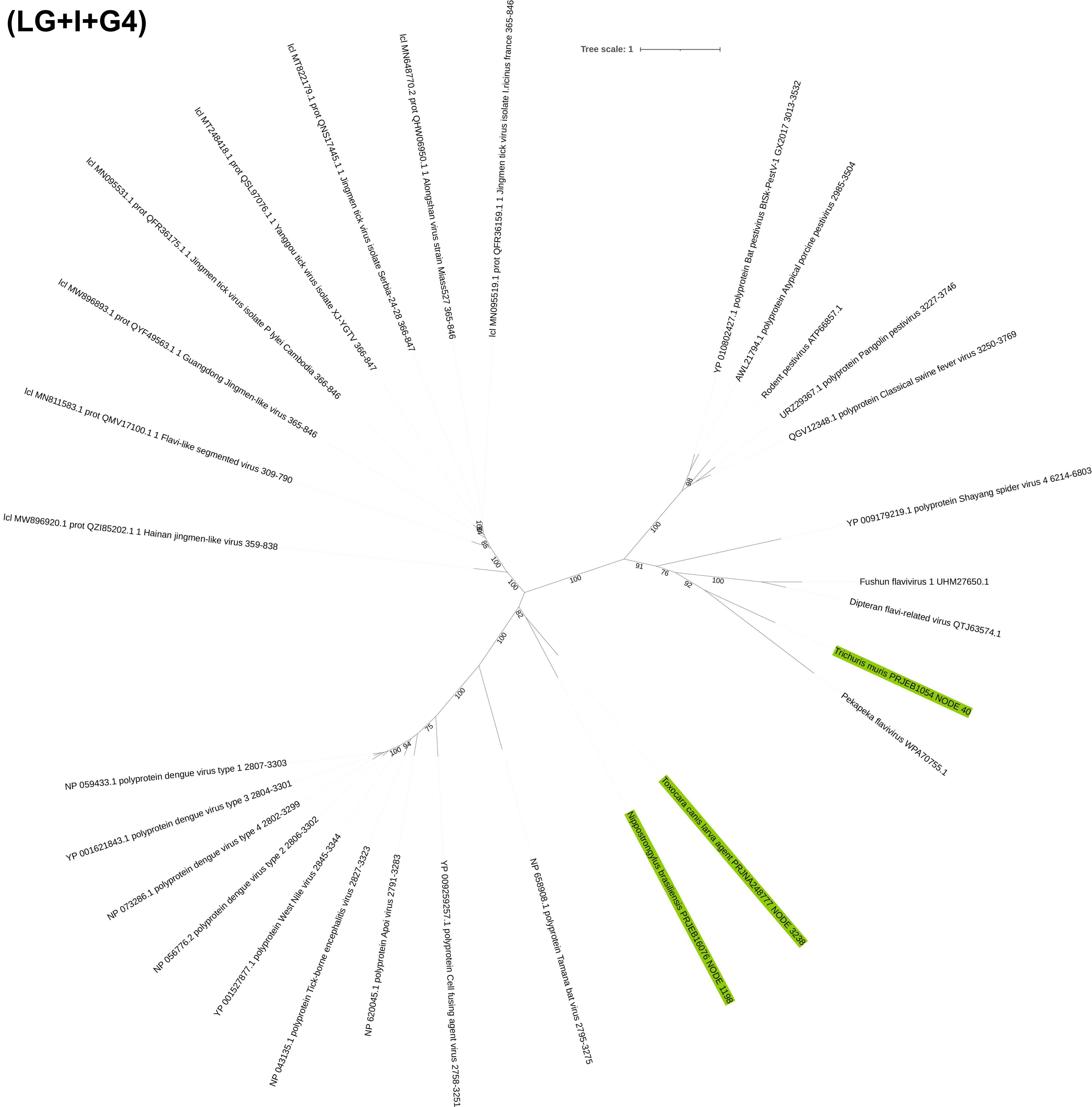

Qinviridae ((LG+I+G4)

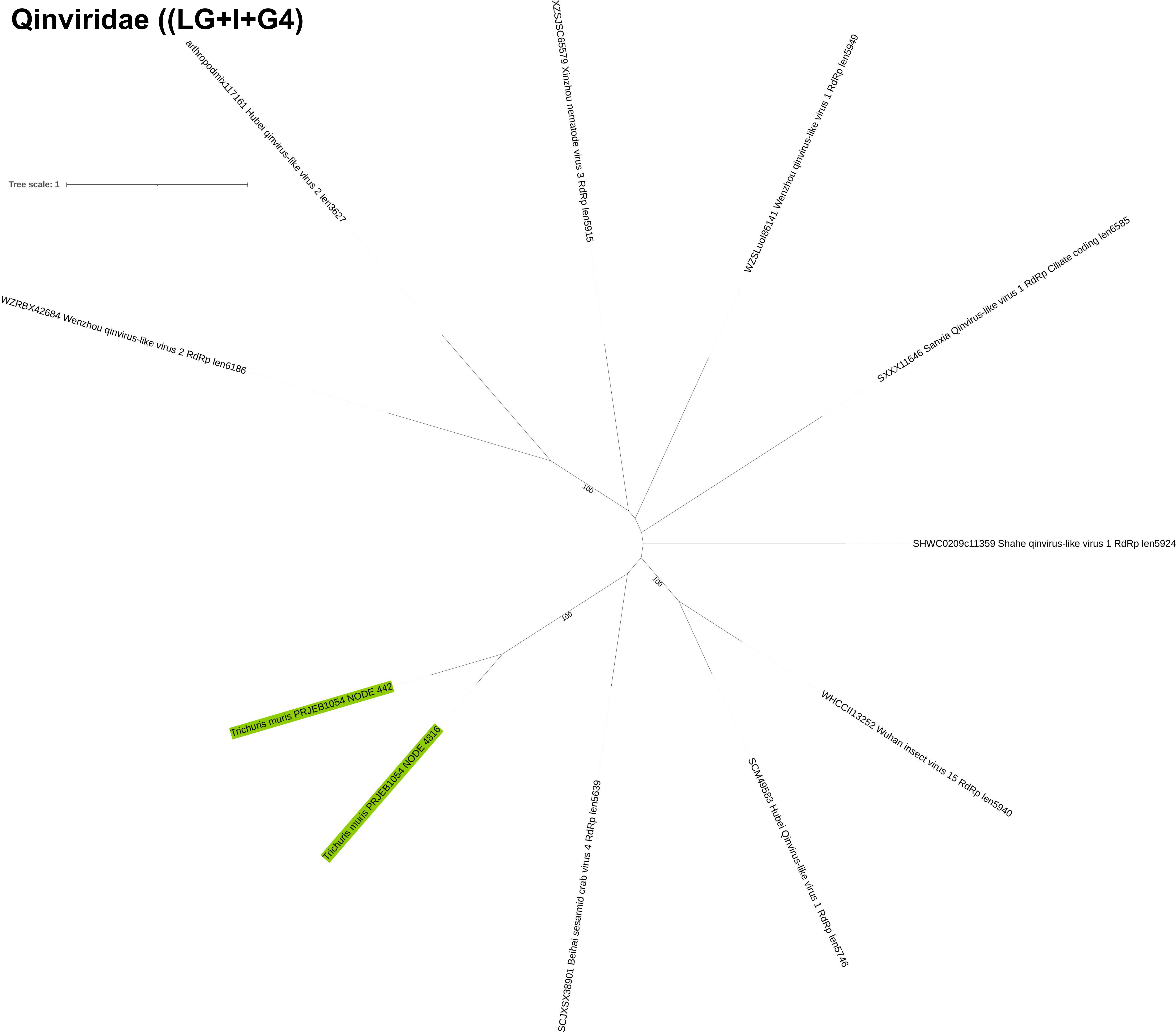

Supplementary Figure 3: ***Read alignment depths for OVRV1, derived from viral metagenomics of* O. volvulus *parasite material collected from locations in Ghana and Cameroon.*** *For each graph, the x-axis represents positions in the respective virus genomes, and the y-axis represents the number of reads from the sequencing dataset used. Metadata on location and sampling included in the grey column on the right side of each graph.* *See following page.*

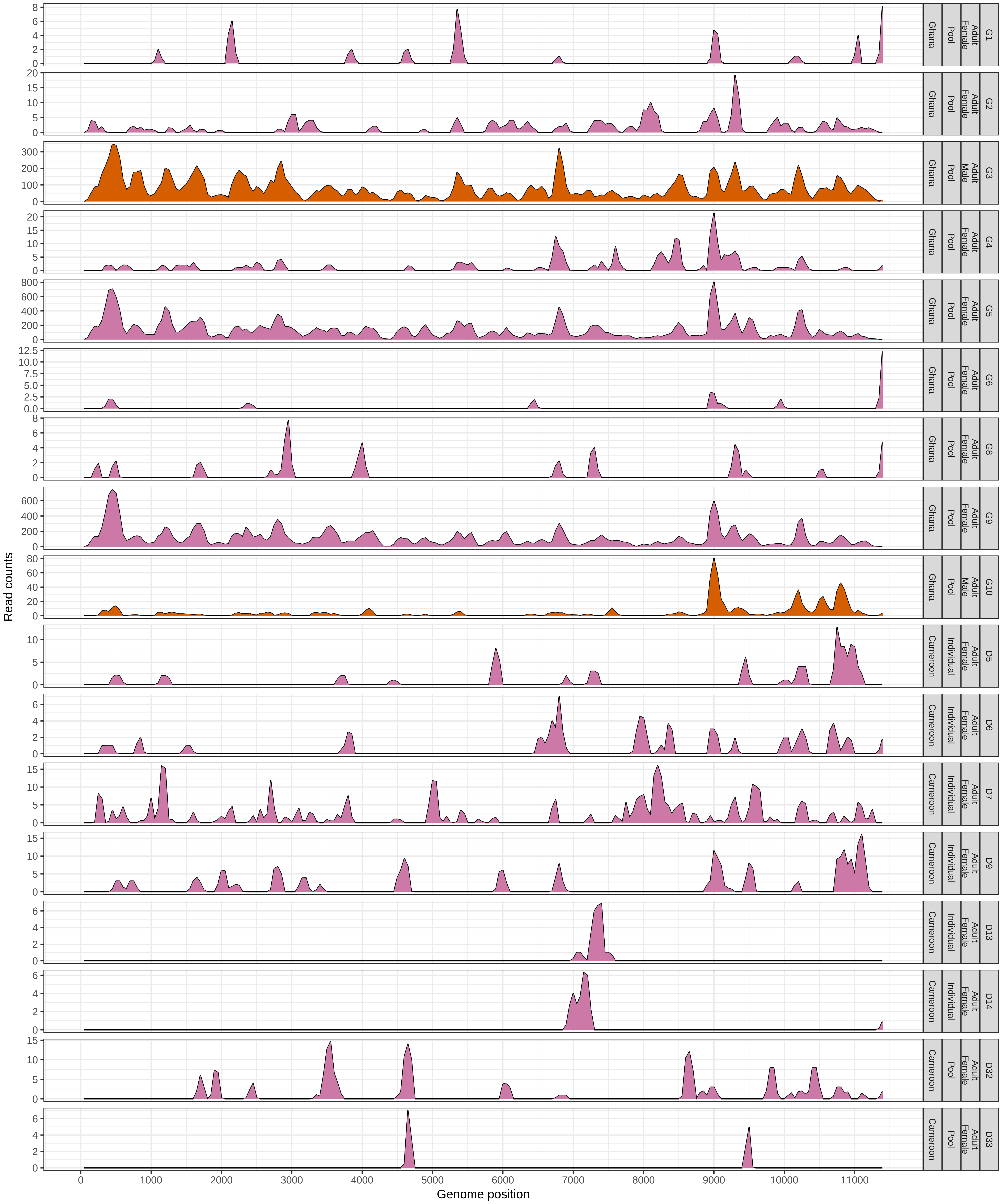
Read counts
Genome position
0
1000
2000
3000
4000
5000
6000
7000
8000
9000
10000
11000
Ghana Pool Adult Female G1
Ghana Pool Adult Female G2
Ghana Pool Adult Male G3
Ghana Pool Adult Female G4
Ghana Pool Adult Female G5
Ghana Pool Adult Female G6
Ghana Pool Adult Female G8
Ghana Pool Adult Female G9
Ghana Pool Adult Male G10
Cameroon Individual Adult Female D5
Cameroon Individual Adult Female D6
Cameroon Individual Adult Female D7
Cameroon Individual Adult Female D9
Cameroon Individual Adult Female D13
Cameroon Individual Adult Female D14
Cameroon Pool Adult Female D32
Cameroon Pool Adult Female D33

Supplementary Figure 4: Alignment of the different strains of OVRV1, derived from* O. volvulus *parasite material collected in Cameroon (1, 'transcriptome') and Ghana (3, 'viral metagenomics'), and* O. ochengi *parasite material collected in Cameroon (1, 'transcriptome'). *See following page.*

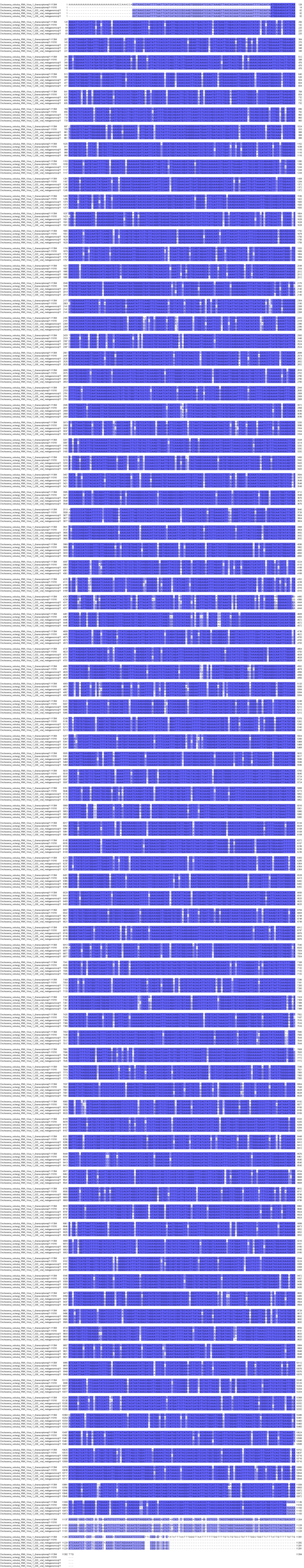
Onchocerca_volvulus_RNA_Virus_1_(transcriptome)/1-11394
Onchocerca_ochengi_RNA_Virus_1_(transcriptome)/1-11016
Onchocerca_volvulus_RNA_Virus_1_(G3,_viral_metagenomics)/1
Onchocerca_volvulus_RNA_Virus_1_(G5,_viral_metagenomics)/1
Onchocerca_volvulus_RNA_Virus_1_(G9,_viral_metagenomics)/1

Supplementary Figure 5: Western blots of excretory-secretory products from adult* B. malayi *nematodes incubated overnight in culture media, showing the presence of BMRV1 capsid proteins. *Note the presence of multiple bands from extracts of both male and female parasites, with the bands appearing in near-regular intervals, suggesting potential multimerization of the 14 kDa band.*

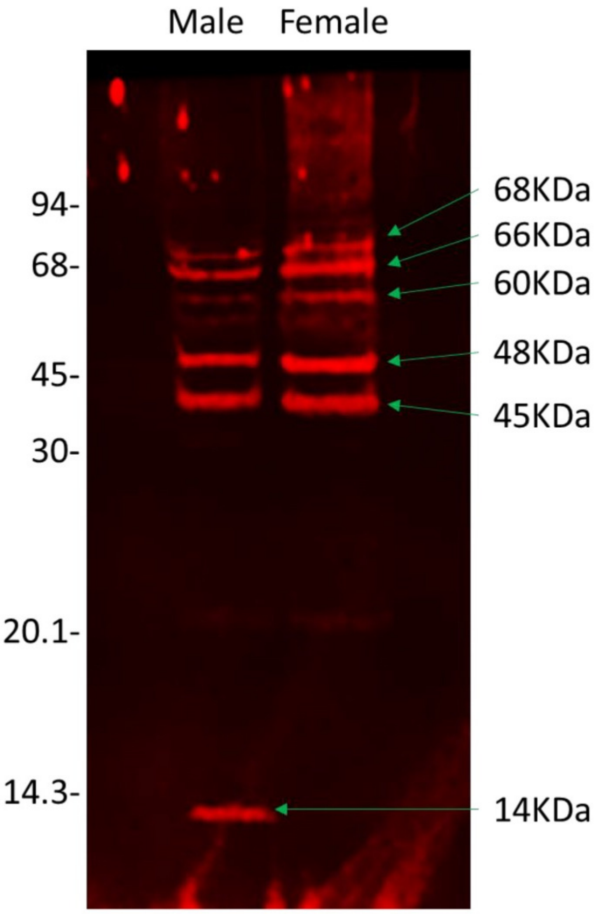

Supplementary Figure 6: PCR of cDNA libraries made from* O. volvulus *parasite material collected in Guatemala, Cameroon (the separate districts of Kumba and Tango) and Mali show presence of OVRV1. *Sequences for the primer sets used can be found in Supplementary Table 3, as well as a description of the target that the primer sets are amplifying in the OVRV1 genome.*

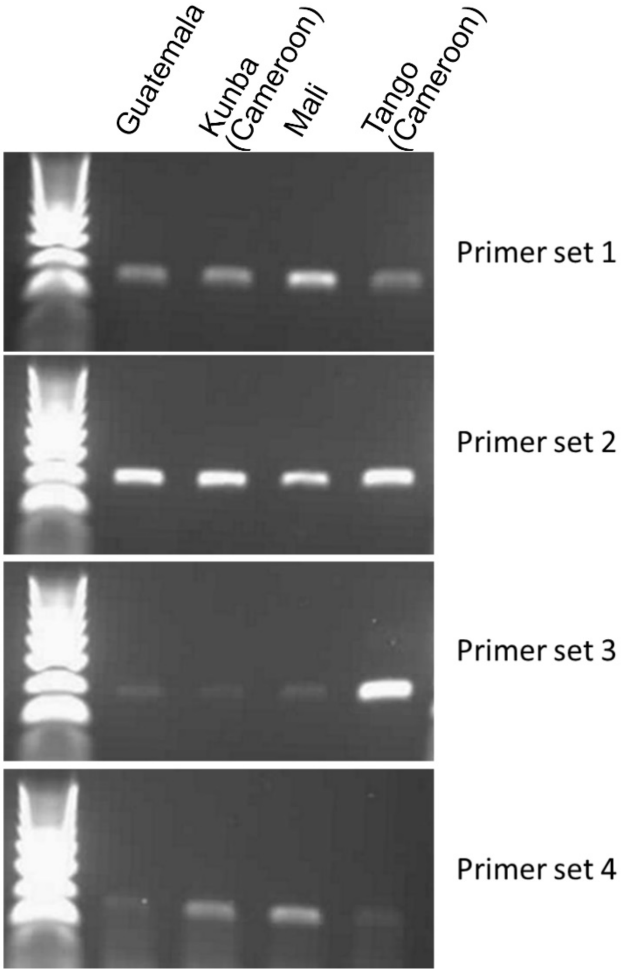

***Supplementary Figure 7*: Western blots of sera derived from jirds uninfected (5) and infected (9) with B. malayi, testing for the presence of antibodies against the BMRV1 capsid protein.** *The serum used for these western blots is the same serum used to generate ELISA data for Figure 5A. Note the variability for BMRV1 capsid antibodies based on western blots. The remaining three blots are negative (1 and 2) and positive (3) controls.*

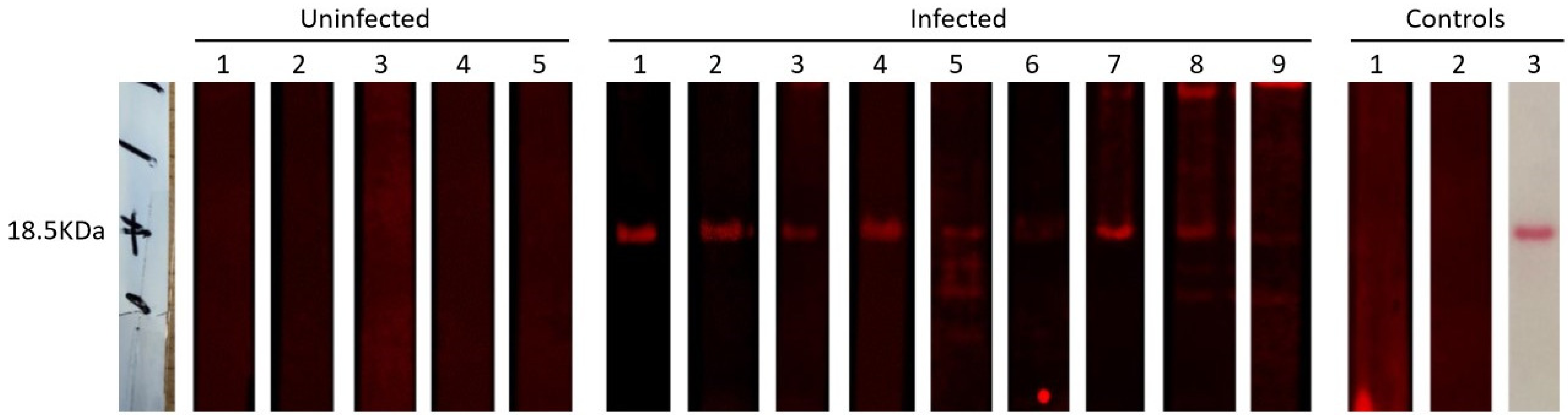

1: No 1st antibody stain
2: No 1st and 2nd stain
3: Positive control stain

Supplementary Source Data Figure 1: *Western blots of excretory-secretory products from adult B. malayi nematodes incubated overnight in culture media, showing the presence of BMRV1 capsid proteins. Used for Supplementary Figure 5. Blue bounding box is the section visualised in Supplementary Figure 5. Molecular ladder included on the far left of image.*

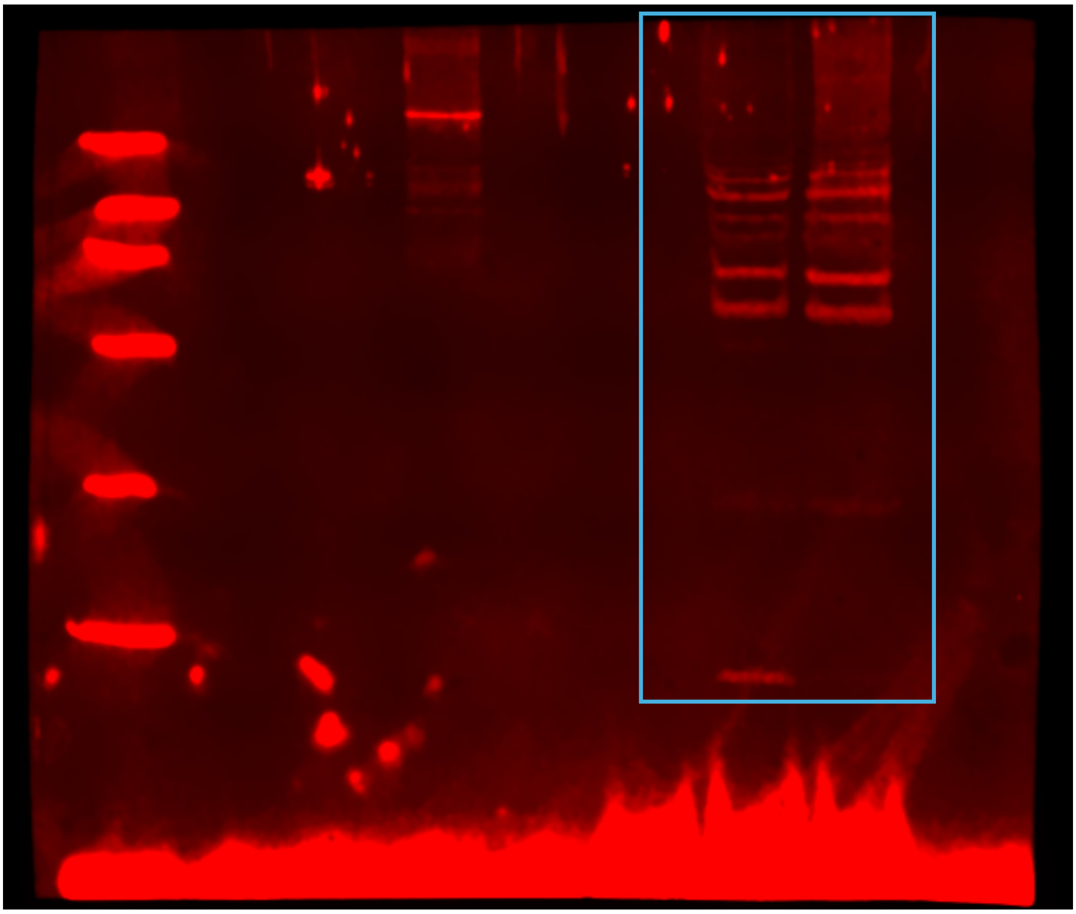

Supplementary Source Data Figure 2: *PCR of cDNA libraries made from O. volvulus parasite material collected in Guatemala, Cameroon (the separate districts of Kumba and Tango) and Mali show presence of OVRV1. Used for Supplementary Figure 6. Blue bounding box is the section visualised in Supplementary Figure 6.*

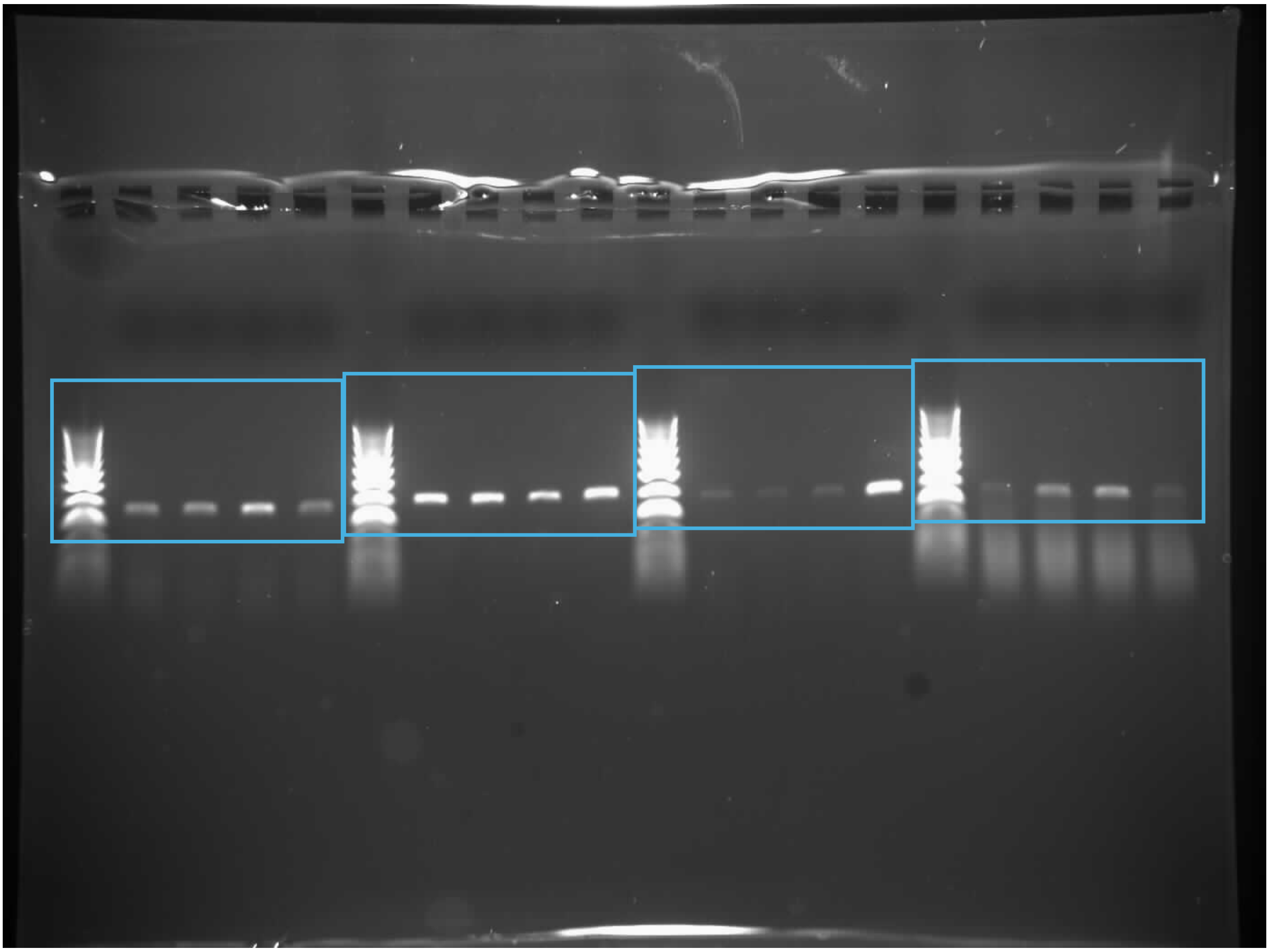

Supplementary Source Data Figure 3: *Western blots of sera derived from jirds uninfected (5) and infected (9) with B. malayi, testing for the presence of antibodies against the BMRV1 capsid protein. This is the clean Ponceau Red stain of the Western blot before cutting and visualisation, with molecular ladder on the left-most side. Used for Supplementary Figure 7. Blue bounding box is the section visualised in Supplementary Figure 7. See Supplementary Source Data Figure 4 for the visualised blot bands.*

We have attempted re-run the samples to generate a clearer image for source data (Supplementary Source Data Figure 5), but could not return signals in all samples similar to Supplementary Source Data Figure 4. This may be due to sample storage, protein aggregation over time, or buffers available to us at the time.

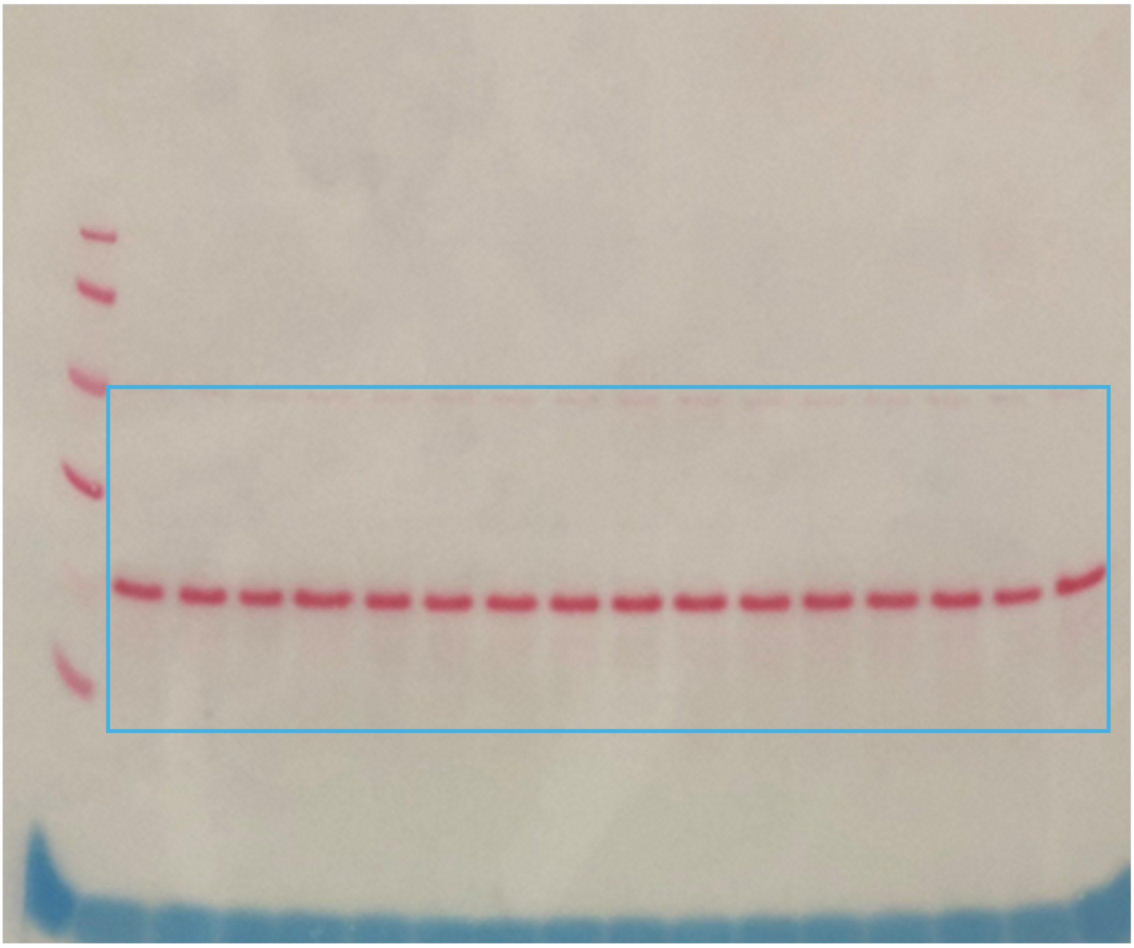

Supplementary Source Data Figure 4: *Western blots of sera derived from jirds uninfected (5, left) and infected (9, middle) with B. malayi, testing for the presence of antibodies against the BMRV1 capsid protein (two negative controls on the right). This is after gel cutting and organised in the same order of appearance from left to right as shown in Supplementary Figure 7. The molecular ladder has been removed, see Supplementary Source Data Figure 3 for a blot with ladder.*

We have attempted re-run the samples to generate a clearer image for source data (Supplementary Source Data Figure 5), but could not return signals in all samples similar to below. This may be due to sample storage, protein aggregation over time, or buffers available to us at the time.

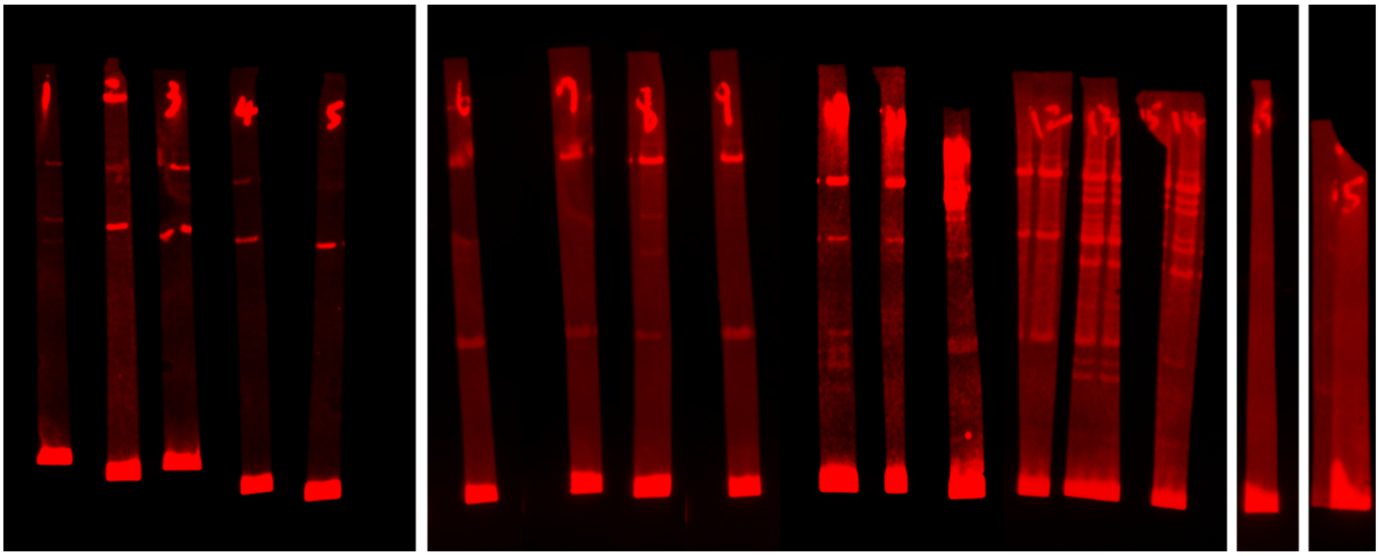

Supplementary Source Data Figure 5: *Western blots of sera derived from jirds infected (9, left) and uninfected (5, right) with B. malayi, alongside a recombinant positive control (in-between the groups), testing for the presence of antibodies against the BMRV1 capsid protein. This is a re-run of the samples shown in Supplementary Figure 7 (samples labelled the same in both figures), including a molecular ladder for all samples. We were not able to replicate bands in all 9 infected jird sera samples, but can observe two correct bands in sample 2 and 4 (blue arrow). Where signals for the capsid protein monomer appear reduced in several samples, a distinct band representing a predicted tetramer as typically found in this virus family remains dominant in all infected sera (grey arrows), but not in uninfected sera.*

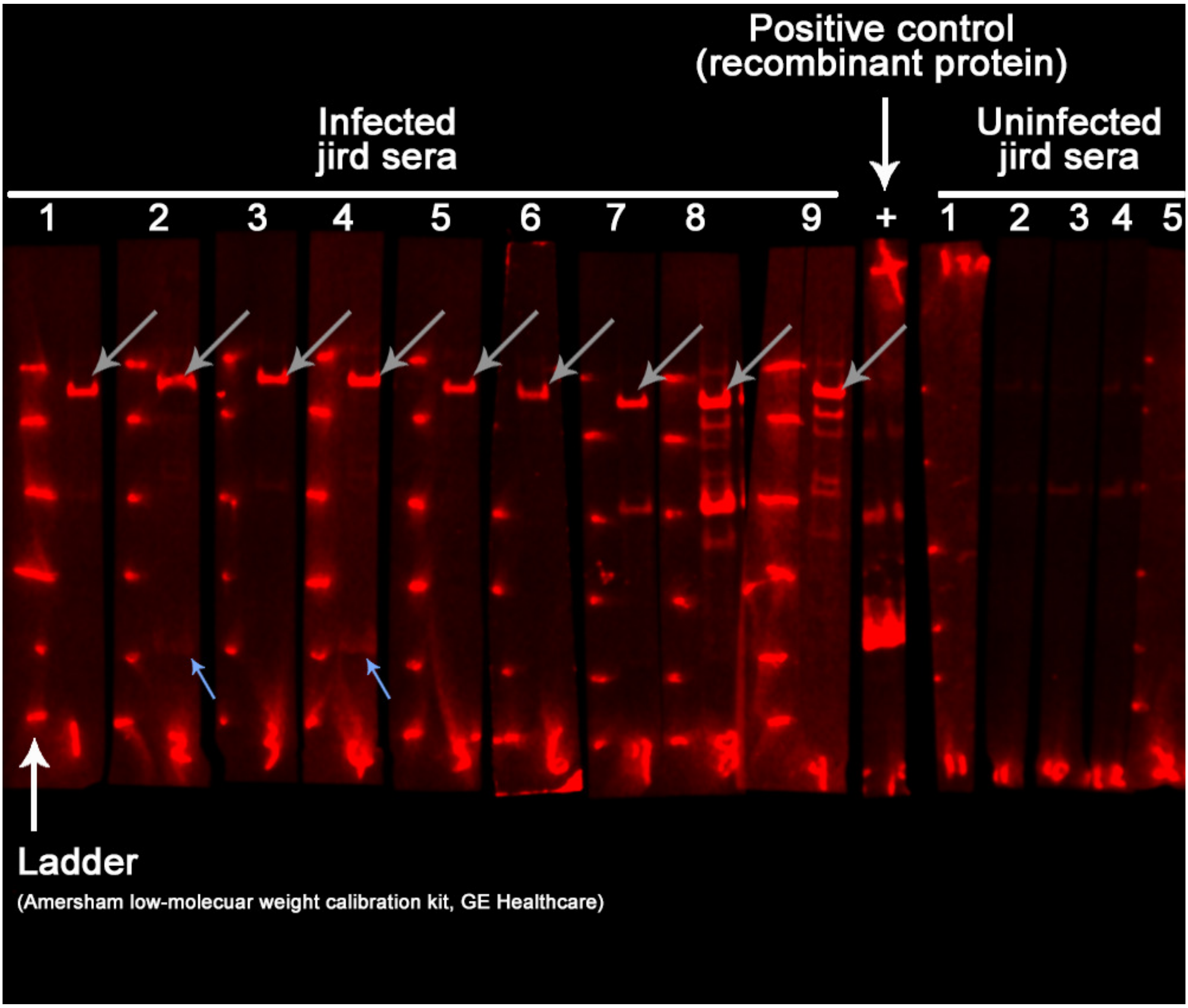

Supplement: Supplementary file 1 — Supplementary Figs. 1–7 and source data for Supplementary Figs. 5–7. [file 41564_2024_1796_MOESM1_ESM.pdf]
