# Supplementary material for: Diverse RNA viruses of parasitic nematodes can elicit antibody responses in vertebrate hosts
Source: Nat Microbiol. 2024 Sep 4;9(10):2488–505. doi: 10.1038/s41564-024-01796-6 (PMC11445058; doi:10.1038/s41564-024-01796-6)
Supplement: Supplementary file 2 — Reporting Summary [file 41564_2024_1796_MOESM2_ESM.pdf]

nature portfolio

Corresponding author(s): Mark Taylor <Mark.Taylor@lstmed.ac.uk> Shannon Quek <Shannon.Quek@lstmed.ac.uk>

Last updated by author(s): Jun 20, 2024

Reporting Summary

Nature Portfolio wishes to improve the reproducibility of the work that we publish. This form provides structure for consistency and transparency in reporting. For further information on Nature Portfolio policies, see our Editorial Policies and the Editorial Policy Checklist.

Statistics

For all statistical analyses, confirm that the following items are present in the figure legend, table legend, main text, or Methods section.

n/a	Confirmed	
☐	☒	The exact sample size (*n*) for each experimental group/condition, given as a discrete number and unit of measurement
☐	☒	A statement on whether measurements were taken from distinct samples or whether the same sample was measured repeatedly
☐	☒	The statistical test(s) used AND whether they are one- or two-sided<br>*Only common tests should be described solely by name; describe more complex techniques in the Methods section.*
☐	☒	A description of all covariates tested
☐	☒	A description of any assumptions or corrections, such as tests of normality and adjustment for multiple comparisons
☒	☐	A full description of the statistical parameters including central tendency (e.g. means) or other basic estimates (e.g. regression coefficient) AND variation (e.g. standard deviation) or associated estimates of uncertainty (e.g. confidence intervals)
☐	☒	For null hypothesis testing, the test statistic (e.g. *F*, *t*, *r*) with confidence intervals, effect sizes, degrees of freedom and *P* value noted<br>*Give P values as exact values whenever suitable.*
☒	☐	For Bayesian analysis, information on the choice of priors and Markov chain Monte Carlo settings
☒	☐	For hierarchical and complex designs, identification of the appropriate level for tests and full reporting of outcomes
☒	☐	Estimates of effect sizes (e.g. Cohen's *d*, Pearson's *r*), indicating how they were calculated

Our web collection on statistics for biologists contains articles on many of the points above.

Software and code

Policy information about availability of computer code

Data collection

ENA Browser Tools application (v1.5.3).
Zeiss LSM 880, AxioObserver.

Data analysis

Zeiss LSM 880, AxioObserver.
Zeiss ZenBLUE Edition (v3.7.97.02000).
bbduk (v38.79).
SPAdes pipeline, specifically the rnaviralSPAdes module (v.3.15.3).
Virsorter2 (v.2.2.3).
BlastX and BlastN against NCBI's virus database.
PROKKA (v.1.11).
InterProScan, https://www.ebi.ac.uk/interpro/search/sequence/.
MAFFT (v.7.505).
Trimal (v1.2rev59).
IQTree (v.1.6.1).
ModelFinder (as bundled with IQTree v.1.6.1).
The Interactive Tree of Life, https://itol.embl.de/.
BWA-MEM, run as part of the NVIDIA Clara Parabricks toolkit71 (v4.0.1-1).
MosDepth (v0.3.2).
RStudio (v2023.06.0 Build 421).
R (v4.3.0).
ggplot2 (v3.4.2).

gggenes package (v0.5.0).
ggbeswarm (v.0.7.2)
viridis (0.6.5).
ggsignif (v.0.6.4).
CheckV (v1.0.1).
geNomad (v1.6.1).
Artemis (v16.0.17).
Orthofinder (v2.5.1).
MosDepth (v0.3.2).
Trimmomatic (v.0.39).
Bowtie2 (v.2.4.2).
Samtools (v.1.15).
bcftools (v.1.8).
Bedtools (v.2.29.2).
Bedtools genome coverage (v.2.27.1)
Geneious Prime (v.2023.1.2).
GraphPad Prism (v.10.0.2).

For manuscripts utilizing custom algorithms or software that are central to the research but not yet described in published literature, software must be made available to editors and reviewers. We strongly encourage code deposition in a community repository (e.g. GitHub). See the Nature Portfolio guidelines for submitting code & software for further information.

Data

Policy information about availability of data

All manuscripts must include a data availability statement. This statement should provide the following information, where applicable:

- Accession codes, unique identifiers, or web links for publicly available datasets
- A description of any restrictions on data availability
- For clinical datasets or third party data, please ensure that the statement adheres to our policy

Assembled virus sequences (all 9175 derived from transcriptome assembly, and an additional 3 derived from viral metagenomics for OVRV1) have been deposited in the European Nucleotide Archive (ENA) at EMBL-EBI under accession number PRJEB64898.
Sequencing data used for viral metagenomics of OVRV1 has been deposited in the ENA at EMBL-EBI under accession number PRJEB67302.
Small RNA sequencing data from BMRV1 infected B. malayi adults have been deposited in the Sequence Read Archive (SRA) of the NCBI database, under the accession number PRJNA1089951.
Additional supplementary data is also hosted separately on Figshare, containing the phylogenetic trees used as images in this study in Newick format, as well as the alignments that were used to generate them. Additional phylogenetic trees for the different virus orders identified (both the Newick formatted trees and the alignments used to generate them) are also available on Figshare. This can be found at: http://doi.org/10.6084/m9.figshare.23906130
Sequence data used to help build the above phylogenetic analysis came from a preexisting alignment used for a wide-ranging study of virus phylogenetics in invertebrates, reference "Shi, M. et al. Redefining the invertebrate RNA virosphere. Nature 540, 539–543 (2016)".
Additional data for the alignment of all Onchocerca volvulus RNA Virus 1 nucleotide sequences in fasta-file format, plus Onchocerca ochengi RNA Virus 1, have also been uploaded to Figshare and can be found at: http://doi.org/10.6084/m9.figshare.23954781
Additional code for the analysis of small RNA-sequencing data can be found github.com/rhparry/viral_sRNA_tools/3_bam_sRNA_histogram.sh.
Datasets that were mined for virus/virus-like sequences have been included in Supplementary Table 1, but are also listed below:
PRJNA284514
PRJNA81117
PRJNA772674
PRJNA303984
PRJNA388112
PRJNA817251
PRJEB2593
PRJNA80937
PRJNA281132
PRJNA263509
PRJNA502990
PRJEB2240
PRJNA60555
PRJEB2965
PRJEB1686
PRJNA388699
PRJNA312925
PRJNA316941
PRJNA248777
PRJNA72583
PRJNA350405
PRJNA72587
PRJNA772807
PRJNA486010
PRJEB16076
PRJEB20824
PRJNA72579
PRJNA72577
PRJEB7677
PRJEB31116
PRJEB5184

PRJEB1376
PRJNA72585
PRJNA72135
PRJNA589551
PRJNA589551
PRJNA257433
PRJNA589551
PRJNA257433
PRJNA170655
PRJNA257433
PRJEB12315
PRJNA179528
PRJEB1054

Research involving human participants, their data, or biological material

Policy information about studies with human participants or human data. See also policy information about sex, gender (identity/presentation), and sexual orientation and race, ethnicity and racism.

Reporting on sex and gender	Sex data was only collected for human serum samples from the Edna McConnell Clark Foundation and Center for Disease Control, as well as the nodules collected from human patients used for immunofluorescence antibody staining. This information was not used in the study design however, as the primary objective of the analysis was to determine the population-based positivity of viral infection (either via using serology against viral antigens, sequencing, or RT-PCR). At this stage of investigation, there is no rationale to determine viral infection positive/negative rates, or population dynamics, based on human host sex-based differences. Consent has not been provided for sharing of disaggregated sex data. Overall numbers for the data collected by the Edna McConnell Clark Foundation (used for ELISA): 192 males, 154 females, 29 undefined/records lost.<br>See reference: "Cook, J. A., Steel, C. & Ottesen, E. A. Towards a vaccine for onchocerciasis. Trends Parasitol. 17, 555–558 (2001)." for more details.<br><br>See reference: "Turner, J. D. et al. Macrofilaricidal activity after doxycycline only treatment of Onchocerca volvulus in an area of Loa loa co-endemicity: A randomized controlled trial. PLoS Negl. Trop. Dis. 4, (2010)." for details on human participants who gave nodules used for immunofluorescence antibody staining (only nodules from a placebo treated group were used).
Reporting on race, ethnicity, or other socially relevant groupings	Only information on the country and/or village of residence (i.e. the country where the participant lived in when the study was performed) was utilised in the analysis performed in this manuscript. Race and ethnicity was not reported as these social groupings were not relevant to the study.<br>Participants were classed as being resident in a particular country and/or village based on the location of their current residence within a country's geographic borders (defined at the time of study completion). This classification was used to allow some general identification on distribution of virus in context of the global distribution of the parasite host.<br>See reference: "Cook, J. A., Steel, C. & Ottesen, E. A. Towards a vaccine for onchocerciasis. Trends Parasitol. 17, 555–558 (2001)." for more details on samples collected as part of the Edna McConnell Clark Foundation study.<br><br>See reference: "Turner, J. D. et al. Macrofilaricidal activity after doxycycline only treatment of Onchocerca volvulus in an area of Loa loa co-endemicity: A randomized controlled trial. PLoS Negl. Trop. Dis. 4, (2010)." for more details on samples collected for immunofluorescence antibody staining.<br><br>Additional data on country of sampling for O. volvulus parasite material from human patients for viral metagenomics is available in supplementary table 3.
Population characteristics	The only relevant consideration for population characteristics utilises data from the Edna McConnell Clark Foundation, which characterised individuals as 'susceptible' to infection with Onchocerca volvulus parasites, or 'putative immunes', which show no signs of infection with parasites despite living in endemic areas for >20 years and with no participation in anthelminthic mass drug administration regimens. This data was used for analysis in Figure 5, and described in the manuscript methods. Human serum samples from the Edna McConnell Clark Foundation and the Center for Disease Control come from Cameroon (n=200), Togo (n=67), Nigeria (n=54), Uganda (n=88) and Ecuador (n=54). Sampled individuals ranged in age from 3 to 95, median age per country was 51, 35, 39, 33, and 33 respectively, with male:female ratios of 98:82, 31:27, 42:12, and 21:33 respectively (where this data was given, Uganda had no information on participant gender).
Recruitment	Inclusion/exclusion criteria for human participants who donated Onchocerca volvulus nodules or parasite material for immunofluorescence antibody staining or PCR can be found in "Turner, J. D. et al. Macrofilaricidal activity after doxycycline only treatment of Onchocerca volvulus in an area of Loa loa co-endemicity: A randomized controlled trial. PLoS Negl. Trop. Dis. 4, (2010)."<br><br>Inclusion criteria for human participants who donated O. volvulus parasite material for viral metagenomics were based on adults aged 18-60 years old that had palpable onchocerciasis nodules.
Ethics oversight	All animal experiments were approved by the ethical committees of the University of Liverpool and Liverpool School of Tropical Medicine (LSTM) and conducted under Home Office Animals (Scientific Procedures) Act 1986 (UK) requirements. Animals had free access to food and water throughout the duration of studies, checked daily for welfare and weighed weekly.<br><br>Human serum was obtained from patients enrolled in a double-blind placebo-controlled randomized clinical trial conducted in Cameroon and was approved by Ethics Committees of the Tropical Medicine Research Station, Kumba, Cameroon, and the Research Ethics Committee of The Liverpool School of Tropical Medicine, Liverpool, UK and NHS National Research Ethics

Service (09/H1001/81, Northwest 4 REC). Written informed consent was obtained from all participants, with the exception of those who were illiterate, where a literate witness signed on behalf of the participant and the participant added a thumbprint (trials registry, no: ISRCTN48118452). Human serum from Uganda, Cameroon, Togo, Nigeria and Ecuador were obtained from the NIH NAID Filariasis Resaearch Resource Reagent Center (FR3, http://www.filariasiscenter.org/), which provides blanket approval for the research community to use as part of a NIAID-funded sample repository. These samples were curated by the Edna McConnell Clark Foundation (EMCF) in 198553, and the CDC onchocerciasis serum bank. Ethical approval for use of UK uninfected control sera was obtained from the NHS Research Ethics Committee (16/NW/0170) and the Central Liverpool Research Ethics Committee (protocol number: UoL001207).

Ethical clearance for the collection of O. volvulus parasite material from Cameroon was obtained from Prof. Stephen Mbigha Ghogomu of the Ethics Review and Consultancy Committee, Cameroon Bioethics Initiative, P.O. Box 31489, Biyem-Assi, Yaoundé, Cameroon, reference number CBI/ 443/ ERCC/CAMBIN. Ethical clearance for the collection of O. volvulus parasite material from Ghana was obtained via a full board review from the Kintampo Health Research Centre Institutional Ethics Committee, Ghana, study ID number KHRCIEC/2018-18. Written informed consent was obtained from all participants, apart from those who were illiterate, where a thumbprint was used instead.

Note that full information on the approval of the study protocol must also be provided in the manuscript.

Field-specific reporting

Please select the one below that is the best fit for your research. If you are not sure, read the appropriate sections before making your selection.

☒ Life sciences ☐ Behavioural & social sciences ☐ Ecological, evolutionary & environmental sciences

For a reference copy of the document with all sections, see nature.com/documents/nr-reporting-summary-flat.pdf

Life sciences study design

All studies must disclose on these points even when the disclosure is negative.

Sample size	Sample size calculations were not possible to generate, as the viruses identified in this study were completely novel, and no prior knowledge of their distribution is available. Additionally, the limited amounts of material available for the parasites being worked on due to constraints with ethics and logistics precluded an extensive analysis to infer virus population dynamics. This study utilised all of this limited biological material we had available at the current time.
Data exclusions	A total of 30 data points were excluded from the ELISA assays. These were from Ugandan samples specifically (out of 88), and were excluded from analysis because they laid above the range of the standard curve used in the study.
Replication	All observations were replicated with a minimum of two or more biological replicates where parasite material was available. All attempts at replication were successful.
Randomization	Randomization was not relevant for this study, as no particular experimental variable/covariate was being tested. We were only interested in the presence or absence of novel viruses in datasets or parasite material that is currently available to us.
Blinding	Blinding was not relevant for this study, as this work was focused on analysing the presence or absence of novel viruses in material where no prior knowledge is available.

Reporting for specific materials, systems and methods

We require information from authors about some types of materials, experimental systems and methods used in many studies. Here, indicate whether each material, system or method listed is relevant to your study. If you are not sure if a list item applies to your research, read the appropriate section before selecting a response.

Materials & experimental systems

n/a	Involved in the study	
☐	☒	Antibodies
☒	☐	Eukaryotic cell lines
☒	☐	Palaeontology and archaeology
☐	☒	Animals and other organisms
☒	☐	Clinical data
☒	☐	Dual use research of concern
☒	☐	Plants

Methods

n/a	Involved in the study	
☒	☐	ChIP-seq
☒	☐	Flow cytometry
☒	☐	MRI-based neuroimaging

Antibodies

Antibodies used	Polyclonal antibodies generated bespoke for this study utilised recombinant proteins synthesised from Brugia malayi (BMRV1 capsid protein) and Onchocerca volvulus cDNA (OVRV1 glycoprotein). This work was outsourced to Alta Bioscience Ltd (Redditch, UK),

project numbers Q10640 (BMRV1 capsid protein) and Q10986 (OVRV1 glycoprotein).

Additional monoclonal antibodies were also used:
IRDye 680RD goat anti-rabbit IgG (Li-COR Biosciences, Lincoln, USA), catalogue number 926-68071, lot number D30221-0.5 Used at a dilution of 1:15,000.
Rabbit anti-mongolian gerbil IgG (H+L) (Bioss, USA), catalogue number BS-0403R, lot number BB04262953. Used at a dilution of 1:15,000.

Validation

All antibodies were tested and validated using pre-immune sera, 1st, 2nd, and last bleed serum from nematode extracts (microfilariae and adult stages for both B. malayi and O. volvulus), E. coli homogenates (isolate DE3), and insect cell line homogenates (C6/36 infected with Wolbachia endosymbiont wAlbB). These extracts were tested via Western blots, and showed that antibodies used in this study were specific to recombinant proteins only.
Further validation for OVRV1 glycoprotein was performed using immunofluorescence antibody staining with a no-antibody control. This showed no fluorescent signal in any tissues, whilst samples with the probes included showed no non-specific fluorescence in fibrous, human-derived nodule tissue.

Animals and other research organisms

Policy information about studies involving animals; ARRIVE guidelines recommended for reporting animal research, and Sex and Gender in Research

Laboratory animals

Meriones unguiculatus (Mongolian Jirds) were obtained from Charles River Laboratories and used for the maintenance of the parasite life cycle. Only male jirds were used as these show enhanced susceptibility to infections with Brugia malayi when compared to females. Individual 12-week old jirds were ordered and infected as described in the manuscript and elsewhere (A murine macrofilaricide pre-clinical screening model for onchocerciasis and lymphatic filariasis. Parasit. Vectors 7, 472 (2014).). Infections were allowed to continue, and microfilariae sampled from the jirds at monthly intervals. Jirds were culled following Schedule 1 procedures at a maximum age of 24 months, and adult parasite material obtained from the carcasses.

Female Aedes aegypti mosquitoes (Liverpool black-eye strain, obtained from the Filariasis Research Resource Reagent Center) were used for the maintenance of Brugia malayi parasites. Mosquitoes were aged 2 weeks when fed infected blood containing Brugia malayi microfilariae, and processed aged 4 weeks for infective L3-stage parasites.

Brugia malayi are not standard laboratory animals- they are lower invertebrates, and parasites of vertebrate hosts. The exact age cannot be tracked, as we cannot follow them from birth to adulthood within their host laboratory animal (Meriones unguiculatus).

Wild animals

This study did not involve wild animals.

Reporting on sex

As only male jirds were used for parasite maintenance, no sex-based analysis of the laboratory animal host was possible. This study was only interested in analysing the presence/absence and abundance of viral loads within the B. malayi parasite itself, and not whether the parasite host gender has any impact on viral loads/presence.

Field-collected samples

This study did not involve field-collected samples of parasites from animals/other research organisms.

Ethics oversight

All animal experiments were approved by the ethical committees of the University of Liverpool and Liverpool School of Tropical Medicine (LSTM), and conducted under Home Office Animals (Scientific Procedures) Act 1986 (UK) requirements (license numbers P86866FD9 and PP6173839).

Note that full information on the approval of the study protocol must also be provided in the manuscript.

Plants

Seed stocks

This study did not involve seed stocks or plants of any type.

Novel plant genotypes

This study did not involve seed stocks or plants of any type.

Authentication

This study did not involve seed stocks or plants of any type.
