# Supplementary material for: Diverse RNA viruses of parasitic nematodes can elicit antibody responses in vertebrate hosts
Source: Nat Microbiol. 2024 Sep 4;9(10):2488–505. doi: 10.1038/s41564-024-01796-6 (PMC11445058; doi:10.1038/s41564-024-01796-6)

Source Data Figure 1

Reverse-transcription PCR results of Brugia malayi *parasite RNA from FR3 laboratories. Two independent parasite replicates, with four different PCR targets against the RNA genome of Brugia malayi RNA Virus 1. Blue bounding box is the section visualised in Figure 3a.*

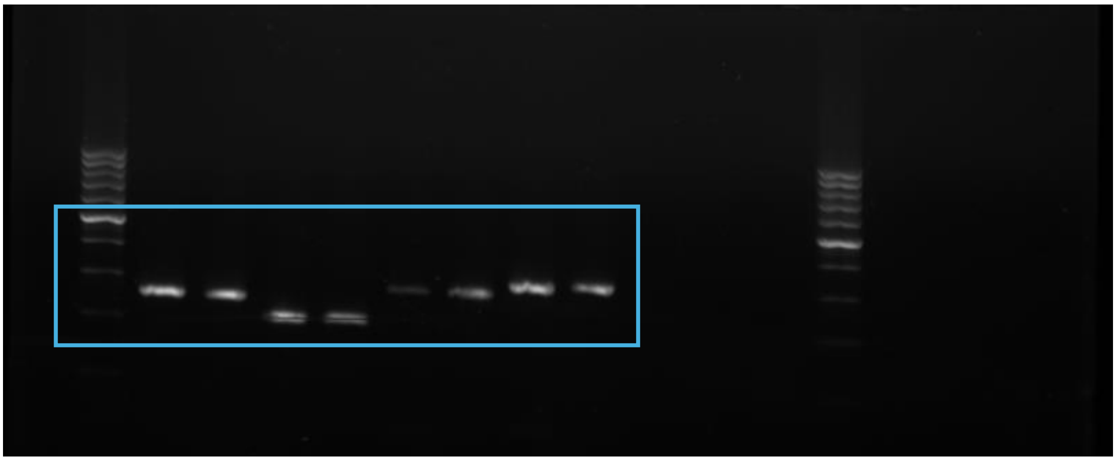

Source Data Figure 2

Reverse-transcription PCR results of Brugia malayi *parasite RNA reared at the Liverpool School of Tropical Medicine. Four independent parasite replicates, with four different PCR targets against the RNA genome of Brugia malayi RNA Virus 1. Blue bounding box is the section visualised in Figure 3b.*

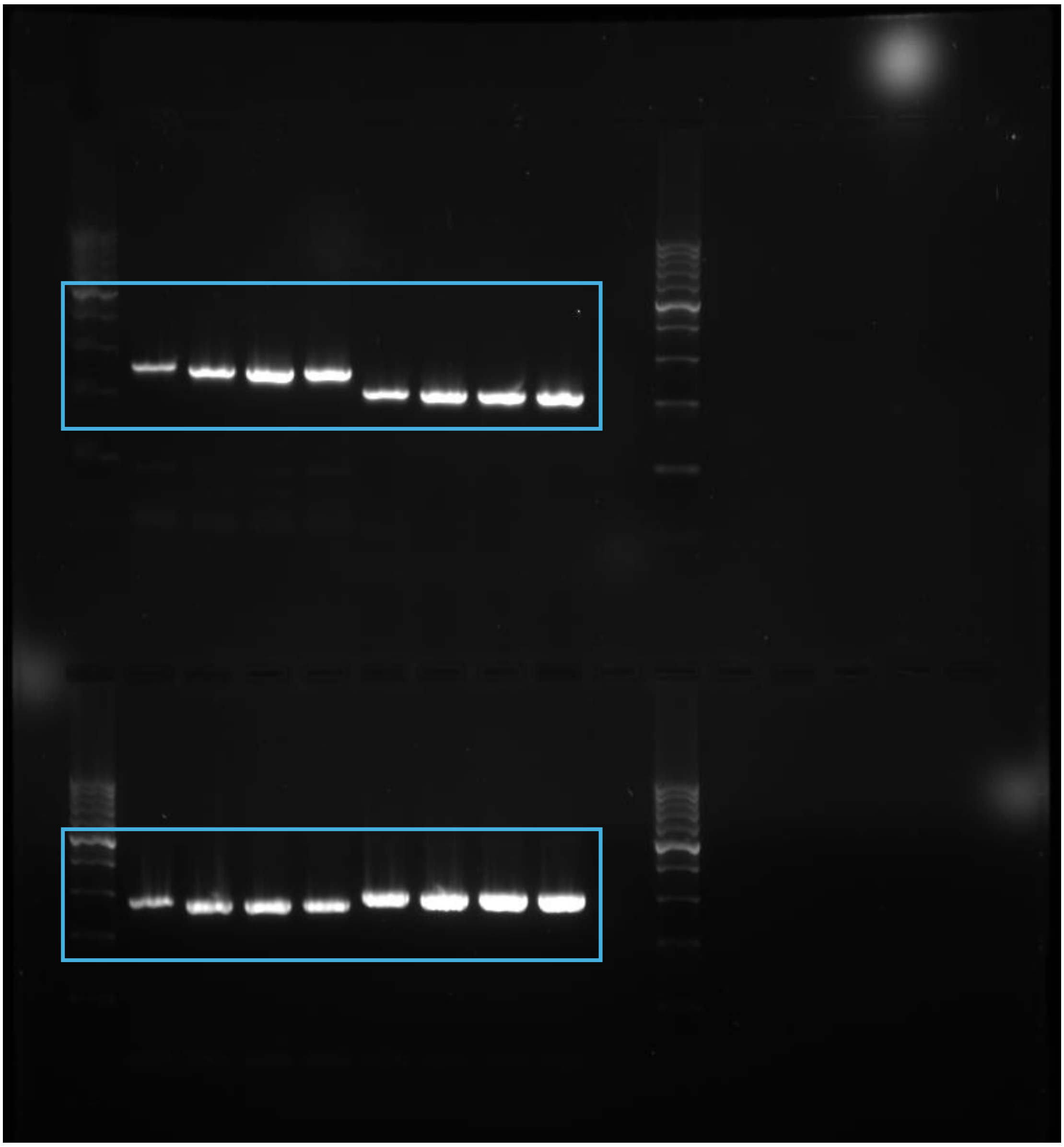

Source Data Figure 3

PCR results of Brugia malayi *parasite gDNA reared at the Liverpool School of Tropical Medicine. Four independent parasite replicates, with four different PCR targets against the RNA genome of Brugia malayi RNA Virus 1. As the target is an RNA virus genome, no amplification was identified from genomic DNA extracts. Blue bounding box is the section visualised in Figure 3c.*

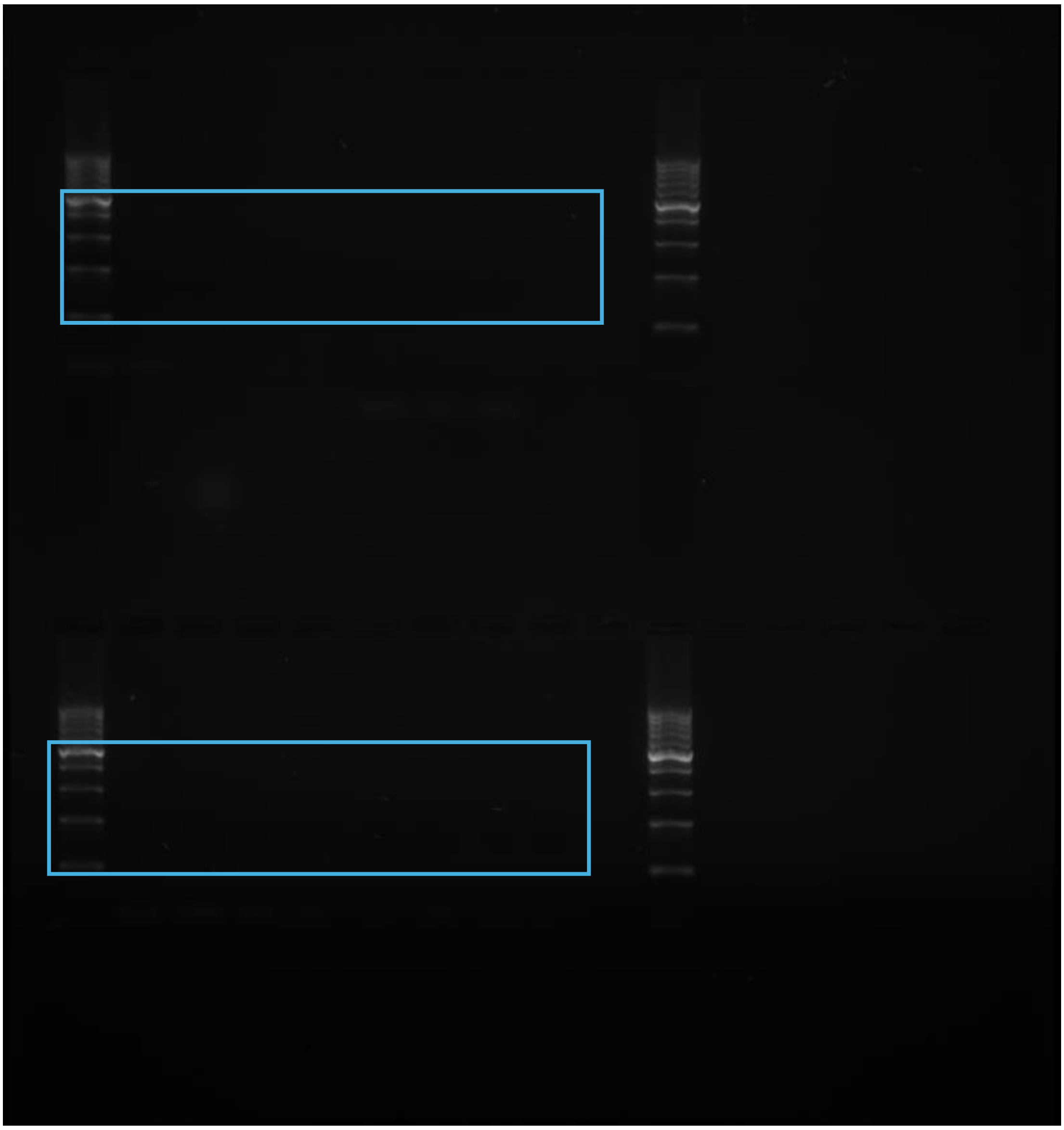

Source Data Figure 4

Western blot of Brugia malayi RNA Virus 1 capsid protein from parasite extracts. Blue bounding box is the section visualised in Figure 3e.

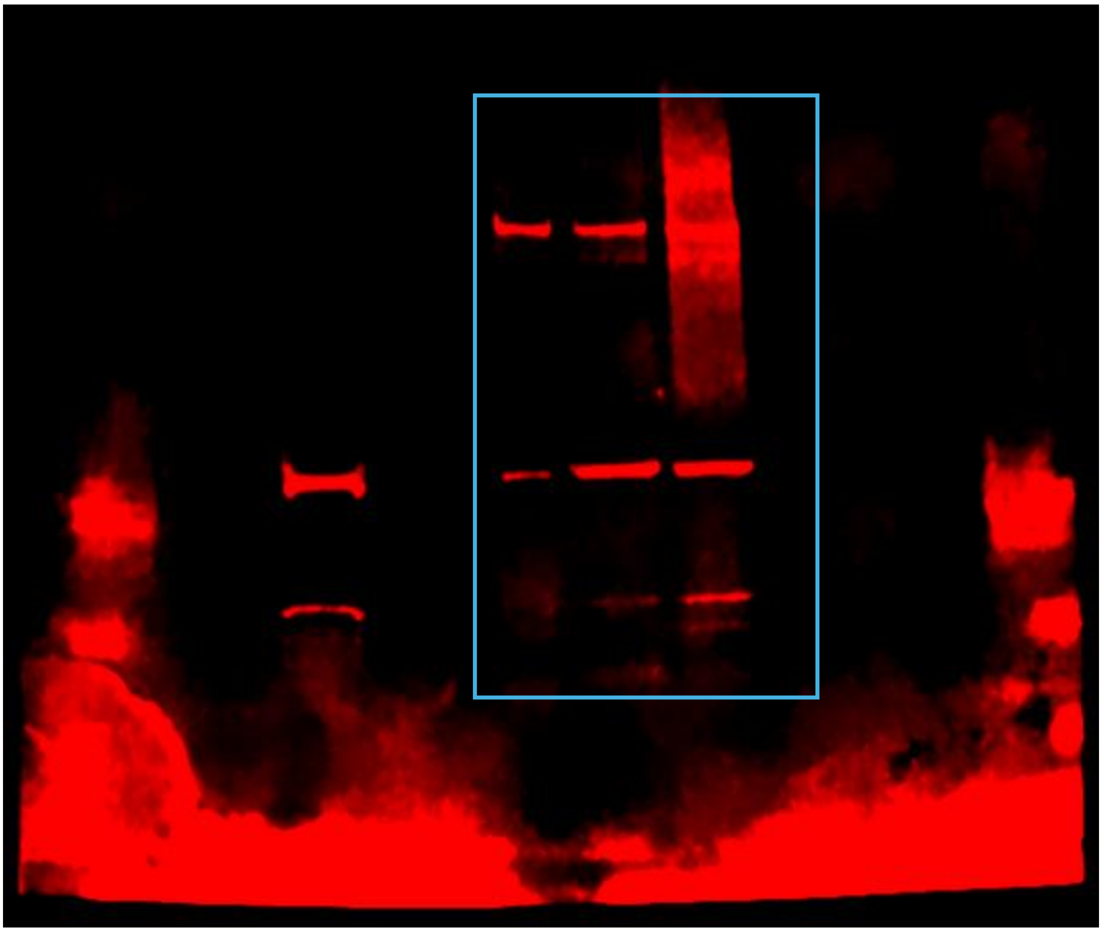

Source Data Figure 5

Reverse-transcription PCR and regular PCR results from Onchocerca volvulus *parasite cDNA/gDNA libraries stored at the Liverpool School of Tropical Medicine. Due to sample availability, only one biological replicate of* O volvulus *was available, with three additional biological replicates from* O. ochengi. *Blue bounding box is the section visualised in Figure 5a.*

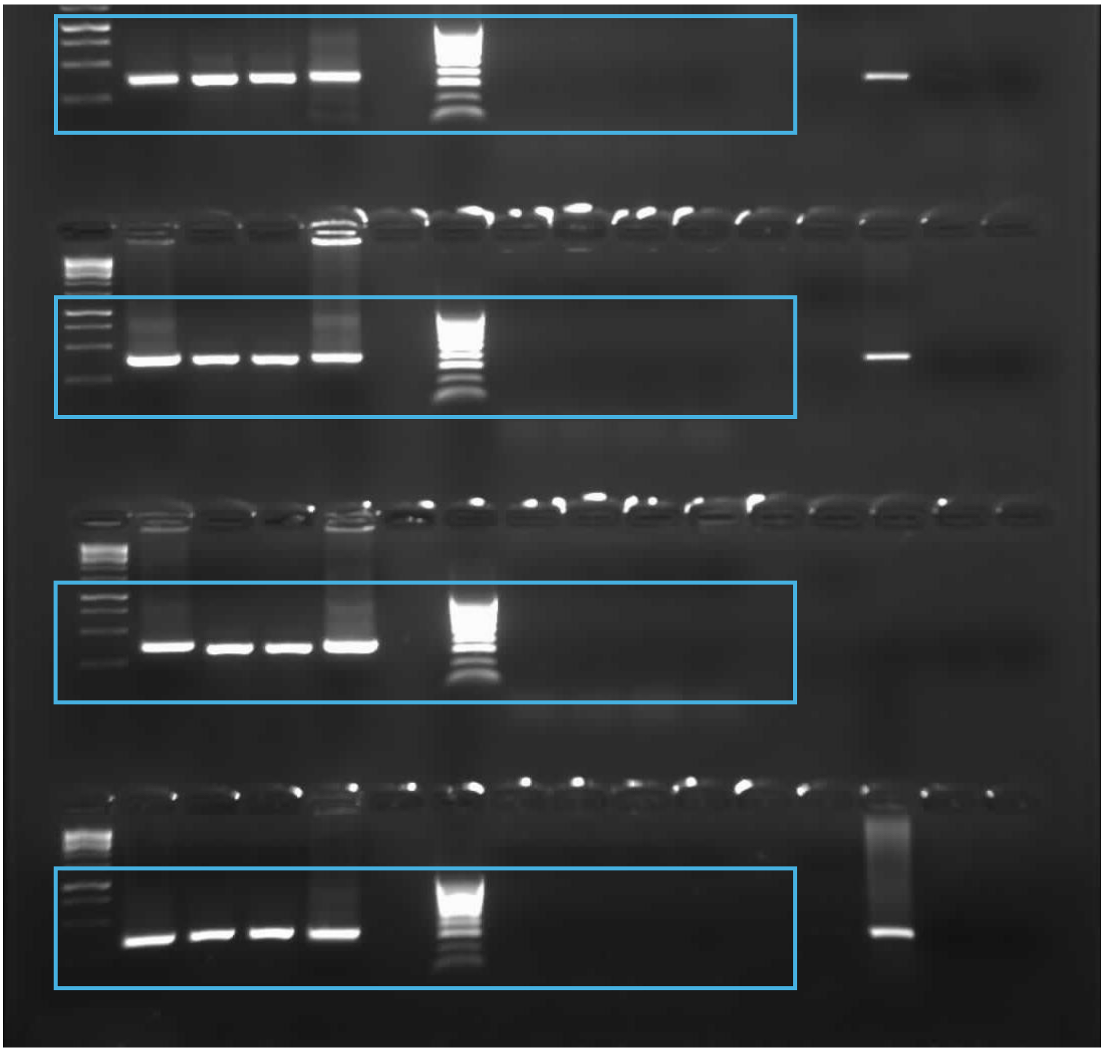

Source Data Figure 6

Western blot of Onchocerca volvulus RNA Virus 1 glycoprotein from parasite extracts. Blue bounding box is the section visualised in Figure 5b.

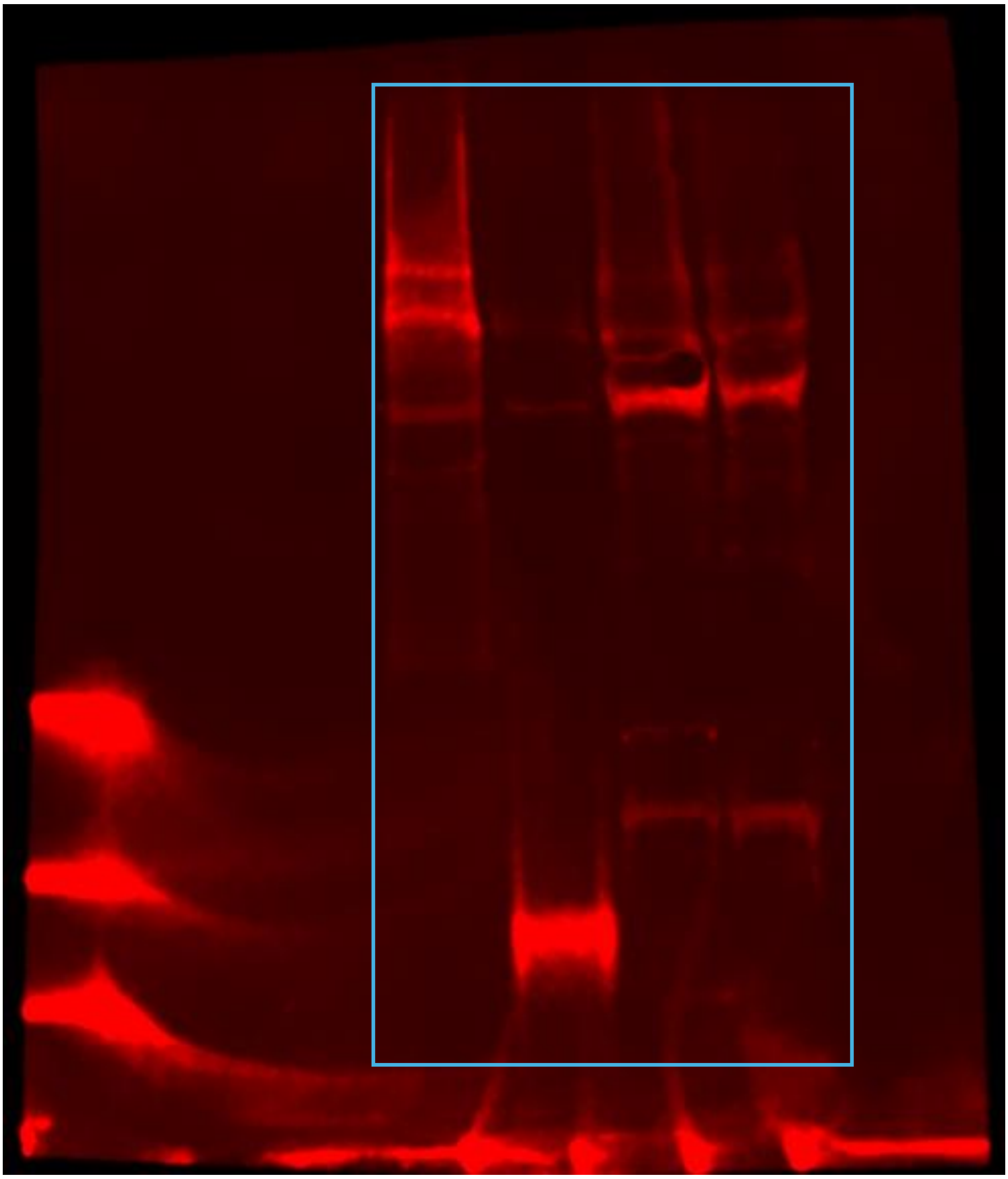

Supplement: Supplementary file 5 — Unprocessed PCR gels and western blots. [file 41564_2024_1796_MOESM5_ESM.pdf]
